# Ancient DNA at the edge of the world: Continental immigration and the persistence of Neolithic male lineages in Bronze Age Orkney

**DOI:** 10.1073/pnas.2108001119

**Published:** 2022-02-07

**Authors:** Katharina Dulias, M. George B. Foody, Pierre Justeau, Marina Silva, Rui Martiniano, Gonzalo Oteo-García, Alessandro Fichera, Simão Rodrigues, Francesca Gandini, Alison Meynert, Kevin Donnelly, Timothy J. Aitman, The Scottish Genomes Partnership, Andrew Chamberlain, Olivia Lelong, George Kozikowski, Dominic Powlesland, Clive Waddington, Valeria Mattiangeli, Daniel G. Bradley, Jaroslaw Bryk, Pedro Soares, James F. Wilson, Graeme Wilson, Hazel Moore, Maria Pala, Ceiridwen J. Edwards, Martin B. Richards

**Affiliations:** ^a^Department of Biological and Geographical Sciences, School of Applied Sciences, University of Huddersfield, Huddersfield HD1 3DH, United Kingdom;; ^b^Department of Archaeology, University of York, York YO10 5DD, United Kingdom;; ^c^Institut für Geosysteme und Bioindikation, Technische Universität Braunschweig 38106 Braunschweig, Germany;; ^d^School of Biological and Environmental Sciences, Faculty of Science, Liverpool John Moores University, Liverpool L3 3AF, United Kingdom;; ^e^Medical Research Council Human Genetics Unit, Institute of Genetics and Cancer, University of Edinburgh, Edinburgh EH4 2XU, United Kingdom;; ^f^Centre for Genomic and Experimental Medicine, Institute of Genetics and Cancer, University of Edinburgh, Edinburgh EH4 2XU, United Kingdom;; ^g^Department of Earth and Environmental Sciences, The University of Manchester, Manchester M13 9PL, United Kingdom;; ^h^Department of Research, Business and Innovation, University of West England, Bristol, BS16 1QY, United Kingdom;; ^i^Private address, Broadford, Isle of Skye IV49 9BB, United Kingdom;; ^j^The Landscape Research Centre Ltd, Malton YO17 8SL, United Kingdom;; ^k^Archaeological Research Services Ltd, Bakewell DE45 1HB, United Kingdom;; ^l^Smurfit Institute of Genetics, Trinity College Dublin, Dublin D02 VF25, Ireland;; ^m^Centre of Molecular and Environmental Biology, Department of Biology, University of Minho 4710-057 Braga, Portugal;; ^n^Centre for Global Health Research, Usher Institute, University of Edinburgh, Edinburgh EH8 9AG, United Kingdom;; ^o^Environment and Archaeology Services, Midbea Schoolhouse, Westray, Orkney KW17 2DP, United Kingdom

**Keywords:** ancient DNA, Orkney, Neolithic, Bronze Age, genome-wide

## Abstract

Orkney was a major cultural center during the Neolithic, 3800 to 2500 BC. Farming flourished, permanent stone settlements and chambered tombs were constructed, and long-range contacts were sustained. From ∼3200 BC, the number, density, and extravagance of settlements increased, and new ceremonial monuments and ceramic styles, possibly originating in Orkney, spread across Britain and Ireland. By ∼2800 BC, this phenomenon was waning, although Neolithic traditions persisted to at least 2500 BC. Unlike elsewhere in Britain, there is little material evidence to suggest a Beaker presence, suggesting that Orkney may have developed along an insular trajectory during the second millennium BC. We tested this by comparing new genomic evidence from 22 Bronze Age and 3 Iron Age burials in northwest Orkney with Neolithic burials from across the archipelago. We identified signals of inward migration on a scale unsuspected from the archaeological record: As elsewhere in Bronze Age Britain, much of the population displayed significant genome-wide ancestry deriving ultimately from the Pontic-Caspian Steppe. However, uniquely in northern and central Europe, most of the male lineages were inherited from the local Neolithic. This suggests that some male descendants of Neolithic Orkney may have remained distinct well into the Bronze Age, although there are signs that this had dwindled by the Iron Age. Furthermore, although the majority of mitochondrial DNA lineages evidently arrived afresh with the Bronze Age, we also find evidence for continuity in the female line of descent from Mesolithic Britain into the Bronze Age and even to the present day.

## Significance

The Orcadian Neolithic has been intensively studied and celebrated as a major center of cultural innovation, whereas the Bronze Age is less well known and often regarded as a time of stagnation and insularity. Here, we analyze ancient genomes from the Orcadian Bronze Age in the context of the variation in Neolithic Orkney and Bronze Age Europe. We find clear evidence for Early Bronze Age immigration into Orkney, but with an extraordinary pattern: continuity from the Neolithic on the male line of descent but immigration from continental Europe on the female side, echoed in the genome-wide picture. This suggests that despite substantial immigration, indigenous male lineages persisted for at least a thousand years after the end of the Neolithic.

Benefiting from the tail end of the Holocene climatic optimum, the British Early Neolithic spread rapidly through Britain and Ireland from the south over 300 to 400 y from ∼4050 BC (1–3). The settlers brought with them domesticated wheat, barley, sheep, and cattle, as well as knowledge of carinated bowl ceramics and causewayed enclosures (1–5), pointing to a likely source in northern France or Belgium.

The Orkney Islands, lying to the north of the Scottish mainland, flourished during the Neolithic (3800 to 2500 BC), becoming a major cultural center (6–9). Underpinned by a successful farming economy and long-range contacts, the earliest permanent settlements were constructed in wood, followed by stone-built dwellings from 3300 cal. (calibrated) BC onward (9, 10). The use of stone appears to have been a conscious design choice (9, 11, 12) and has resulted in an extraordinary level of archaeological preservation.

While recent genome-wide studies (13) have demonstrated the extent and tempo of continental migration into Britain during the Beaker period, after 2500 BC, there has so far been little or no recognition of the archaeological implications of this for Orkney. The paucity of Beakers and associated material culture in the archaeological record has been taken as an indication that the cultural and population shifts occurring elsewhere in Britain at this time had little direct impact in Orkney (8, 14–18) and indeed may have been locally resisted (6). As a result, Orkney has been seen to have developed along a largely insular trajectory during the second millennium BC.

Significant changes in funerary practice did begin to emerge at this time, and research has concentrated on funerary remains. Barrow cemeteries, some of the largest in northern Britain, appeared in Orkney around the end of the third millennium BC. These earthen mounds contained multiple burials, added sequentially and most frequently comprising cremated remains in pits or stone-lined cists (18). Flat cist cemeteries were also in use for both inhumation and cremation burials, and often graves contained the remains of several individuals, but grave goods were infrequent.

Until recently, the low visibility of settlement sites had led to the idea that this was a period of environmental and cultural recession (19). The balance has begun to be redressed through focused environmental analyses (20) and reports on settlements such as at Crossiecrown (9) and Tofts Ness (21). Opportunities to correlate settlement and funerary remains are very rare, and few sites extend across the Neolithic and Bronze Age (BA) periods, making it difficult to draw a coherent picture of change over time. In this respect, the ongoing investigations at the Links of Noltland (LoN) are providing valuable new insights.

The LoN is located on Westray, the northwesterly most island of the Orkney archipelago. The exceptional conditions have preserved extensive settlement and cemetery remains dating from at least 3300 cal. BC up to about 500 BC (22–25). While no direct overlap has yet been detected between Neolithic and BA phases of settlement, there is no evidence for a major hiatus in occupation. The BA settlement, distinguished on architectural grounds and dating from ∼2500 to 1200 cal. BC, includes three separate conglomerations of domestic and ancillary buildings, which, like their Neolithic counterparts, were spread across a contemporary farmed landscape. Built from a mix of stone and earthen banks, often arranged in pairs, they were in use until at least 1200 cal. BC. A cemetery located among these settlements, used between at least 2150 BC and 850 BC, comprised >50 burials, including >100 individuals. Both cremation and inhumation were practiced, at times contemporaneously, and multiple burials within a single grave were common. Material evidence of the “Beaker complex,” seen across mainland Britain, is scant in Orkney; a few sherds from two Beaker vessels were recovered from the wider area (19), dated to ∼2265 to1975 cal. BC, but no further pottery or recognizable artifacts have been found in association with the cemetery or settlement.

The study of ancient genomes has shown that across much of Europe, including mainland Britain, the arrival of Metal Age culture was accompanied by the introduction of new ancestry from the Pontic-Caspian Steppe and a predominance of Y-chromosomal haplogroup R1b-M269 (13, 26–31). We investigated genomic variation in the Orkney archipelago within the context of this framework. Genome-wide SNP (single–nucleotide polymorphism) capture and shotgun data were available from 21 Early Neolithic Orcadians (13, 32), but only one from the BA (13). To investigate BA Orkney, we generated whole-genome shotgun sequence data from 22 samples from the LoN cemetery and analyzed them alongside these published data. We also included new data from three Iron Age (IA) samples from the multiperiod ritual complex and cemetery site of Knowe of Skea (KoS), on the west coast of Westray, and 12 further prehistoric samples from Scotland and northern England.

## Results

We present shotgun genome data from 29 samples from prehistoric Scotland and eight from northern England: 22 from the BA LoN in Westray, Orkney, dating to ∼1400 to 1700 BC (LoN); three from the IA KoS in Westray, Orkney, dating to the first two centuries AD; one from IA Milla Skerra (MS), Unst, Shetland; one from IA High Pasture Cave (HPC), Isle of Skye in the Hebrides (33); one from Neolithic Strath Glebe (SG), also Skye; a Pictish sample from Rosemarkie Cave (RC), Black Isle in northern Scotland, dating to 430 to 630 AD; a Beaker burial sample from Low Hauxley (LH), Northumberland; three BA samples from West Heslerton (WH), North Yorkshire; two IA samples from Knapton Wold (KW), North Yorkshire; and two IA samples from Carsington Pasture Cave (CPC), Derbyshire. Whole-genome coverage varied greatly from 0.0007× to 0.8207×. We undertook genome-wide analysis on samples above 0.009×, with samples averaging 0.194×. All samples passed contamination tests (Table 1, *SI Appendix*, Table S1, Dataset S1 *A* and *B*, and *SI Appendix*, Fig. S1). We analyzed these in the context of genome data from Early Neolithic Orkney (*n* = 21) (13, 32) and Neolithic, Chalcolithic (CA), and BA Europe and 1,856 new mitogenomes from modern Orkney (*n* = 1,356) and Shetland (*n* = 500) (Datasets S1*C* and S2).

**Table 1.** Summary of ancient samples reported in this study

Sample	Site	Period	Calibrated date to 2σ	Sex	mtDNA haplogroup	Y-DNA haplogroup (2017 ISOGG nomenclature)
KD026	SG	Scotland Neolithic	―	XY	U5b2c	I2a2b-FGC29562/Y10705
KD070	LH	England EBA	2464–2209 cal. BC	XY	T2e1	R1b1a1a2a1a2c1a1n-BY575
KD003	WH	England EBA	―	XX	T2e	n/a
KD040	WH	England EBA	―	XY	T2b4h	R1b1a1a2a1a1e1b-FGC15048
KD041	WH	England EBA	―	XY	U5a1+@16192	R1b1a1a2a1a2c-S461/Z290
KD006	LoN	Orkney MBA	1622–1498 cal. BC	XY	T2a1b1a	I2a1b1a1b-A1150/Y13519
KD044	LoN	Orkney MBA	―	XX	U5b2a3b	n/a
KD045	LoN	Orkney MBA	―	XY	J1c2a	I2a1b-M423
KD046	LoN	Orkney MBA	―	XY	T2a1b1a	Undetermined
KD047	LoN	Orkney MBA	1501–1319 cal. BC	XY	H39	I2a1b1a1b-A1150/Y13519
KD048	LoN	Orkney MBA	1509–1416 cal. BC	?	H39	n/a
KD049	LoN	Orkney MBA	―	XY	H39	I2a1b1a1b1-A8742
KD050/65	LoN	Orkney MBA	1609–1437 cal. BC	XX	H39	n/a
KD051	LoN	Orkney MBA	1743–1543 cal. BC	?	Undetermined	n/a
KD052	LoN	Orkney MBA	―	XX	K1a29a	n/a
KD053	LoN	Orkney MBA	―	XY	Undetermined	Undetermined
KD055	LoN	Orkney MBA	―	XX	J1c2a	n/a
KD057	LoN	Orkney MBA	―	XY	H1n1	I2a1b1-L161.1/S185.1
KD058	LoN	Orkney MBA	1616–1456 cal. BC	XX	K1a3a	n/a
KD059	LoN	Orkney MBA	1620–1462 cal. BC	XY	T2b21	I2a1b1a1b-A1150/Y13519
KD060	LoN	Orkney MBA	―	XY	H1n1	I2a1b1-L161.1/S185.1
KD061	LoN	Orkney MBA	―	XY	K1c2	R1b1a1a2a1a2c1a-CTS24/DF13/S521
KD062	LoN	Orkney MBA	1536–1425 cal. BC	XX	U5b2a3b	n/a
KD063	LoN	Orkney MBA	―	XX	H58a	n/a
KD064	LoN	Orkney MBA	―	XY	T2b21	I2a1b1a1b1-A8742
KD066	LoN	Orkney MBA	―	XX	T2a1b1a	n/a
KD067	LoN	Orkney MBA	―	XX	H+195	n/a
KD071	KW	England IA	―	XX	H1b1 + 16362	n/a
KD072	KW	England IA	―	XX	H1b1 + 16362	n/a
CE003	CPC	England IA	758–416 cal. BC	XX	X2b4	n/a
CE004	CPC	England IA	387–205 cal. BC	XY	H10b	R1b1a1a2a1a-L151/PF6542
KD004	KoS	Orkney IA	340 cal. BC–cal. AD 4	XY	H1b	R1b1a1a2a1a2c-S461/Z290
KD042	KoS	Orkney IA	―	XX	U5a1b1a	n/a
KD043	KoS	Orkney IA	25–215 cal. AD	XY	H1b	R1b1a1a2-M269/PF6517
KD005	HPC	Scotland IA	46 cal. BC–cal. AD 202	XX	H7a1b	n/a
KD073	MS	Shetland IA	236–402 cal. AD	XY	J1b1a1	Undetermined
KD001	RC	Scotland IA/medieval	441–641 cal. AD	XY	J1b1a1a	R1b1a1a2a1a-L151/PF6542

CPC, Carsington Pasture Cave, Derbyshire; HPC, High Pasture Cave, Skye; KoS, Knowe of Skea, Westray, Orkney; KW, Knapton Wold, North Yorkshire; LH, Low Hauxley, Northumberland; LoN, Links of Noltland, Westray, Orkney; MS, Milla Skerra, Unst, Shetland; RC, Rosemarkie Cave, Black Isle; SG, Strath Glebe, Skye; WH, West Heslerton, North Yorkshire.

### Genome-Wide Variation.

ADMIXTURE analysis (Fig. 1*A*) showed that the samples from BA Orkney closely resembled other northern European BA people in their overall genome-wide profiles and were highly distinct from Neolithic Orkney samples, which resembled more our Neolithic sample from Skye and other British and Irish Neolithic samples. Neolithic samples all lacked the CHG (“Caucasus hunter-gatherer”) component (in blue) that most clearly signals admixture from Pontic-Caspian Steppe pastoralists (34). The CHG fraction in Orkney (both BA and IA) is somewhat higher (∼40%) than in other Scottish CA and EBA (Early Bronze Age) samples but within the wide range of values for England (Fig. 1*A* and *SI Appendix*, Fig. S2*A*). Modern Orcadians have an even higher fraction of the CHG component, reflecting medieval Norse settlement, estimated from modern genome-wide surveys at ∼20 to 25% (35) and ∼25 to 30% of modern Y chromosomes (36, 37). Geographical and chronological trends are portrayed more clearly in the PCA (principal component analysis) (Fig. 1*B* and *SI Appendix*, Fig. S3). LoN BA samples broadly clustered with northern and central European Bell Beaker, CA, and BA samples, and KoS IA samples fell within the same broad cluster.

**Fig. 1A and B.**
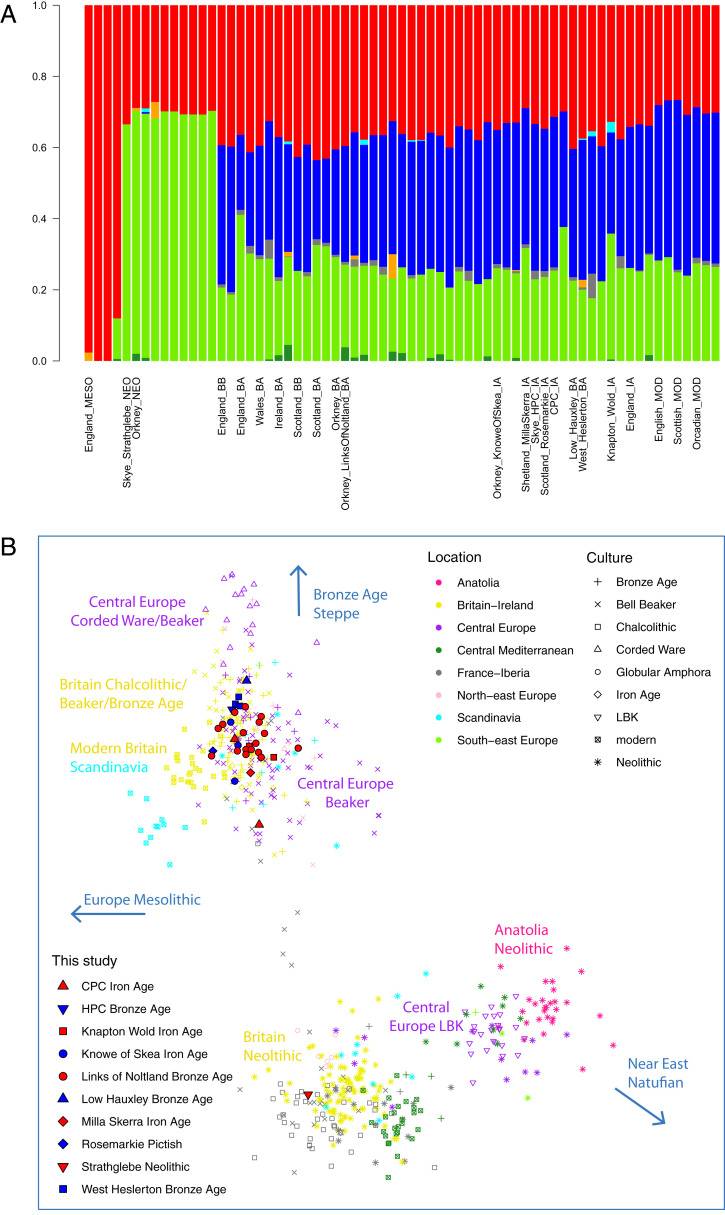
Visualization of Orkney genome-wide data in context. (*A*) Unsupervised ADMIXTURE plot (*K* = 7) of European Mesolithic, Neolithic, BA, and IA samples. The red component maximizes in the WHG, green in the ANF, and blue in the CHG; profiles to the right of each label are from the same population. (*B*) PCA showing first two principal components of European Mesolithic, Neolithic, and BA samples, projected on present-day European variation. The figure shows a zoom-in of the full plot (*SI Appendix*, Fig. S3), excluding outlier Yamnaya and Mesolithic samples. LBK, Linearbandkeramik.

**Fig. 1C.**
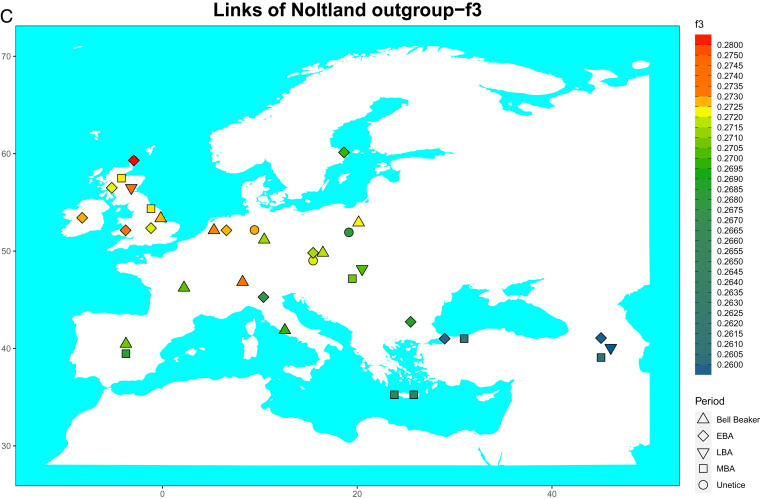
(*C*) Map displaying outgroup-*f3* statistics for the LoN samples, showing the close relationship with Bell Beaker and BA samples from the British and Irish mainland and northwestern continental Europe.

*D*-statistics quantify shared genetic drift among genomes and can thus also be used to estimate the degree of similarity among individuals. We calculated symmetry *D*-statistics by comparing potential outlier samples (as noted in the ADMIXTURE analysis) to the rest of the LoN using the form *D*(Mbuti, Test; Potential Outlier, LoN). The LoN samples consistently formed a clade, indicating that they were statistically indistinguishable from each other (*SI Appendix*, Fig. S4*A* and Dataset S1*D*). With *D*-statistics of the form *D*(Mbuti, LoN; European BA, European BA), after closest matches to the slightly older published Lop Ness BA sample from Sanday, Orkney, the most common significant similarities were with British Bell Beaker complex (BBC) samples, the Scottish BA, and Orkney KoS IA, as well as to a few continental individuals such as French BBC and the Dutch BA (*SI Appendix*, Fig. S4*B* and Dataset S1*E*). Outgroup-*f*3 statistics showed a similar pattern, with LoN closest to eastern British, Welsh, Irish, and northwest European BBC and BA samples, albeit with overlapping errors across European BBC and BA samples (Fig. 1*C* and *SI Appendix*, Fig. S5*A*). This indicates that the Orkney BA was most likely settled via the British mainland (possibly the eastern side) by people who arrived in Britain during the Beaker period.

**Fig. 2.**
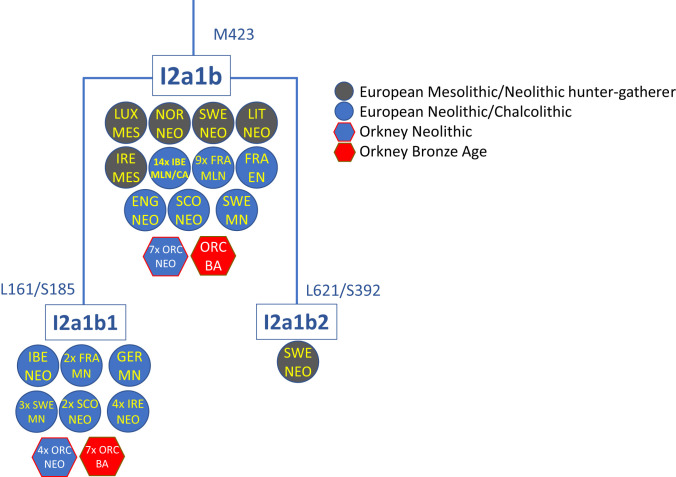
Schematic phylochronology of Y-chromosome haplogroup I2a1b-M423. For detailed branching at the tips, see *SI Appendix*, Fig. S15.

The software *qpAdm* (38) summarizes *f*_4_-statistics (which are similar to *D*-statistics) in order to estimate the direction and magnitude of gene flow, or admixture, from one population to another. We modeled admixture fractions with *qpAdm* using the three major components demonstrated by ADMIXTURE; Steppe, “Anatolian Neolithic Farmer” (ANF), and “Western Hunter-Gatherer” (WHG) (*SI Appendix*, Fig. S6 and Dataset S1*F*). The LoN comprised ∼55% of their ancestry from the Steppe, 33% from ANF, and 12% from WHG, broadly similar to published BA samples from across Britain (13).

The populations that contributed to the LoN population were likely admixtures of those three components. To identify more proximal sources for the LoN, we modeled various potential Early Neolithic versus Late Neolithic/EBA source populations (Table 2). The Orcadian BA samples could be plausibly modeled as ∼4 to 7% local Neolithic and ∼93 to 96% Scottish BBC populations, but also as 1 to 5% local Neolithic and ∼95 to 99% French BBC populations or ∼1% local Neolithic and ∼99% Danish BA populations. Despite the uncertainty indicated by the SEs, these results clearly imply very high levels of replacement of the Neolithic people by people related to continental BBC immigrants by the EBA, with only ∼5% assimilation at most of the local autosomal gene pool. However, by the time the descendants of the BBC immigrants reached Orkney, they appear to have lost their Beaker cultural affiliation, as reflected in the dearth of Beaker-associated material culture in Orkney (6).

**Table 2.** Putative BA and Neolithic ancestry of LoN MBA and Lop Ness EBA (13) samples modeled with *qpAdm*

Target	Neolithic population	Neolithic proportion	Late Neolithic/BA population	BA proportion	SE	*P* value
LoN	British Neolithic	0.039	Scotland BBC	0.961	0.032	0.079759
LoN	Orkney Neolithic	0.038	Scotland BBC	0.962	0.031	0.080413
Lop Ness	Orkney Neolithic	0.075	Scotland BBC	0.925	0.045	0.151044
LoN	British Neolithic	0.005	France BBC	0.995	0.031	0.124343
LoN	Orkney Neolithic	0.006	France BBC	0.994	0.031	0.124541
Lop Ness	Orkney Neolithic	0.052	France BBC	0.948	0.046	0.066244
Lop Ness	Orkney Neolithic	0.013	Denmark BA	0.987	0.032	0.284911

Only feasible and signiﬁcant results are displayed. The strong apparent similarity between the Orkney MBA LoN samples and the southern France BBC samples is likely not due to common ancestry but possibly due to the higher levels of Neolithic assimilation in the latter (*SI Appendix*, Fig. S2*B*); the reason for the similarity with the Danish BA is unclear.

Thus, the picture from the genome-wide analyses suggests a substantial replacement of the Orcadian population between the Late Neolithic and the BA, similar to that seen in mainland Britain (13). However, there are striking and unexpected differences between the patterns displayed by the uniparental marker systems, which can illuminate in more detail how this process took place.

### Mitochondrial DNA Variation.

Early Neolithic Orkney (*n* = 21) includes mitochondrial DNAs (mtDNAs) characteristic of the European Neolithic, suggesting predominantly settlement from the western Neolithic but with a minor contribution from the Danubian Neolithic (*SI Appendix*, Section S5). By contrast, the BA LoN suite of lineages (*n* = 20) is very different (Datasets S1*G* and S2). There are a number of minor H lineages, including H39 (four individuals), H58a, H+195, and two individuals with H1n1. There are also two with J1c2a, three with T2a1b1a—matching the EBA individual from Lop Ness (the only previously published BA Orkney sample) (13), two with T2b21, two with U5b2a3, one with K1a3a, one with K1a29a, and one with K1c2. Eight of these individuals (three of the H39 individuals, all three T2a1b individuals, one of the two U5b2a3 individuals, and the K1a3a individual) were part of a multiple burial, of which two were related (see below). The males from the multiple burial also all carried Y-chromosome haplogroup I2a1b-M423/I2a1b1-S185.

The age and geographic distribution of the clusters to which most of the BA LoN lineages belonged suggest that most of them were not inherited from the local Neolithic but arrived later. Many are associated in ancient DNA studies with continental Corded Ware Culture, BBC, or BA populations (*SI Appendix*, Section S5). For example, T2a1b1 is seen in the German Corded Ware, whereas T2b21 matches German and Czech BBC lineages. While H39 and K1c2 lineages have not been seen in published ancient DNA data, the modern lineages are restricted to northern Europe and date to ∼3000 BC and 2600 BC, respectively, again suggesting a source in the Corded Ware expansion across northern Europe at 2500 to 3000 BC. Several lineages, such as J1c2*, K1a3a, H1n1, H58a, and H+195, are harder to resolve, but their distribution is in each case consistent with a BBC arrival, although we cannot currently conclusively rule out a local Neolithic source. The IA KoS remains (*n* = 3) include two identical H1b lineages and one U5a1b1a, both of which can be attributed to either the BBC or the Corded Ware on the Continent.

The lineage most likely to date to before the Beaker Age in Orkney, seen in two LoN individuals, is U5b2a3 + 16319, which we name here U5b2a3b (Dataset S3). U5b2a3 dates to ∼8500 BC and is seen in Early Neolithic individuals from both Scotland (13) and Wales (39), and so the Orkney individuals represent potential continuity from the British Neolithic into the BA. Intriguingly, U5b2a3b is also seen in one modern individual from the British Isles (40), as well as an individual from Virginia, United States (founded as a British colony), indicating potential continuity through to the present day. Indeed, with U5b2a* found in Neolithic Orkney (32) and Scotland (13) and, notably, Mesolithic Ireland (41) and U5b2a3 itself also seen in Neolithic Ireland (41), along with the presence of U5b2 lineages in modern Orkney and Shetland (Dataset S2), it is possible that some U5b2 lineages, including U5b2a3b, may signal some of the most ancient lineages surviving in present-day Britain and Ireland, potentially even from the local Mesolithic.

### Y-Chromosome Variation.

There are 16 known Y-chromosome (Y-DNA) haplotypes from Neolithic Orkney, of which 14 appear to be well resolved (13, 32). All 14 belong to haplogroup I2a, of which seven are I2a1b-M423, four are I2a1b1-S185, one is I2a2-S33, one is I2a2a1b-CTS10057, and one is I2a2a1a2-Y3679 (the remaining two are poorly resolved I and I2). In BA LoN, even though the majority of genome-wide and female lineages most likely arrived in Britain and Orkney with the BBC or BA, all but one of the nine Y-DNA lineages belong to haplogroup I2a1b-M423, with just one belonging to R1b-M269 (*SI Appendix*, Section S6 and Dataset S1*H*). We found four distinct haplotypes within I2a1b: I2a1b-M423, I2a1b1-S185, and the more derived I2a1b1a1b-A1150 and I2a1b1a1b1-A8742.

**Fig. 3.**
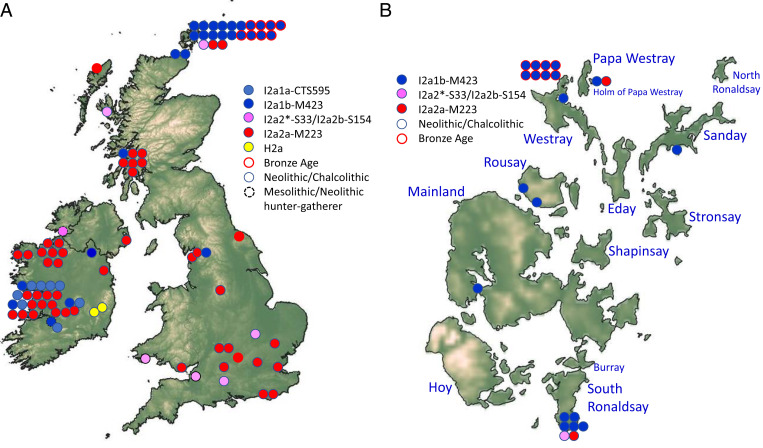
Distribution of Mesolithic and Neolithic Y-chromosome lineages, and their Bronze Age descendants. (*A*) Britain and Ireland with (*B*) zoom in on Orkney. Colors represent different Y-chromosome lineages, and distinct outlines represent the time period of the sample. Each circle represents one individual, except for Trumpington Meadows, Cambridgeshire (66), where two brothers are represented by a single circle. Maps prepared with GADM tools (https://gadm.org/data.html) (67) using data from SRTM (68).

**Fig. 4.**
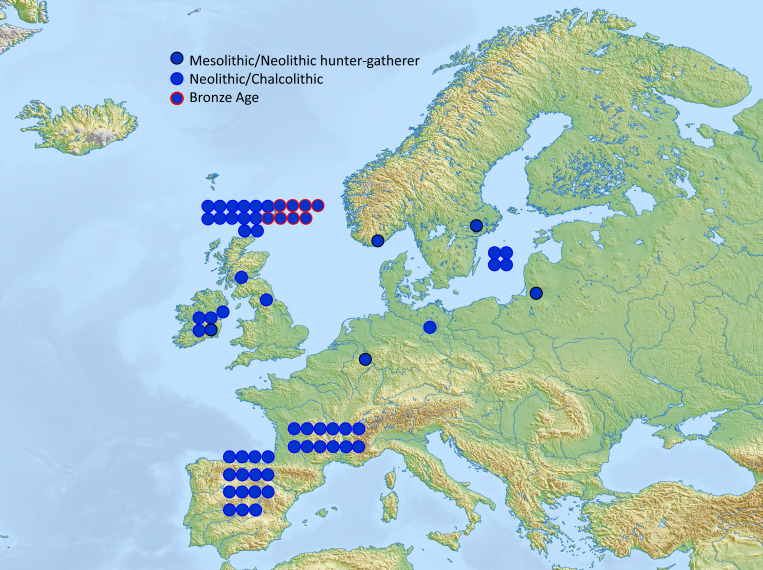
Distribution of prehistoric I2a1b-M423 Y-chromosome lineages in Europe. Each circle represents one individual carrying I2a1b. Map modified from Mapswire.com (https://mapswire.com/), which is licensed under CC BY 4.0.

This predominance of I2a1b-M423 is surprising because it is completely absent elsewhere in CA/BA Europe, where the Y-DNA landscape is heavily dominated by R1b-M269 (Figs. 2–4 and *SI Appendix*, Figs. S13–S15). For example, in a dataset of 21 BBC males from Britain, 20 carry the R1b-M269 lineage and only one I2a, which is on the distinct I2a2a-M223 lineage. If we include CA and EBA Britain and Ireland, 41 out of 43 males carried R1b-M269, two I2a2a-M223, and none I2a1b-M423.

Thus, except for the single R1b-M269 lineage, all sampled LoN BA males carried a subset of the Neolithic Y-DNA pool. These are very unlikely to have been brought to Orkney by BBC or BA migrants from further south in Britain. Not only has I2a1b-M423 not been seen in the European BBC or BA, but it was a minority lineage even during the European Neolithic. Among 389 published male genomes from the European Neolithic, only 12% (47 of them) carry I2a1b-M423, of which 40% (19/47) are from Britain or Ireland (42), and most of those in Britain are from Orkney (Figs. 3 and 4). Even in Britain and Ireland, outside of Orkney most Neolithic Y-DNA lineages belong to haplogroup I2a2-S33 or I2a2a-M223 (Fig. 3), although, curiously, our Neolithic individual from Skye belongs to the very rare I2a2b-S154, seen elsewhere only in Middle Neolithic France (43). I2a1b-M423 seems to be largely restricted to western Neolithic Britain and Ireland, where it occurs rarely alongside I2a2a-M223, as well as I2a1a-CTS595 (41), which has not yet been found in Neolithic Britain. This perhaps suggests a relict distribution, shared by Orkney, Ireland, and western and northern Britain.

A consequence is that not only was the assimilation of Neolithic male lineages very rare during the BBC spread in Britain, but assimilation of I2a1b-M423, which formed a small minority of Neolithic British mainland lineages, must have been even rarer, if it ever happened at all. We conclude that the I2a1b-M423 lineages at BA LoN had most likely persisted from the local Orcadian Neolithic and were not contributed to this population by mainland British Neolithic groups. By contrast, the two sampled males at the IA KoS site, also on Westray, belonged to the R1b-M269 lineage.

I2a1b-M423 likely arrived in Orkney with the first farmers. In the Neolithic, I2a1b-M423 was largely distributed in an arc around the Atlantic façade of Europe, from the western Mediterranean to the Baltic. Outside Britain, most I2a1b-M423 lineages are from Middle/Late Neolithic Spain and France, with one from Germany and a small number from Sweden, where, at a megalithic site on Gotland, all four genotyped males belonged to I2a1b-M423 (Fig. 4) (32). It is also present in several hunter-gatherers in northern and central Europe, including Mesolithic Ireland. This distribution, the molecular-clock age of the two major subclades (I2a1b1-S185 and I2a1b2-S392 both date to ∼7 ka) (YFull YTree version 8.06.01, 27 June 2020; https://www.yfull.com/tree/), and evidence that the ancestral lineage survives today only in Iberia (YFull tree) suggest assimilation from hunter-gatherers during the spread of the Neolithic into southwest Europe, followed by Neolithic dispersal into northwest and northern Europe, although some further assimilation in northern Europe is also possible.

### Runs of Homozygosity and Kinship.

We assessed runs of homozygosity (ROH) using the program hapROH (44). ROH profiles of BA LoN samples indicate a small effective population size but give no evidence for recent consanguinity, up to third cousin unions (*SI Appendix*, Fig. S7). HapROH estimated the effective population size to be ∼400. This is a large decrease from Neolithic Orkney and also much lower than elsewhere in Neolithic, BBC, or BA Britain and northwest Europe (*SI Appendix*, Table S2). These results suggest a small, endogamous population.

We estimated kinship using Relationship Estimation from Ancient DNA (READ) software (45), coupled with uniparental markers and the age-at-death osteoarchaeological profile. The READ analysis identified almost no evidence for close kinship. Even among the seven individuals in the multiple inhumation who passed the criteria for DNA analysis (out of 11), the only first- or second-degree relationship involved two full siblings: a brother and sister, where the former died in adolescence and the latter soon after birth. The siblings shared an identical, rare mtDNA haplotype (within H39), and the male carried the most common Y-DNA haplotype at the cemetery (I2a1b1-S185). An infant from outside of the multiple burial carried a slightly distinct lineage of mtDNA H39, but we could find no evidence of close kinship using READ (*SI Appendix*, Fig. S8*A*).

The low Y-DNA diversity and multiple sharing of rare mtDNA haplotypes both suggest a small, close-knit community, notwithstanding the relatively recent arrival (within the previous millennium) of most of the mtDNAs from overseas. However, the most significant signal remains the contrast between the autochthonous male lineages versus higher-diversity nonlocal female lineages, pointing to ongoing patrilocal marriage patterns, not only in the BA but, by inference from the persistence of I2a1b-M423, at the end of the Neolithic too. We note that although the contrast between the largely indigenous Y-DNA and the largely continental mtDNA and autosomal fraction is very striking, a level of ∼95% continental genome-wide ancestry could be achieved by the marrying out of indigenous men with immigrant women in only five generations, or 100 to 150 y, which the results suggest were followed by isolation and endogamy (*SI Appendix*, Section S3.10).

## Discussion

We have investigated genomic variation in BA and IA Orkney and compared it with the available evidence for the preceding Orcadian Neolithic, in the context of Mesolithic, Neolithic, BA, and IA variation from across Europe. Both the mtDNA and Y-DNA variation of Neolithic Orkney point to settlement primarily from the Mediterranean/Rhône/Atlantic dispersal route, via the British mainland, in line with genome-wide analyses for Neolithic Britain as a whole (13, 39). Although this process was largely one of colonization, we find some evidence for potential assimilation and survival of indigenous Mesolithic maternal lineages. The presence of an apparently ancient local branch of mtDNA haplogroup U5b complements genome-wide observations of hunter-gatherer assimilation in western Scotland (39) and Ireland (41).

This study confirms that the drastic shift in the British population in the BA, evident in both the genome-wide (13) and mtDNA patterns, also occurred in Orkney. Orkney was largely resettled from the British mainland by people of substantially recent continental ancestry. Although this demographic shift may have taken place over centuries, it was likely sustained relatively unchanged into the IA; although we have analyzed only three IA samples, they all show a similar pattern.

Unexpectedly, despite this wave of immigration, local Neolithic male lineages persisted well into the BA, at least in Westray. While we do see evidence for male newcomers, in the presence of a single R1b-M269 Y-DNA lineage (in an infant burial), the other males all carry the indigenous I2a1b-M423 lineage. This lineage survived in a single fifth or sixth century Pictish sample from Birsay, northwest Mainland (46), but is only seen in a single family (among 407 males tested) in Orkney today.

The I2a1b-M423 lineage almost vanished elsewhere in western Europe after the end of the Neolithic. None are seen in post-Neolithic European archaeological remains. It is seen at only ∼1% in modern Britain and is almost absent in most of modern western Europe, although one recent subclade of I2a1b2-S392 has undergone dramatic expansion with Slavic populations in the Balkans (Figs. 2–4 and *SI Appendix*, Fig. S13) (47).

A possible explanation can be found in the continuity, stability, and self-sufficiency of farming settlements, such as LoN. These successful household groups, while undoubtedly participating in an Orkney-wide Neolithic society, also developed strong local identities, manifested in locally variant art styles, material culture, architecture, and ritual activity. They may, for example, have pursued their own long-range contacts, as suggested, for example, by the importation of aurochs and local tomb art, distinctive within Orkney and most directly comparable with that found at Brú na Bóinne in Ireland, where patrilineal descent has recently also been inferred from genetic data (41). From a position of strength during the Neolithic, such settlements may have been better placed to mediate inward migration and to make specific choices with regards to the management of lineage.

We propose that we may be seeing the surviving remnants of well-established Neolithic household groups in BA Orkney: a number of distinct male lineages that have persisted when almost the whole of the rest of the population (and genome) has been replaced. While the archaeological signs of these groups may not have been especially ostentatious, the persistence of their lineages for at least a thousand years beyond the point when the vast majority of male lineages elsewhere in Britain were replaced by newcomers might imply a more protracted and perhaps more negotiated process of assimilation than elsewhere, as well as pointing to much less insularity than has often been assumed for the Orcadian BA (25).

There are several caveats to this suggestion. Firstly, we are describing the situation in one of the most remote parts of the Orkney archipelago and at a particular moment in time. It is a snapshot and may not be representative of Orkney as a whole. While the single Lop Ness sample (from another island in the archipelago) confirms the overall pattern of continental immigration, the individual is female and therefore provides no information on the male lineage. Further investigations can help to fill out the picture.

Secondly, there are numerous cremation burials at the site for which DNA analysis cannot be carried out. Is it possible that newcomer R1b-M269 males were mostly cremated? This seems unlikely; substantial numbers of BBC and EBA inhumation burials have been analyzed from England and Scotland, and the males carried almost exclusively R1b-M269 Y-DNA lineages. However, even if this were the case, the persistence in inhumations of the I2a1b-M423 lineage, in the face of an almost 95% replacement at the genome-wide (and probably also the mtDNA) level, remains extraordinary.

Within the European context, the Orkney BA stands in stark contrast as a location, at the northwestern extreme of the continent, where the majority of the genome was overwritten between the Late Neolithic and the end of the EBA but the male lineages somehow persisted. Even so, we can understand this phenomenon in terms of the same patrilocal marriage practices that we see throughout west Eurasia. The ancestral distribution in Orkney demonstrates deliberate marriage patterns involving local men and incoming women. This process of preferential assimilation seems likely to have continued for many generations, given the extent of replacement of the remainder of the Orcadian Neolithic genome.

The existence of a powerful and likely strongly hierarchical strand in Neolithic society has been proposed on the basis of the discovery of an incestuous first-degree union at Newgrange in Ireland (41) and was prefigured by earlier analyses of Ireland and other megalithic cultures in both northwest and central Europe (32, 48). Cassidy et al. (41) argue that it encompassed the whole of Ireland, adding that it may have incorporated the similar megalithic communities of Wales and Orkney, most likely originating in Brittany (1, 49). I2a1b-M423 is seen in both Mesolithic and Neolithic Ireland, and the main cluster seen in Late Neolithic Ireland, I2a2a1a1-M284—found in the putative elite lineage at Newgrange—matches an Orcadian Neolithic lineage from the Isbister Chambered Cairn (“Tomb of the Eagles”) on South Ronaldsay (Fig. 3 and *SI Appendix*, Fig. S13) (13). Both our data from BA Orkney and the Neolithic circumcoastal distribution of the Y-chromosome I2a1b-M423 haplogroup lend further support to this suggestion. European Neolithic society, at one extreme (but hardly peripheral) edge of its distribution, may have been patrilineal, patrilocal, and hierarchical long before the arrival of the Beaker complex and (most likely) Indo-European speech (27, 28, 31, 50).

Our data suggest that Neolithic lineages persisted within particular farming households, which, although not obviously elite, appear to have retained control of specific landholdings over many generations. This linkage of lineage with specific place is strongly suggestive of preferential inheritance along the male line. The continuity which this engendered is likely to have contributed significantly to the longevity of settlements between the third and first millennia BC. The indigenous male lineages remained in place while their people, their culture, their language, and even their genomes were transformed to resemble more and more those of the European mainland from which the newcomers had come.

Our findings both demonstrate EBA migration into Orkney and amplify the recognition that “the expansion of the Beaker complex cannot be described by a simple one-to-one mapping of an archaeologically defined material culture to a genetically homogenous population” (51). They also highlight that population influx may have occurred even where few archaeological traces have been identified. This prompts a critical reassessment of the origins of Orcadian BA practices, which have hitherto been viewed either as insular development, imitative of distant elites, or the result of gradual filtering-in of influences. The circumstances surrounding the emergence of novel monument types such as barrows and burnt mounds, for example, will need to be reconsidered.

If more widely borne out, these findings suggest that BA Orkney is likely to have seen regular and sustained migration, engaged in long-distance exchange networks, and adopted novel practices. The perseverance of Neolithic lineages—and, potentially, identities—into this period adds a further layer of cultural complexity, the implications of which remain to be fully explored.

## Materials and Methods

We describe the archaeological samples and materials and methods fully in *SI Appendix*. Briefly, we extracted DNA from 37 samples using existing protocols (33, 52, 53). We constructed and UDG (uracil–DNA glycosylase) treated next-generation sequencing libraries (42, 54, 55), pooled equimolarly, and sequenced all libraries on an Illumina HiSeq4000 (100-bp, paired-end sequencing; Macrogen). We trimmed sequence reads of adapter sequences and merged them using AdapterRemoval (version 2.1.7) (56). We mapped reads to the human reference genome (UCSC [University of California Santa Cruz] hg19) and the human mitochondrial reference genome (the revised Cambridge reference sequence or rCRS, NC_012920.1) (57) using BWA aln (Burrows–Wheeler alignment tool) (version 0.7.12-r1039) (58) and filtered for mapping quality (56, 59). We examined molecular damage patterns to establish data authenticity and levels of mtDNA and whole-genome contamination. As expected from UDG-treated samples, observed damage patterns were minor (*SI Appendix*, Fig. S9). We carried out uniparental marker analysis and molecular sex determination (60) following established methods. We used GATK (version 3.8) to call pseudohaploid genotypes at known SNP positions, which were then merged with the Human Origins dataset (61), the 1000 Genomes Project data, and realigned published ancient samples (*SI Appendix*). We investigated population relationships between newly reported samples and other ancient and modern individuals using smartPCA and ADMIXTURE (version 1.3) (62), with *D* and *f* statistics calculated using ADMIXTOOLS (63) to formally confirm relationships, and quantified admixture using *qpAdm* (34). A list of published samples we used in analyses is in Dataset S1*I*. We inferred kinship relationships using READ (45) and assessed ROH and effective population size with hapROH (44). We describe construction of Y-chromosome phylochronology for I2a and R1b-M269 in *SI Appendix*, Section S6 and Figs. S10–S16. We extracted the modern mitogenomes from the whole-genome Orkney Complex Disease Study (ORCADES) for Orkney (64) and the VIKING study for Shetland (65).

## Supplementary Material

Supplementary File

Supplementary File

Supplementary File

Supplementary File

## Data Availability

Raw sequencing reads of ancient samples produced for this study have been deposited in the European Nucleotide Archive under accession no. PRJEB46830. Modern mitochondrial genomes generated as part of this study have been deposited in GenBank, accession nos. MZ846240 to MZ848095.
