# Supplementary material for: Ancient DNA at the edge of the world: Continental immigration and the persistence of Neolithic male lineages in Bronze Age Orkney
Source: Proc Natl Acad Sci U S A. 2022 Feb 7;119(8):e2108001119. doi: 10.1073/pnas.2108001119 (PMC8872714; doi:10.1073/pnas.2108001119)
Supplement: Supplementary File [file pnas.2108001119.sapp.pdf]

Appendix: Supporting Information for

Ancient DNA at the edge of the world: Continental immigration and the persistence of Neolithic male lineages in Bronze Age Orkney

Katharina Dulias[a,b,c*], M. George B. Foody[a*], Pierre Justeau[a*], Marina Silva[a#], Rui Martiniano [d], Gonzalo Oteo-García[a], Alessandro Fichera[a], Simão Rodrigues[a], Francesca Gandini[a], Alison Meynert[e], Kevin Donnelly[e], Timothy J. Aitman[f], the Scottish Genomes Partnership[f], Andrew Chamberlain[g], Olivia Lelong[h], George Kozikowski[i†], Dominic Powlesland[a,j], Clive Waddington[k], Valeria Mattiangeli[l], Daniel G. Bradley[l], Jaroslaw Bryk[a], Pedro Soares[m], James F. Wilson[e,n], Graeme Wilson[o], Hazel Moore[o], Maria Pala[a], Ceiridwen J. Edwards[a+], Martin B. Richards[a+]

[*] These authors contributed equally to this work.
[+] Joint senior authors
[†] Deceased

[a]Department of Biological and Geographical Sciences, School of Applied Sciences, University of Huddersfield, Queensgate, Huddersfield, HD1 3DH, UK;

[b]Department of Archaeology, University of York, York, YO10 5DD, UK;

[c]Institut für Geosysteme und Bioindikation, Technische Universität Braunschweig, Langer Kamp 19c 38106, Braunschweig, Germany;

[d]School of Biological and Environmental Sciences, Faculty of Science, Liverpool John Moores University, Liverpool, UK;

[e]MRC Human Genetics Unit, Institute of Genetics and Cancer, University of Edinburgh, UK;

[f]Centre for Genomic and Experimental Medicine, Institute of Genetics and Cancer, University of Edinburgh, UK;

[g]Department of Earth and Environmental Sciences, The University of Manchester, Manchester, UK;

[h]Department of Research, Business and Innovation (RBI), University of West England (UWE), Bristol, UK;

[i]Freelance archaeologist, Broadford, Isle of Skye, UK;

[j]The Landscape Research Centre Ltd, The Old Bridge Barn, Yedingham, Malton, North Yorkshire, YO17 8SL, UK;

[k]Archaeological Research Services Ltd, Angel House, Portland Square, Bakewell, Derbyshire, DE45 1HB, UK;

[l]Smurfit Institute of Genetics, Trinity College Dublin, Dublin, Ireland;

[m]CBMA (Centre of Molecular and Environmental Biology), Department of Biology, University of Minho, Campus de Gualtar, 4710-057 Braga, Portugal;

[n]Centre for Global Health Research, Usher Institute, University of Edinburgh, UK;

[o]Environment and Archaeology Services, Edinburgh, UK.

[#]Current address: Ancient Genomics Laboratory, The Francis Crick Institute, 1 Midland Road, London, NW1 1AT, UK

Corresponding authors: Ceiridwen J. Edwards and Martin B. Richards

Emails: c.j.edwards@hud.ac.uk; m.b.richards@hud.ac.uk; tel: 44-1484-471650; 44-1484-471676.

This PDF file includes:

Supplementary text

Figures S1 to S16

Tables S1 to S3

Legends for Datasets S1A to S1K, S2 and S3

SI References

Other supplementary materials for this manuscript include the following:

Datasets S1A to S1K, S2 and S3 (separate Excel files)

Supplementary text: Contents

S1. Archaeological background

S2. Sample preparation, DNA extraction and library preparation

S3. Ancient DNA data processing and genome-wide analyses

S4. Modern mtDNA processing

S5. mtDNA analyses

S6. Y-chromosome analyses

Data availability

Raw sequencing reads of ancient samples produced for this study have been deposited in the European Nucleotide Archive (ENA) under accession number: PRJEB46830. Modern mitochondrial genomes have been deposited in GenBank, accession numbers MZ846240–MZ848095.

Supplementary text

S1 Archaeological background

The Later Neolithic period in Orkney (3200–2500 BC) witnessed a series of transformations and the construction of impressive monuments, collectively celebrated under a UNESCO World Heritage Site designation. On the Mainland, the largest island, ceremonial monuments on a grand scale were erected – the finely crafted Maes Howe passage tomb, the great stone circles of Stenness and Ring of Brodgar, and the exceptional complex of buildings at the Ness of Brodgar (1). Across Orkney, settlements grew in size and number, forming nucleated villages, such as at Skara Brae and Barnhouse (2), Rinyo on the island of Rousay (3, 4) and Links of Noltland on Westray (5). Orkney may also have been the origin of Grooved Ware, a new ceramic tradition that appeared around ~3200 BC and was adopted widely across Britain and Ireland over the next few hundred years. It has been argued that these changes were associated with a more hierarchical social order (6, 7) although other possibilities exist (1, 8). By around 2800 BC, the most ostentatious elements of this phenomenon were beginning to wane, marked by the demise of prominent settlement and ceremonial sites on the Mainland, although Neolithic traditions persisted throughout the archipelago at least into the middle of the third millennium.

Across southern Britain, a steep initial rise in the population during the earlier Neolithic was followed by a decline, coinciding with worsening climatic and environmental conditions (1). A major crash ensued at 3350 BC (9), after which the economy shifted to cattle pastoralism. New ideas, and the reorganization of social structures, led to the first phase of the building of Stonehenge. It has been proposed that these changes may have accompanied a major shift in ideology, from lunar to solar religion from matrilineal to patrilineal social organization, and an increase in social hierarchy, which predated the spread of the Beaker Complex across Europe by several centuries (10). Nevertheless, the reduced population size and economic shifts may have rendered Britain vulnerable to takeover by Continental people carrying the Beaker Complex after ~2500 BC (11, 12).

The Links of Noltland (LoN) is located on Westray, the north-westerly most island of the Orkney archipelago. It comprises a swathe of prehistoric landscape, covering some 5 ha in area, which was engulfed by sand dunes in later prehistory. Neolithic remains include a large enclosed nucleated settlement, an unenclosed settlement, and a subterranean house complex. Constructed mainly from stone and set within a farmed landscape, the settlements overlap in use and represent continuous occupation from 3300 to 2500 BC. The buildings are associated with rich domestic refuse and butchery deposits and have become well-known for the discovery of carved figurines (including the "Orkney Venus", otherwise known as the "Westray wife"), carved stone balls, artwork, grooved ware pottery (13) and aurochs remains (14).

Settlement appears to have moved around within the landscape during both the Neolithic and the subsequent Bronze Age, and there is little evidence of significant differentiation between contemporary households in either period. The evidence points to a considerable degree of continuity and stability, with change being gradual and often in response to environmental shifts. Cultivation appears to have intensified during the second millennium BC and there were broad changes in the farming regime that saw an increase in sheep (15) and innovations in soil management strategies (16). The Bronze Age remains also included a subterranean ritual structure and a well house. The appearance of steatite vessels in the houses of this period imply contact with the Shetland isles to the north.

We summarise the 37 samples newly analysed for this research below, in chronological order *(see* Table 1), and the sampling locations are shown in Fig. S1 and Dataset S1A.

1.1 *Strath Glebe: Neolithic remains from a cairn at Kilchrist, Skye (SG)*

During a wider landscape survey, which was centred around the excavation of High Pasture Cave on the Isle of Skye, Scotland, the remains of a degraded cairn were recorded, which contained disarticulated human bones and teeth (17) These were believed to be associated with the Neolithic, as two leaf-shaped arrowheads were found amongst the remains, and a tooth from the bottom of the cairn was dated to 3494–3102 cal. BC (4569 ± 39; OxA-37513). The interred remains were from disarticulated skeletons, but it was estimated that a minimum of six individuals were buried here.

We selected an upper molar tooth for ancient DNA (aDNA) analysis, sample KD026.

1.2 *Low Hauxley: A Beaker burial from Northumberland (LH)*

The cliff face to the south of Low Hauxley, on the Northumberland coast, has been eroding for the past 30 years. The main archaeological site occupies a slight natural hillock and is flanked to the north and south by separate organic sediment units, described as 'peats', which would have originated as wetlands during the Neolithic period, around the 4th millennium cal. BC (18). A team led by Clive Bonsall conducted a long-term investigation of the site between 1982 and 1988, which involved systematic excavation and extensive palaeo-environmental work (19). Evidence for human activity dating from the Mesolithic through to the medieval period was found, including the remains of a Neolithic stone-built burial mound, or cairn, from which pottery sherds and cist burials, with cremated human remains, were eroding. In addition, Mesolithic flint artefacts were found in a buried soil horizon underneath the cairn, and pottery was recovered from a later soil layer within the sand dune above the cairn. Human remains, including a complete skeleton and a Beaker, were recovered from cist burials within the cairn, which had been made by digging pits through the ground surface and constructing well-made stone boxes with capstones that would have stood proud of the ground surface (18).

We sampled a single individual from Burial 1 from Cairn 1. The individual was a young, possibly male (confirmed by the DNA analysis) adult, aged between 12 and 16, crouched and facing towards the north. He was radiocarbon dated to the early part of the Beaker period (3874 ± 32; SUERC-49872; approximately 2464–2209 cal. BC) and appeared to have been accorded particular significance as a primary or founding burial.

Two other cists contained cremated remains from two other people (Burials 2 and 3), who were not accompanied by a Beaker vessel. To date, there are no other instances where both cremation and inhumation remains accompanied by a Beaker have been reported at the same site. The tops of the three cists would have remained visible after burial and they formed the first phase of the Low Hauxley cemetery, which can be directly associated with the Beaker people.

Two individuals (Burial 3 Cairn 1, and Burial 10 Cairn 2) from the site had previously undergone radiocarbon dating and ancient DNA capture sequencing (12). The Burial 3 in Cairn 1 post-dated (3621 ± 34, OxA-5553/4) our sample, agreeing with the archaeology that Cairn 1 was built for Burial 1, further highlighting the importance of this individual, and that subsequent burials were added at a later date (18). Burial 3 in the Olalde *et al.* study (12) shows evidence of Steppe ancestry and could be modelled as an admixture of mainly incoming Beaker ancestry (~89%) and indigenous Neolithic ancestry (~11%). READ analysis indicated that these three individuals were not closely related.

We sampled from the left petrous portion of Burial 1, sample KD070.

1.3 *West Heslerton: Bronze Age burials from the Vale of Pickering, North Yorkshire (WH)*

Although dominated by a large Early Anglo-Saxon or Anglian cemetery, the first documented occupation at West Heslerton, North Yorkshire dates back to the Late Mesolithic when the area was used for flint knapping, and there is evidence that people continued to live there up into the Bronze

Age. During the Late Neolithic and Early Bronze Age (EBA), the site was used for rituals and the establishment of a field system was attempted, as well as the foundation of two Early Bronze Age barrow cemeteries (20). One of the EBA cemeteries produced an important series of Beaker vessels (21). Woodland regenerated in the area after the cemeteries went out of use during the late Bronze and early Iron Ages, but then the site was once again continuously occupied throughout the Roman and Anglo-Saxon periods, with evidence of settlement from the Late Iron Age onwards (20).

We sampled three petrous portions, samples KD003, KD040 and KD041.

1.4 *Links of Noltland: A Bronze Age cemetery in Westray, Orkney (LoN)*

Links of Noltland is a farming settlement on Westray, Orkney, dating from about 3300 BC to 500 BC and extends for ~2.3 hectares. Increased erosion towards the end of the 20th century AD, and a loss of vegetation cover, had exposed archaeological remains by the mid-1980s and conservation measures were undertaken, including rescue excavations from 2007 onwards, which uncovered a large Bronze Age cemetery.

The cemetery, containing both inhumations and cremation burials, is located in the south-eastern part of the site. The burials were arranged around a central ring ditch; no formal boundaries to the cemetery were located. Three groups of houses lay nearby, each group containing paired buildings. Two burial types are typically found in Orkney: flat cist cemeteries occasionally associated with barrows or mounds, and artificial mounds containing cists (22). The Bronze Age cemetery at the Links of Noltland appears to have been a flat cist cemetery. No mounds could be found in relation to the cemetery, but it is possible that this might be due to severe erosion in the area. Considering the position of the Noltland cemetery relative to the paired Bronze Age structures, and the possible field systems to the north of the site, the funerary area appears to have had close associations with agricultural complexes, as discussed by Øvrevik (23).

Twenty-five articulated and twenty-six disarticulated skeletons, as well as thirty-six cremation deposits, were discovered. The articulated human remains could be aged accurately. Grave goods were scarce and comprised for the most part sherds of pottery and a few pieces of worked bone. They were associated mostly with children and women as was common practice during the Bronze Age, particularly in Ireland (24). Many children were buried in stone-lined cists, while most of the adult inhumations were buried in simple sand-cut graves. Multiple burials appeared to be a common feature of this cemetery, with at least three multiple burials identified, one containing over 22 individuals. The radiocarbon dates indicate that this large tomb was reused over a protracted period, and, on current evidence, the burials were successively interred over a period of several hundred years. As the tomb was capable of being re-opened, it is possible that remains may also have been removed during this period, and the number of individuals found during excavation may not represent all of the burials originally interred. The cremations were buried in simple sand-dug pits. At other sites in Bronze Age Orkney, multiple burials began to be replaced by single-cists burials during the Bronze Age, which were then replaced by cremations. At Links of Noltland, however, it appears from radiocarbon dates that both inhumations and cremations occurred at the same time, suggesting that the two rituals were of equal importance.

We selected 25 of the excavated individuals, consisting of 16 petrous bones and nine teeth, for genome-wide analysis. All of the samples were part of the main cemetery and buried close to one another. Nine of the individuals derived from a single grave that included multiple inhumations: KD006/9293 (3285 ± 30; SUERC-35253; 1622–1498 cal. BC), KD046/9275P, KD047/9302 (3155 ± 30; SUERC-36893; 1501–1319 cal. BC), KD049/9275I, KD050/9275C, KD058/9295 (3270 ± 30; SUERC-36895; 1616–1456 cal. BC), KD062/9290 (3225 ± 30; SUERC-35261; 1536–1425 cal. BC), KD065/9291G (3245 ± 30; SUERC-35498; 1609–1437 cal. BC), and KD066/9304. Two other inhumations, KD052 and KD053, were also buried together in stone-lined cist 9414. KD059/9053 was dated to 1620–1462 cal. BC (3280 ± 30; SUERC-27901).

1.5 *Knapton Wold: An Iron Age cemetery in the Vale of Pickering, North Yorkshire (KW)*

Nearby the Anglo-Saxon cemetery at West Heslerton, two female Iron Age individuals were excavated from Arras burials at Knapton Wold (25). The female skeletons were well preserved and assumed to be related to each other (confirmed by the DNA analysis).

We sampled the petrous portion from each skeleton, samples KD071 and KD072.

1.6 *Carsington Pasture Cave: Iron Age remains from a cave site in Derbyshire (CPC)*

Carsington Pasture Cave, Derbyshire, was excavated in 1998–1999. It contained 34 human burials, consisting of 16 adults and 18 juveniles, dating from the Neolithic to Iron Age (26).

We analysed two tooth samples dating to the Iron Age: an upper M2, CE003, dating to 758–416 cal. BC (2460 ± 30; OxA-29233) and a lower right M2, CE004, dating to 387–205 cal. BC (2240 ± 24; OxA-28865).

1.7 *Knowe of Skea: An Iron Age funerary complex in Berst Ness, Westray, Orkney (KoS)*

The site of Knowe of Skea on Westray, Orkney encompasses burials and material from the Neolithic to the early medieval era, and several buildings dating to the Bronze and Iron Age. Numerous burials were recovered from this site (>100). Radiocarbon dating indicates that the burials date from at least 300 BC to 500 AD. Detailed analysis of the ages and sexes represented indicates that they comprise the remains of a local community (27). The cemetery is the largest of this period yet discovered in Scotland (28).

We sampled from three disarticulated inhumations of an adolescent, a middle-aged adult and an older adult. Of these, individual KD004 (4038) was dated to between 340 cal. BC and cal. AD 4 (2095 ± 35; SUERC-8410), while KD043 (4045) was dated to cal. AD 25-215 (1915 ± 35; SUERC-8411).

1.8 *High Pasture Cave: An Iron Age skeleton from a cave site in the Parish of Strath, Isle of Skye (HPC)*

The human remains of High Pasture Cave include a complex and intriguing assemblage of from the Scottish Iron Age. At least seven, and probably many more (>12), individuals are represented, from foetal to fully adult remains; from articulated burial to disarticulated deposits mixed with animal bones.

One unusual aspect of the finds at HPC is the age profile of the human remains. The majority have either not lived at all independently of a womb or have barely lived a few weeks or months. This is particularly true of the remains deposited right at the end of the use of this site, including one adult and four foetal to infant individuals. Although pregnancy and birth in Iron Age Atlantic Scotland no doubt frequently ended in tragedy, this relative absence of older children and adults clearly cannot represent the natural death profile of a community, and it therefore seems an inescapable conclusion that the very young were specifically selected for deposition on this site. This is particularly noteworthy as the articulated skeletons recovered from other Iron Age sites are overwhelmingly those of adults (29).

We sampled the petrous bone from a 25- to 40-year-old adult female, KD005, dating to 46 cal. BC to cal. AD 202 (1965 ± 40; SUERC-14946). For further site information, *see* Dulias *et al*. (30).

1.9 *Milla Skerra: An Iron Age settlement site at Sandwick, Unst, Shetland (MS)*

The Glasgow University Archaeological Research Division (GUARD) excavated an eroding coastal site between 2004 and 2007 at Sandwick, on Unst, in the Shetland Isles (31). During this excavation, the remains of a partly truncated building of later prehistoric date were revealed. While excavating the building, an inhumation burial was discovered that had been cut through windblown sand that sealed the building after its abandonment. The burial was radiocarbon dated to cal. AD 236–402 (1755 ± 35; SUERC-10745), which falls within the Scottish Iron Age. The closeness of the burial to an abandoned settlement appears to be a widespread practice during the later Iron Age of placing articulated bodies

into or around ruins of buildings across the north and west of Scotland. This practice suggests that the community may have seen connections between the dead and ruined buildings, linking the people to their lineages. Occasional burials along the foreshore of Sandwick are also known and, on the same beach, other remains have been found in short cists.

We sampled the left petrous bone of Skeleton 1, a 50- to 60-year-old male (sample KD073). He had been buried with grave goods, such as a polished disc of cordierite talc schist, and yellow beads on a copper-alloy spiral ring.

1.10 *Rosemarkie Cave: A Pictish skeleton from the Black Isle (RC)*

The Rosemarkie Cave Project has been investigating the archaeology of 19 caves in the southeast of the Black Isle, Scotland since 2006. First results indicated that some caves were visited or occupied during the 7th–9th centuries AD, with open-area excavations of this site uncovering iron-working activity and a *c*. 5th–7th century AD male inhumation burial (32).

The site stratigraphy and radiocarbon dating results suggested that metalworking was taking place within a group of three caves at Learnie during the early medieval period. The inhumation, recovered from Learnie 2B Cave, had been deposited in a pit excavated in sand deposits within the floor of the cave, in a dark alcove. The inhumation predated the start of the metalworking phase in the cave. However, it is possible that metalworking in the adjacent Learnie 1B Cave was contemporary with the deposition of the inhumation (further radiocarbon dates forthcoming).

Radiocarbon dates of animal bones from across the site gave an overlapping time frame of 600–941 AD. Later investigations provided radiocarbon dates from archaeological sequences of worked bone and antler from the 2nd–3rd century AD. Butchered animal bone comprised the main meat-bearing elements from at least eight cattle, along with two bones from a horse. The bones from a large plaice had also been included in this bone group, which had been deposited above the cranium of the human inhumation. These may have comprised the remains from a feasting deposit.

The male inhumation burial was found below a post-medieval cobbled floor and midden deposits in a dark alcove, and comprised the well-preserved remains of a young male, who had suffered severe, multiple traumas to the head. The grave was unmarked, with stones weighting down the limbs, and butchered animal bones placed over the position of the head. The burial is quite remarkable as its position, and the condition of the individual at the time of death, are unusual. The skeleton was aligned in a northwest–southeast position with the skull pointing to the southeast. The arms were by the side of the torso, while the lower limbs/feet were in a cross-legged position. Forensic analysis showed that the individual was very healthy and of a robust build. He died due to multiple trauma wounds to his skull, caused by more than one weapon. A fragment of the left rib provided a radiocarbon date of cal. AD 441–641 (1508 ± 30; SUERC-70721).

The unusual position and setting of the burial suggested a deliberate interment. The stones that were placed on top of the body may have represented an attempt to hold down the body after death, with the animal bones representing an offering, which may indicate a ritual act. The selection of the cave may have formed an important part of the burial process. Given the date range of the early 5th–7th centuries AD, the burial could potentially represent Pictish, Viking, early Christian or other influences, given the cultural milieu of the time in this region. Further to the forensic analysis of the burial, facial reconstruction was also undertaken (32).

We sampled the petrous bone of the Rosemarkie skull from the intact cranium (sample KD001).

S2 Sample preparation, DNA extraction and library preparation

2.1 *Sample preparation and DNA extraction*

We carried out the sampling at the Ancient DNA Facility at the University of Huddersfield under dedicated clean-room conditions. We wore full body suits, hairnets, gloves and face masks throughout the drilling, extraction and library preparation processes. We cleaned all tools and surfaces with LookOut® DNA Erase (Sigma-Aldrich), as well as using bleach, ethanol and long exposures to UV light.

We decontaminated the bone and tooth surfaces by UV radiation for 30 minutes on each side, followed by cleaning with 5µm aluminium oxide powder using a compressed air abrasive system. We excised the densest part of the petrous bone (33) using a circular saw and sampled the teeth by cutting off the crown. We obtained bone powder from the petrous, and tooth powder from the tooth roots, by crushing the excised portions in a MixerMill (Retsch MM400) at 30Hz/s for 30 seconds. We extracted DNA from ~150 mg of the resulting powder following the protocol by Yang *et al.* (34), with modifications by MacHugh *et al.* (35). We included blank controls throughout the extractions, library preparation and amplification reactions to monitor for possible modern DNA contamination at every stage of the process.

2.2 *Library preparation and sequencing*

We constructed next-generation sequencing (NGS) libraries from DNA extracts using the method of Meyer and Kircher (36), with modifications outlined in Dulias *et al.* (30). All libraries were UDG-treated, in order to increase the overall coverage. After final amplification, we analysed the libraries using an Agilent 2100 Bioanalyzer High Sensitivity DNA kit, and pooled them equimolarly, before sending them for 100-bp paired-end sequencing on an Illumina HiSeq4000 (Macrogen, South Korea). We sequenced twelve libraries per lane. In addition, we prepared dual-indexed libraries from samples KD001, KD003, KD004, KD005 and KD006, which were sent for 100-bp single-end sequencing on a HiSeq2500 (NBAF Liverpool, UK).

S3 Ancient DNA data processing

3.1 Primary DNA processing

We assessed the sequencing quality using FastQC v.0.11.5 (37). We trimmed the resulting NGS reads using AdapterRemoval v.2.1.7 (38), thus removing the adapters from the read pairs, trimming Ns, removing low quality bases and merging the reads. We used BWA.aln v.0.7.12 (39) to map reads to the revised human mitochondrial Cambridge Reference Sequence (rCRS), NC_012920.1 (40) – and the human reference genome (UCSC hg19/GRCh37), filtering by base quality 15 and disabling seed length as recommended for aDNA data (41), as well as using -n 0.01 and -o 2. We merged BAM files by sample using MergeSamFiles from Picard tools version 2.9.2 (https://broadinstitute.github.io/picard/). We used SAMtools v.1.3 (42) to sort and filter reads for mapping quality 20, and to remove PCR duplicates. We used qualimap v.2.2.1 (43) to assess the quality of the BAM files. Sequencing information, along with contamination results, can be found in Dataset S1B.

To authenticate the retrieved data as genuinely ancient, we assessed damage patterns using mapDamage v.2.0 (44). We observed the presence of C-to-T and G-to-A transitions, consistent with ancient DNA, although, as expected for UDG-treated samples, we observed only minor damage patterns (Fig. S9). As well as showing damage patterns consistent with ancient DNA, qualimap indicated that the samples also had low endogenous content and small fragment size – again, typical ancient DNA characteristics. Furthermore, we compared the mtDNA haplotypes of the researchers working in the ancient DNA facility to the ancient newly generated samples and all belonged to distinct haplogroups, excluding the possibility of laboratory-based cross-contamination.

We also applied two distinct contamination tests, both of which consistently indicated very low levels of contamination (Dataset S1B). Firstly, we estimated the level of whole-genome contamination using verifyBamId (45) with the 1000 Genomes Project vcf, following Günther et al. (46), Sánchez-Quinto et al. (47) and Schiffels et al. (48) (Dataset S1B, column L)

(ftp://ftp.1000genomes.ebi.ac.uk/vol1/ftp/release/20130502/ALL.wgs.phase3_shapeit2_mvncall_integrated_v5b.20130502.sites.vcf.gz). Contamination estimates were all very low, except in the case of KD053 (15% with verifyBamID), which included very few SNPS (<1000 overlapping the Harvard 1240K set) and was not included in any downstream analysis. Secondly, we further tested for mtDNA contamination using schmutzi, following default guidelines and reference panels (49); although, in this case, the deamination levels in many samples were too low for schmutzi to operate, due to the UDG treatment (Dataset S1B, column M). Contamination estimates were again consistently very low, except for KD066, where we detected no contamination with verifyBamID, but schmutzi suggested 20%; again, the number of SNPs from this sample was very low (<6000 overlapping the 1240K set) and it was not included in further analyses. All other samples were below 3% with verifyBamID, except for KD058 (3.5%), with similar results from schmutzi where these could be obtained.

3.2 *Sex determination*

We determined the genetic sex using a script published by Skoglund *et al.* (50) and, in cases where the sex could not be assigned using this method, we plotted karyotype graphs and used the Skoglund score (51). Results are in Dataset S1B.

3.3 Mitochondrial haplogroup assignment

We aligned filtered samples to the rCRS and assigned them to haplogroups using Haplogrep v2.1.19 (52), which follows the nomenclature in PhyloTree (Build 17, February 2016) (53). We checked all missing, private and heteroplasmic mutations that were detected by HaploGrep, using IGV version 2.4.16 (54).

3.4 Y-chromosome haplogroup assignment

We determined Y-chromosomal haplogroups for males using pathPhynder (https://github.com/ruidlpm/pathPhynder), supplemented with an in-house script, as detailed below (S6).

3.5 *Reference panels*

We used GATK (Genome Analysis ToolKit v.3.8 (55) pileup to call samples as pseudo-haploid. We only called bases if they were biallelic and had a minimum base quality of 30. We randomly called a base covering the position, and the allele was presumed to be homozygous.

We assembled three panels for population analysis:

Panel 1 was called from the 1240K array (https://reich.hms.harvard.edu/datasets) and merged with realigned ancient samples (Dataset S1I) (11, 12, 46-48, 56-93).

Panel 2 was the Human Origins panel combined with Panel 1, comprising 594,924 overlapping SNPs.

Panel 3 was called against the 1000 Genomes Project panel, which was filtered to keep only biallelic autosomal SNPs with a minor allele frequency of >1% (1,2149,279 SNPs).

We excluded samples that had less than 10,000 SNPs in common with any panel from further analysis. When two or more samples were related, the sample with the highest number of SNPs was used when analyses were carried out on a group level.

3.6 *Estimating kinship*

We used Relationship Estimation from Ancient DNA (READ) software (94) to infer kinship. This software can identify four levels of relatedness: “identical twin/same individual”, “first degree”, “second degree”, and “unrelated”. first-degree relationships are characterized by either parent-offspring or a full sibling relationship, whereas second-degree connections are represented by half-siblings, grandparent-grandchild, aunt/uncle-niece/nephew, or double cousins. We conducted READ on Panel 3, as it had the greatest number of SNPs and, therefore, greater potential numbers of overlapping SNPs between samples. We repeated the READ analysis using Panel 1 to double-check

results. We then combined the READ results with the individual's age-at-death, genetic sex and uniparental haplotype to determine familial relationships.

We found only one instance of first- or second-degree relationship: two full siblings: a brother and sister, where the former died in adolescence and the latter died around birth. The siblings shared an identical, rare mtDNA haplotype (within haplogroup H39), and the male carried the most common Y-DNA haplotype at the cemetery (I2a1b1-S185). An infant from outside of the multiple burial carried a slightly distinct lineage of mtDNA haplogroup H39 but we could find no evidence of close kinship using READ.

3.6.1 *Kinship within the Bronze Age cemetery from Links of Noltland*

We identified two samples, KD050 and KD065, as "identical twin/same individual". As these samples came from alternate petrous bones in the same context, they most likely belonged to the same individual and, henceforth, they were merged and are referred to as KD050/KD065. It was clear that KD049 and KD050/KD065 shared a first-degree relationship (Fig. S8A). As both died below reproductive age, and they shared a mitochondrial haplotype, we interpreted them as full siblings: brother and sister, with the latter dying around birth. As KD049 had the greater number of SNPs, KD050/KD065 was not included in analyses assessing LoN as a group.

3.6.2 *Kinship within the Iron Age cemetery from Knowe of Skea*

We found that two of the adults from the Knowe of Skea (KD042, KD043) shared a second-degree relationship, but did not share a mitochondrial haplotype (Fig. S8B). KD043, had the greater number of SNPs and therefore was included in analysis of KoS as a group.

3.7 *ADMIXTURE*

We conducted an unsupervised analysis using ADMIXTURE v.1.3 (95) on Panel 2. We pruned the panel for linkage disequilibrium using PLINK version 1.90 (96), using parameters --indep-pairwise 200 25 0.4, resulting in 351,826 SNPs remaining. We ran ADMIXTURE in 20 replicates from K = 2 to K = 14 with different random seeds, using the --cv to calculate the cross-validation error (CV). We display ADMIXTURE results at K=7 as it had the lowest CV error in which components relating to the Iranian Neolithic, western hunter-gatherers and Anatolian Neolithic were maximised (Fig. S2).

The ancient individuals analysed in this paper comprised three main components, which are respectively maximised in European Mesolithic populations ("Western hunter-gatherers", WHG), Natufian hunter-gatherers and Early Neolithic farmers from the Levant and Anatolia ("Anatolian Neolithic farmers", ANF), and the Iranian Mesolithic/Neolithic and Caucasus hunter-gatherers (CHG). A marked difference could be seen between the Orkney Neolithic and Bronze Age LoN with the introduction of the CHG component, providing clear evidence for substantial gene flow into the archipelago. The Orkney Bronze Age genomes were broadly similar to other British Bronze Age groups, both those newly presented in this study and those previously published.

3.8 *Principal component analysis*

We undertook principal component analysis (PCA) using a subset of Panel 2 of West Eurasian samples, comprising 893 modern individuals from 64 populations. We projected our data onto the PCA using Smartpca of the EIGENSOFT package (97, 98), and using the parameter "lsqproject:YES".

The newly presented samples sit within the modern variation of West Eurasians. The Strath Glebe individual plots with other Neolithic individuals and modern Sardinians. The other samples in this study plot with modern western Europeans and within the range of published British Bronze Age samples, on a cline between Anatolian Neolithic and Yamnaya Samara individuals. Some overlap can be seen between modern British and the newly reported ancient individuals (Fig. S3).

3.9 *Formal test of admixture implemented with* f*-statistics and* D*-statistics*

We calculated *f*-statistics and *D*-statistics as formal tests of admixture, in order to confirm the relationships between our new ancient samples and the published ancient data. We visualized this in PCA and ADMIXTURE analysis using Panel 1. We used the ADMIXTOOLS package (58) to assess the significance with a block jackknife of 5cM in size.

We considered statistics to be significant if they had a *Z*-score of greater than 3, which corresponds to a *p*-value of <0.001. We used the same panel for these formal tests of admixture as were previously used for the PCA and ADMIXTURE analysis. Where tests required an outgroup population, we used the modern Mbuti population of Central Africa, from the SGDP, given that no level of Eurasian admixture has been detected in this population (92, 99).

3.9.1 *Outgroup* f3-*statistics*

We used outgroup *f3*-statistics to test the relationships between the ancient Orkney genomes and other ancient populations and individuals, with qp3Pop from ADMIXTOOLS. We constructed the outgroup *f3*-statistics in the form *f3*(Mbuti; This Study, X), where "This Study" refers to our newly reported samples and "X" refers to other ancient populations. We repeated this test for groups and individuals to ensure that there were no outliers. The magnitude of the resulting *f3*-statistic is a measure of the amount of genetic drift shared between Orkney LoN and X since their split from the outgroup, Mbuti.

The results for the outgroup-*f3* in the form *f3*(Mbuti; Orkney_LoN, X), where Orkney_LoN was the Bronze Age population from the Links of Noltland, are displayed in Figs. 1C and S5A. Orkney_CA_EBA from Lop Ness has the highest genetic affinity to the LoN population. This sample is Bronze Age, but earlier than the cemetery at LoN, dating to 1950–1496 cal. BC (there are two barely overlapping radiocarbon dates from Lop Ness (12, 100). Populations showing relatively high Steppe (CHG) and hunter-gatherer (WHG) ancestry also showed close genetic links to the LoN population. British and northern European populations showed a greater affinity to the LoN than southern European populations. The Strath Glebe Neolithic individual showed greatest affinity to other Scottish Neolithic individuals, then to Neolithic individuals from Britain and Ireland, and then to Middle Neolithic individuals from western Europe (Figs. S5B and S5K).

We could not draw very precise conclusions from the other Beaker/Bronze Age (Low Hauxley, West Heslerton) and Iron Age (Knapton Wold, Carsington Pasture Cave, Knowe of Skea, High Pasture Cave, Rosemarkie, Milla Skerra,) samples, possibly due to lower coverage. In general, they display an affinity to western and northern European Bronze Age populations (Fig. S5C-J).

3.9.2 D-*statistics*

We calculated *D*-statistics using qpDstat from the ADMIXTOOLS package. To determine whether the LoN formed a single population, we tested samples that appeared distinctive in the PCA and ADMIXTURE using *D*(Mbuti, X; Outlier sample, all other LoN), where X is a published ancient population. No significant ($|Z|>3$) results were produced, suggesting that LoN forms a single population (Fig. S4A, Dataset S1D).

To identify genetic affinity between the samples in this study and other ancient populations, we employed *D*-statistics in the form of *D*(Mbuti, Test; popA, popB), where Test refers to a group from this study and popA and popB refer to other ancient populations (Dataset S1E). We considered the results only if the test used at least 10,000 SNPs.

The PCA, ADMIXTURE and outgroup-*f3* statistics showed that the Links of Noltland appeared to be genetically broadly similar to other northern and central European Bronze Age populations, which also comprised European hunter-gatherer, Anatolian Neolithic and Bronze Age Steppe components (WHG, ANF and CHG). *D*-statistics tests showed that the Neolithic samples always formed a clade to the exclusion of the Orkney_LoN group, thus showing an admixture event after the Neolithic. To test for the Bronze Age populations to which LoN had greater affinity, we used *D*-statistic tests in the form of

D(Mbuti, LON; BA1, BA2), where BA refers to a Bronze Age population. Similar to the outgroup-*f3*, the LoN population displayed a high affinity to the published Orkney_CA_EBA individual from Lop Ness (Fig. S4B).

The Strath Glebe individual had the greatest affinity with Neolithic populations from Britain and Ireland (Dataset S1E).

We compared *D*-statistic tests for the Beaker/Early Bronze Age populations (Low Hauxley, West Heslerton) and the Iron Age (CPC, HPC, Knapton Wold, Knowe of Skea, Rosemarkie) to contemporary populations from across Europe. These tests revealed greater gene flow with northern Bronze Age populations (Germany, Netherlands) than those from Iberia and southeast Europe (Dataset S1E). Again, more precise results may be limited by low numbers of overlapping SNPs.

3.9.3 qpAdm *and* qpWave

We used *qpAdm*, from the ADMIXTOOLS package, to estimate admixture proportions that contributed to each of the samples (Dataset S1F). We used a set of nine "right" populations (*i.e.* the outgroups): Mota, Ust'-Ishim, Mal'ta, Villabruna, Mbuti, Papuan, Onge, Han and Karitiana. We employed the option "allsnps: YES".

Following the ADMIXTURE results, we modelled Strath Glebe as a combination of Anatolian Neolithic and western hunter-gatherer populations (74% ANF, 26% WHG) (Dataset S1F). Comparison of autosomal and X-chromosomal ancestry proportions produced negative *Z* scores, suggesting that ANF males might have admixed with WHG females (Dataset S1J). However, Strath Glebe's mitochondrial U5b2c lineage was widespread across Europe in the Mesolithic (including several in the southeast), suggesting it may have been brought from southeast Europe (albeit not Anatolia). It was also found in Ireland in the Neolithic, in the same individual as an I2a2a Y-chromosome lineage (101). On the other hand, Strath Glebe's I2a2b Y-chromosome is extremely rare in published aDNA but has been seen in one Mesolithic individual from Ireland (101), so that the uniparental evidence points more to the opposite pattern (i.e., incoming females and local males). Moreover, Sánchez-Quinto *et al.* (47) previously demonstrated the opposite pattern in Scottish Middle Neolithic individuals (three of which are from Orkney, while the other is from northeast Scotland), but did not find sex-biased admixture in the 51 published British Neolithic individuals of Olalde *et al*. (12).

We also ran three-way *qpAdm* analyses (with Russian Samara Yamnaya, Anatolian Neolithic and WHG as source populations) for each sample individually from the LoN (total n = 16), KoS (n = 3), WH (n = 3) and previously published Early (n = 9), Middle (n = 4) and Late (n = 5) Scottish Bronze Age populations (Fig. S6). Small differences were evident between the Orkney and Scottish Bronze Ages, with the LoN having a smaller WHG component and higher ANF and Steppe components. This difference was maintained when LoN were grouped together for the analysis (Dataset S1F).

We modelled all other populations as an admixture of ANF, WHG and Samara Yamnaya (to represent Bronze Age Steppe ancestry), to indicate the overall contribution of the three distal sources. The presence of Steppe ancestry again suggests that Orkney experienced migration during the Bronze Age. LoN and KoS (analysed both individually and collectively) comprised 55% and 58% Steppe ancestry respectively, similar to results seen for Beaker samples elsewhere in eastern Britain (12).

We then modelled the LoN as a two-way admixture of more proximal putative sources. We modelled LoN as a combination of various European Bronze Age populations and the Neolithic population from either Orkney, the rest of Scotland, or the whole of Britain (significant feasible results are displayed in Table 2). The results indicated a near complete replacement of the earlier Neolithic population.

To investigate potential genetic continuity from the Bronze Age LoN to the Iron Age Knowe of Skea, we undertook several further tests. In several *D*-statistic tests an affinity could be seen between LoN and KoS in comparison to other European Bronze Age populations; *D*(Out, LoN; European BA, KoS) and *D*(Out, KoS; European BA, LoN) (Dataset S1E). These tests also produced several insignificant results

(|Z|<3), potentially as a result of SNP coverage. KoS and LoN could, however, be shown to form a clade to the exclusion of other European Bronze Age, *i.e. D*(Out, European BA; LoN, KoS) (Dataset S1K). These *D*-statistics therefore demonstrated a close similarity between the two populations.

To test whether the Knowe of Skea population could be modelled as a continuation of the Links of Noltland population we employed both *qpWave* (ADMIXTOOLS) and *qpAdm*, both of which demonstrated that KoS could be modelled as descending solely from the LoN. As an extra check, we employed two-way *qpAdm* modelling of KoS as a combination of a Scottish Bronze Age source (LoN, Scotland_CA_EBA or Scotland_LBA) and a European Bronze Age population. We added the Anatolian Neolithic, Samara Yamnaya and Neolithic populations from England, Orkney, Germany (LBK) and Iberia to the "right" populations. The only successful results were those which included LoN, where LoN contributed the vast majority (or all) of the total ancestry. We show only the significant results in Table S1. The very small contribution from other populations is further evidence that a high degree of genetic continuity occurred between the LoN and KoS.

We modelled the other Bronze and Iron Age British samples as a three-way combination of Yamnaya Samara, ANF and WHG, with the Yamnaya contributing approximately half the ancestry. This is similar to patterns identified in Olalde *et al.* (12). We did not test for proximal ancestry for these samples due to their low number of SNPs (Dataset S1F).

3.10 *Runs of homozygosity*

We calculated runs of homozygosity (ROH) using the default parameters of hapROH (102) for samples with over 400,000 SNPs: the Strath Glebe Neolithic individual, four LoN (KD049, KD060, KD061, KD064), 1 KoS (KD004) and the Rosemarkie individual. HapROH is designed for ancient human DNA called against the 1240K SNP list (Panel 2). We found no long runs of homozygosity in any of the populations, indicating that none were the result of recent consanguinity. The LoN, however, had more ROH than the other populations, suggesting a population bottleneck (or founder effect) and/or small population size (Fig. S7).

Further evidence for this emerged when we used hapROH to calculate the maximum-likelihood effective population size using a panmictic model (Table S2), with the LoN and KoS individuals yielding a small effective population size. We see small effective population size consistently in all LoN individuals when calculated separately, indicating that it is not the result of an unusual sample biasing results. The other published individuals all came from larger effective populations. The published Early Bronze Age individual from Lop Ness also showed a small effective population size, whilst in contrast during the Neolithic the effective population size was larger, although the Strath Glebe sample, from the Isle of Skye in western Scotland, is the lowest. Bell Beaker samples from France and the Netherlands have levels similar to Neolithic Orkney and mainland Scotland. Bronze Age sites in Britain and Ireland are higher still – almost an order of magnitude above those of Orkney. Thus, Orkney is unique in this dataset in witnessing a drastic reduction in effective size with the onset of the Bronze Age, likely due to founder effect during the arrival of new people after the Neolithic, subsequent relative isolation and small population size, and cultural practices such as endogamy.

As noted in the main text, a level of ~95% continental genome-wide ancestry could be achieved by the marrying out of indigenous men with immigrant women in only five generations, or 100–150 years. After one generation of introgression, the original genotype comprises about half the genome; after two, a quarter and so on. Five generations would amount to 100–150 years, depending on whether we assume generation time of 20 or 30 years.

S4 Modern mtDNA data processing

The Orkney Complex Disease Study (ORCADES) is a family-based, cross-sectional study that seeks to identify genetic factors influencing cardiovascular and other disease risk in the isolated archipelago

of the Orkney Isles in northern Scotland (103). Genetic diversity in this population is decreased compared to Mainland Scotland, consistent with the high levels of endogamy historically. 2078 participants, aged 16–100 years, were recruited between 2005 and 2011, most having three or four grandparents from Orkney, the remainder with two Orcadian grandparents. Fasting blood samples were collected and many health-related phenotypes and environmental exposures were measured in each individual. All participants gave written informed consent, and the study was approved by Research Ethics Committees in Orkney, Aberdeen (North of Scotland REC), and South East Scotland REC, NHS Lothian (reference: 12/SS/0151).

Similarly, the Viking Health Study – Shetland (VIKING) is a family-based, cross-sectional study that seeks to identify genetic factors influencing cardiovascular and other disease risk in the population isolate of the Shetland Isles in northern Scotland (104) Genetic diversity in this population is decreased compared to Mainland Scotland, consistent with the high levels of endogamy historically. 2105 participants were recruited between 2013 and 2015, most having at least three grandparents from Shetland. Fasting blood samples were collected and many health-related phenotypes and environmental exposures were measured in each individual. All participants gave informed consent, and the study was approved by the South East Scotland Research Ethics Committee, NHS Lothian (reference: 12/SS/0151).

We sequenced the 1356 ORCADES (Orkney) WGS samples from 500 ng of genomic DNA at the Wellcome Trust Sanger Institute using standard Illumina paired-end DNA library construction. We amplified adapter-ligated libraries using six cycles of PCR and subjected them to DNA sequencing using the HiSeqX platform (Illumina), according to manufacturer's instructions. We transformed base call files for each lane into unmapped BAM files using Illumina2BAM, marking adaptor contamination and decoding barcodes for removal into BAM tags. We mapped PhiX control reads using BWA Backtrack and used them to remove spatial artefacts. We converted reads to FASTQ files and aligned them to the GRCh38 reference genome within the bcbio workflow (1.1.5) (https://zenodo.org/record/3740787/export/json#.YIGf4j_TXIV), using BWA-MEM v.0.7.17 (39) to align them and post-processing them with GATK v3.8 (105). Sequences produced a mean depth of 20X. We sequenced the 500 VIKING (Shetland) WGS samples to a mean depth of 35X and aligned them against the GRCh38 reference genome at Edinburgh Genomics, University of Edinburgh; *see* Halachev *et al.* (106) for details. We used SAMtools v.1.3.1 (42) to separately extract reads aligned to chrM for both cohorts.

We processed 1356 BAM files from Orkney and 500 from Shetland. We sorted BAM files using SAMtools v.1.8 and removed PCR duplicates with DeDup, as described in the EAGER pipeline (107). We called the variants against the rCRS with GATK HaplotypeCaller v.3.8 (105). Within HaplotypeCaller, we set a minimum coverage at 2X, a standard call confidence at 30 and the ploidy at 100. Since the ploidy for the mitogenome is 1, setting it at 100 provided the percentage represented by each allele at each position and helped to filter heteroplasmies. We then filtered SNPs for a minimum quality of 30 using GATK v.3.8 (105).

For downstream phylogenetic analysis, we filtered the mutations by allele frequency. We considered allele frequencies greater or equal to 0.7 as true mutations, and frequencies between 0.69 and 0.3 as heteroplasmies. We considered differences from the rCRS reference sequence (40) below 0.3 as sequencing artefacts. We excluded unstable mutations denoted in PhyloTree, Build 17 (53). To classify sequences into haplogroups, we extracted variant lists from the consensus FASTA files and uploaded the variant list for each sample into HaploGrep 2 (52).

To build a maximum-parsimony tree (Dataset S2), we generated FASTA files from the list of SNPs for each sample and aligned our FASTA sequences against the rCRS with the software Sequencher® (version 5.1; Gene Codes Corporation, Ann Arbor, MI USA: http://www.genecodes.com). We imported

the sequences into mtPhyl (version 4.015), in order to estimate the maximum-parsimony phylogenetic tree (based on the HaploGrep2 classification) and exported it into an Excel sheet.

S5 mtDNA analyses

5.1 Phylogenetic and phylogeographic analysis of Orcadian lineages

We display Orcadian and Shetland mitogenome data, both modern (Dataset S1C) and prehistoric (Dataset S1G), as a maximum-parsimony tree in Dataset S2. We used published data for further comparative analyses (11, 12, 47, 68, 71, 83, 87, 88, 91, 101, 108, 109).

Early Neolithic Orkney (*n* = 21) (12, 47) includes lineages belonging to HV0a – classified as V in Olalde *et al.* (12) – H1, H3, several H5, several in J1c1b, J1c2, J1c9, T2c1d1, U8b1b, three K1a+195, K1a4, K1a1b1, two K1b1a1, U5b1 and U5a2c3. The high levels of K1a, H5, J1c and T2c1d are characteristic of the European Neolithic generally, U8b1b points to the central European Neolithic (62, 83, 87), and H1, H3, K1a1b1 and K1a4 point to the Mediterranean/Rhône/Atlantic Neolithic (110, 111), overall suggesting mixed ancestry tracing the settlement ultimately from both major Neolithic dispersal routes, but predominantly from the western Neolithic.

The Bronze Age Links of Noltland (*n* = 20), includes a number of minor H lineages, including H39 (represented in five samples in total, although the READ results indicated that two samples were from a single female individual), H58a, H+195, and two individuals with H1n1; two with J1c2a; three with T2a1b1a (matching one individual from Lop Ness, further south in Orkney, the only previously published CA/BA Orkney sample (12); two with T2b21; two with U5b2a3; and one each of K1a3a, K1a29a and K1c2. Eight of these individuals were part of a multiple burial and thus potentially closely related (although we could only confirm one familial relationship: see 3.6): three of the four H39 individuals, all three T2a1b1a individuals, one of the two U5b2a3 individuals, and the K1a3a individual. The males from the multiple burial all carried Y-chromosome haplogroup I2a1b-M423. The Iron Age sample from Knowe of Skea, on the southern coast of Westray (*n* = 3), includes two identical H1b lineages and U5a1b1a.

Whilst the Iron Age samples include lineages that might be associated with Beaker immigration, deriving ultimately from the Corded Ware or Yamnaya cultures, this is less obvious for the Bronze Age, and many diagnostic Corded Ware/Yamnaya lineages, such as H6a, H13a, U5a and T1a, are absent. Nevertheless, there are difficulties with attributing a source to these lineages since the likely alternatives are potentially very similar. Beaker Britain was most likely settled from the Low Countries (12), which themselves will have been carrying lineages introduced from both the LBK and the Mediterranean Neolithic expansions, as well as lineages brought later on from east-central and eastern Europe with the Corded Ware and Yamnaya. It is likely that the great majority of the mtDNAs in Bronze Age Orkney arrived with Beaker or Bronze Age immigrants.

T2a1b1a is seen not only in three LoN individuals, but also a further Bronze Age Orcadian from Lop Ness and two more from Bronze Age Scotland. T2a1 has some clear Steppe ancestry, and T2a1b1* is seen in the Corded Ware of Germany, pointing to a Corded Ware ancestry. T2b21 matches German and Czech Beaker lineages and so Beaker immigration is most likely. Neither T2b21 nor T2a1b1a are seen in modern Orkney or Shetland, although there is a single T2a1a in Orkney and numerous T2b lineages in both.

There are four apparent H39 lineages (with just the variant at position 16299 in common). The northern modern distribution (restricted to Scandinavia, Germany and the British Isles), aged to ~5 ka, suggests that these most likely spread with the Corded Ware from the east. K1c2 is similar: it is also rare and not otherwise seen in prehistoric individuals, but its modern northern European distribution and age (~4.6 ka) again suggest dispersal from the north-east. It is seen in multiple individuals in modern Orkney and Shetland.

Several more lineages are potentially Beaker immigrants, although the situation is less clear. J1c2* is present in Neolithic Orkney, but even so it is also widespread in (modern) southern and central Europe and could, therefore, have been re-introduced as J1c2a with the Beaker complex. K1a3a is analogous – K1a3* is present in both Neolithic and Chalcolithic Scotland, but K1a3 most likely arose in the Late Glacial in the Near East (112), so it dispersed separately during the Neolithic and may have arrived independently in Orkney in the Beaker period.

H1n1 is absent from other prehistoric samples and is distributed across north-central Europe in modern samples. It dates to ~7 ka, and H1n has deeper roots in Mediterranean Europe with a more recent ancestry in the northeast. It could have entered Britain either in the Neolithic or the Beaker age. H58a is similarly absent from other prehistoric samples, and is restricted to modern Spain, Denmark and Britain. It dates to ~6-7 ka, which may suggest it reached Orkney with the Neolithic and was inherited into the Bronze Age, rather than arriving later, but there is no clear evidence either way. H+195, whilst also seen in one Middle Neolithic Hungarian individual, has a very similar modern distribution and is similarly enigmatic.

U5a1b1, seen in an Iron Age Knowe of Skea individual, is also seen in continental Beaker lineages and most likely arrived with the Beaker complex. Although the Knowe of Skea U5a1b1a lineage does not match any modern lineages from the Northern Isles, U5a1b1e is common in Orkney and U5a1b1g is seen in both a Scottish CA/EBA lineage and in modern Shetland.

There are also two H1b* individuals in Knowe of Skea. Although the age and modern distribution suggests that H1b spread with the western Neolithic, the oldest prehistoric sample is from the Globular Amphora Neolithic from Poland, in the region preceding the spread of the Corded Ware, with others (in H1b1) comprising Beaker/Bronze Age samples from central Europe. Therefore, these lineages may have been assimilated by the Corded Ware and Beaker complexes, raising the possibility that the H1b in Iron Age Orkney may also have arrived with the Beaker complex. H1b1, although not H1b*, is present in both modern Orkney and Shetland.

As discussed in the main text, the lineage most likely to date to before the Beaker Age in Orkney is U5b2a3+16319, which we have named U5b2a3b, seen in two LoN individuals. We estimated the age of U5b2a3 and U5b2a3b through an aDNA calibration in BEAST v.2.1 using an uncorrelated lognormal relaxed clock, the HKY85 model of nucleotide substitutions and gamma-distributed rates (113). For the analysis we used seven dated ancient mitogenomes for the calibration (UNTA85_113, scy301, I2988, BERG746, I3033, PB754 and KD062) (11, 12, 101, 110, 114, 115) and 85 published modern U5b2 sequences, including five U5b2a3 sequences (EF420249, EU682506, JQ702815, JX153147 and NA20517). The British subclade U5b2a3b dated to the Early Neolithic (6315 [3720; 9960] ka); U5b2a3 itself dated to the Mesolithic (10,500 [7005;14660] ka) and is seen in Early Neolithic individuals from the Parknabinnia court tomb in western Ireland (101), western Scotland (Arran) (12) and west Wales (11), as well as in a Middle Neolithic individual from eastern France (110) (Dataset S3 – note that the long branch to the Welsh sample points to possible issues with aDNA data quality). A subclade of U5b2a3 with a variant at position 16240 is seen in a Beaker individual from Bavaria in southern Germany (115) and an Iron Age Scythian from Moldova (114) but otherwise the lineage seems largely restricted to northwest Europe and, with U5b2a present in the Mesolithic of Ireland as well as Neolithic Britain, U5b2a3b (and some other U5b2 lineages) might potentially indicate continuity from the Mesolithic.

U5b2a3b is also seen in one modern individual from the British Isles (116), and one individual from Virginia in the United States (an early British-founded colony) suggesting potential continuity through to the present day. Although Mesolithic samples from England and Wales to date belong to U5b*, U5b1 or U5a2 (11), U5b2a* is seen in Neolithic Orkney (47), as well as Neolithic Oban in western Scotland (12). U5b2b is also seen in modern Orkney and Shetland, as well as in Chalcolithic/EBA southeast and northeast England (12) and U5b2c is also seen in modern Orkney and Shetland (Dataset S2), as well as Neolithic Primrose Grange in Ireland (47).

5.2 Evidence for continuity from Neolithic to Beaker/Bronze Age Britain overall, from main database matching variant haplotypes

Out of 104 British Beaker/CA/BA samples (12), 21 mtDNAs (20%) show direct matches with British Neolithic lineages, whereas only 3/53 (5.6%) show autochthonous (I2a2a vs. R1b1a1a2-M269) Y-chromosome lineages. This suggests an overall Bronze Age male sex bias on the British mainland, with more assimilation of local Neolithic lineages on the female than the male side, as was also the case for continental Europe, Iberia and India (91, 117, 118) – and the reverse of the situation in Orkney.

Details:

- Four identical H1, two in Neolithic Orkney, two in Beaker and MBA southern England
- Two H1+16189, Low Hauxley Beaker/BA and Neolithic Orkney
- Three identical H1c, from England CA/EBA, English Neolithic and Scottish Neolithic
- Four identical H5, from Beaker Britain, Neolithic Orkney (two) and Neolithic Wales
- Two identical HV0+195, from Beaker southern England and Neolithic Scotland
- Three identical J1c1, from CA/EBA and MBA southern England and Neolithic Scotland
- Two identical J1c2, from MBA southern England and Neolithic Orkney
- Two identical K1a4a1, Scotland Neolithic, Beaker southern England
- Six identical K1b1a1, two Orkney Neolithic, four southern England CA/EBA
- Six identical T2b, one Neolithic southern England, four Beaker/CA/EBA England, one MBA England
- Three identical X2b+226, two in England Neolithic, one England CA/EBA

S6 Y-chromosome analyses

6.1 Methodology

Y-chromosome data are presented in Dataset S1H. For paternal haplogroup prediction, we used pathPhynder (119) and we developed the software with an additional script that both makes explicit the uncertainty associated with the assignment of each a sample to a particular node in the Y-chromosome tree (by visualising the completeness or incompleteness of the SNP evidence) and also increases the resolution of the sample placement in the tree for haplogroups defined by SNPs absent from the reference dataset.

The degraded nature of ancient DNA makes phylogenetic and phylogeographic analysis challenging, as missing diagnostic SNPs can hamper the building of reliable phylogenetic trees. One solution, proposed by the software pathPhynder, maps the SNPs of ancient samples onto a robust phylogenetic tree built from modern data in order to estimate the most likely phylogenetic position of each ancient sample.

We first used pathPhynder to assign the mutations of each branch of the Y-chromosome tree from the 1000 Genomes Project (120). Secondly, pathPhynder generated a SNP list and ancestral states of each ancient sample classified into haplogroups according to the IOGG 2018 (121) nomenclature. We used pathPhynder's default parameters which filter C to T and G to A mutations covered by only one read to reduce the effect of post-mortem deamination on the downstream analysis. Next, pathPhynder traversed the 1000 Genomes Y-chromosome tree to count the number ancient SNPs that overlap with the modern variants and which support or are in conflict with membership to each one of the tree branches, in order to identify the most likely position of the ancient samples in the phylogeny.

Using the pathPhynder result, we then developed a set of Python scripts for a more detailed analysis. We first added the haplogroup classification obtained by pathPhynder for both modern and ancient samples onto the tree. Secondly, we annotated the tree by the archaeological period for the ancient samples and by the geographical origin for both ancient and modern samples.

Thirdly, we annotated the nodes of the tree by identifying the position of each haplogroup. To do this, using the YFull (v9.01.00: https://www.yfull.com/tree/) (122) and the ISOGG Y-chromosome 2018 tree torso (https://isogg.org/tree/2018/index18.html), we built a guide tree that classifies lineages from the root to the terminal haplogroups into each Y-chromosome haplogroup. Thus, by looking at the phylogenetic position of each sample and its haplogroup, the script searches automatically for their common ancestor (nodes) and identifies in the guide tree the corresponding haplogroups to annotate all the nodes of the tree (Fig. S10).

According to the newly annotated tree (labelled for haplogroups), we also added onto each branch the mutations of the ancient samples that overlapped with the modern mutations. However, pathPhynder only takes into account the variants which are shared between each ancient sample and the modern samples in order to place the ancient samples on the tree. To improve the resolution, we also wished to carry out a pairwise comparison between different ancient samples, to ensure that any mutations in the ancient samples that are absent in the modern reference dataset are also taken into account. Thus, if these new informative SNPs lead to a new derived branch or branches, not present in the modern tree, the script creates the new branch(es), annotates the nodes with the haplogroup information, the branch(es) with the respective haplogroup defining mutations, and adds the ancient samples to their new phylogenetic position in the tree (Fig. S11). For example, in Fig. S15**,** all the sub-branches derived from the branch I2a1a2a have been identified by pairwise comparison between ancient samples, because these diagnostic mutations are not described in the modern samples.

To ensure that the phylogenetic position/haplogroup classification of each ancient sample is reliable, we have also taken into account the ancestral states and missing SNPs. From the haplogroup classification of each ancient sample, the script searches the ISOGG (2018) nomenclature of the diagnostic SNPs for their first derived sub-haplogroups. It then searches the pileup file generated by pathPhynder, if these diagnostic SNPs are in an ancestral state as defined by pathPhynder, or if they are missing (gap). Following this approach, we identified three classifications. The first classification ("[*]") is when all the first derived sub-haplogroups, as described by ISOGG, have at least one diagnostic SNP defined as in an ancestral state by pathPhynder. The ancestral state for all derived sub-haplogroups reinforces the haplogroup classification by confirming that we cannot expect a deeper haplogroup classification because of missing data (Fig. S12A).

The second classification ("[x?/x2]") can be described when one of the two next derived haplogroups have missing data for all their diagnostic SNPs. In this case, we cannot exclude a more downstream haplogroup classification given that the ancient sample only has missing data at the relevant SNPs. However, the ancestral state for the second derived haplogroups confirms that we cannot have a more suitable haplogroup classification inside that subclade (Fig. S12B).

The final classification ("[?]") is when we have missing data for all the next derived haplogroups. Here, we cannot confirm the haplogroup classification with the existing data, meaning that a more refined classification would likely be possible with improved coverage (Fig. S12C).

We generated the final tree with the Python library ete3 (123). The phylogeography of the I2a and R1b-M269 subclades of the whole tree is shown in Figs. S13 and S14, and the detailed trees are shown in Figs. S15 and S16.

Note that interpreting Y-chromosome haplogroup nomenclature requires considerable caution. The widely accepted nomenclature is that provided by ISOGG. However, as is also the case for mtDNA, the Y-chromosome phylogeny is continuously being refined and updated as new sequences are added (*see* the YFull tree for an up-to-date Y-chromosome tree with dated nodes). Unfortunately, when ISOGG revises the nomenclature every year it is often not conservative with respect to the revision of nomenclature. This can create great confusion, as the names of clades are subtly (or even quite

substantially) reorganised – so that, for example, a clade labelled in 2018 can share a name with a completely different clade labelled in 2016 or 2017.

For the main text of this paper, we have more conservatively followed the consensus amongst recent aDNA papers of using the 2017 ISOGG classification, allowing cross-comparison with their data tables, but we note that not all authors have followed this convention. An important exception in the present context is Cassidy *et al.* (101) who followed the 2019 classification. We provide a brief translation guide for the lineages encountered here in Table S3.

6.2 Haplogroup R1b-M269 variation

As described in the main text, all but one of the nine males sampled at LoN carried variants of Y-chromosome haplogroup I2a1b-M423. Just one individual at LoN, an infant who was not from the multiple burial, carries R1b-S145, a derived form of the Y-DNA lineage R1b-M269, which arrived in Britain with the Bell Beaker Complex (12). R1b-M269 is the main male lineage that dispersed from the Pontic-Caspian Steppe after around 5000 years ago (68, 109). R1b-S145 is found today predominantly in western and northern Britain, Ireland, Brittany and Iceland, with a lower presence in northern Iberia and the Low Countries (124), and has been seen in Bell Beaker, BA, IA and Romano-British aDNA genomes (125) (Figs. S13, S15).

R1b-S145 has not been found in prehistoric Iberian males (n = 171); it appears for the first time there in Roman times, between the third and sixth century AD (91). Its immediate ancestor, R1b-S116, is, however, seen in prehistoric Iberian samples dating back as far as ~2500–2000 BC and in much more substantial numbers from the onset of the BA (almost 40% of 39 BA samples) (91). R1b-S116 is seen earliest in Beaker Complex remains from western/central Europe, with radiocarbon dates upwards of 2200 BC in the Netherlands, France, England, Hungary and Germany (12). Given a radiocarbon date of 2469–2296 cal. BC from Dorset, on the south coast of England, for R1b-S145 (12), these data together suggest an arrival in Britain of R1b-S116 and a rapid formation of R1b-S145 within Britain around 2500–2300 BC.

The LoN infant (KD061) is further derived at CTS241 and CTS8221 (indicating haplogroup R1b-DF13), which dates in the modern Y tree (YFull) to ~4000 years old. This lineage has also been seen in the EBA, ~2000–1500 BC at Rathlin Island, on the Northern Ireland coast close to Scotland, alongside Beaker-like food vessel ceramics (75), and from Beaker Complex England and CA/EBA and MBA Scotland and England from ~2400 BC onwards.

The two male KoS IA samples also belong to the R1b-M269 lineage, with one of them also carrying S145 and the derived DF13 marker, like the BA LoN individual. Although the IA samples are few, this result might suggest that R1b-M269 lineages were becoming more prominent in Westray by around 2000 years ago. The R1b-DF13 subclade is the most frequent Y-chromosome lineage in western Britain and Ireland today; its predecessor R1b-S116 is seen in 9/20 present-day Orcadians (126), where medieval Viking settlement shifted Y-chromosome frequencies again (127), and its immediate its immediate predecessor R1b-S145 is found at 49–67% in Scotland, 48–94% in Ireland, 45–56% in Wales and 38–40% in western England, although only 12–16% in eastern and central England due to later arrivals, such as Anglo-Saxons (124, 128, 129).

The Beaker burial from Low Hauxley, in Northumberland, also belongs to R1b-M269, and the Pictish sample from Rosemarkie belongs to R1b-S129, supporting the general picture of the ubiquity of derived forms of the R1b-M269 lineage in post-Neolithic Britain (12), which stands in sharp contrast to the high frequency of I2a1b lineages identified in the Orkney Bronze Age.

6.3 Sex bias, patrilocality, exogamy and dispersal

The discovery that pastoralists from the Pontic-Caspian Steppe dispersed into Europe ~3000–2500 BC, assimilating the indigenous Neolithic populations, and later also across Central and South Asia in the Middle/Late Bronze Age – whilst most likely also spreading Indo-European languages (12, 68, 75, 85,

90, 109, 130) – is one of the most important developments in the study of prehistory of the past few years. In many regions, this process was accompanied by a significant sex bias: there is a preponderance of incoming male lineages and indigenous female lineages (84, 91, 117, 118, 131). For example, in a large Beaker Age dataset from Britain (12), ~20% of mtDNA haplotypes directly match British Neolithic mtDNA lineages, whereas little more than 5% of Y-DNA lineages are autochthonous (*see* S5).

It has been suggested that this early sex bias was a product of a patriarchal society organised via roaming male war bands (117, 132, 133), which may also have been adopted by Late Neolithic societies in north-central Europe in response to the arrival of aggressive Corded Ware groups (134). However, it is clear from archaeological evidence that women were often high-status members of these communities, both amongst the early Yamnaya and during the BA (135, 136). The underlying common factor may rather have been patrilocality and exogamy – the cultural imperative for men to marry women from outside the immediate community (115, 137, 138), such as seen in the Omaha kinship system inferred by historical linguists for Proto-Indo-European speakers (139).

There have been several attempts to use aDNA alongside stable isotope analyses to examine local and regional datasets and cast light on prehistoric social structures, following a pioneering study (before the advent of NGS) of early Corded Ware burials dating to ~4.6 ka in Germany by Haak *et al.* (137). This work identified several nuclear family burials and indicated that whilst the men and children were probably local, the women were from further away, pointing to patrilocality and exogamy. Similar conclusions have been reached using more powerful NGS techniques: for example, other studies (115, 138) have examined Corded Ware, Bell Beaker and BA variation in southern Germany. By combining genome-wide analysis with strontium and oxygen isotope data, they proposed that high-status patrilocal family households were emerging in the BA living alongside both unrelated low-status individuals from the same localities and unrelated high-status females from further afield. At the same time, however, a process of incoming pastoralists assimilating local Late Neolithic farming populations was also unfolding. These authors cautioned that part of what archaeologists identify as “migration” might rather be accounted for as institutional sex-specific mobility.

In fact, patrilocality has previously even been suggested for Early Neolithic groups dispersing into central and western Europe as a possible explanation for reduced diversity amongst Y-DNA compared to mtDNA lineages (140, 141). Although the variance in male *versus* female offspring is a confounding factor, it has also been suggested on the basis of strontium isotope patterns (142), possibly as a result of a more sedentary life-style (143), albeit without sex bias in the dispersal patterns (118).

However, the situation regarding Yamnaya and Corded Ware patrilocality and female exogamy is complicated by the fact that it does indeed seem to be overlaid onto large-scale migration, which itself seems to have been sex biased. Although differing in many important ways, both direct Yamnaya migrants and the bearers of the Corded Ware culture carried a substantial genome-wide component from the Steppe region, with the former especially contributing to the formation of the Bell Beaker Complex across central and western Europe after 2500 BC. These dispersing groups also carried distinct suites of Y-DNA lineages that originated amongst the hunter-gatherer populations of the western Steppe – mainly R1a1a-M17 lineages amongst Corded Ware and BA Central Asian groups, and R1b-M269 lineages amongst the European Yamnaya and Bell Beaker groups.

A paradoxical consequence emerges from the suggestion that populations were both dispersing west and east from the Steppe and at the same time likely to have been practising patrilocal residence and thus drawing in women from beyond their culture. In practice, the expanding populations seem to have been male-driven, assimilating female lineages from the areas into which they were moving and, therefore, becoming gradually more like the populations of the regions they were settling genetically, both over time and as they moved further from the source. Both dispersal and patrilocality were operating to transform the gene pool.

The specifics of this process varied from place to place. In Iberia, for example, at the south-western extreme of Europe, with a strong initial sex bias, there was a rapid and very heavy replacement of male lineages during the BA, but a more gradual transformation of other parts of the genome as a result of continual recurrent gene flow over the millennia (91, 93). Within Britain, on the other hand, there seems to have been a much more substantial replacement of the genomes of the whole population (12).

Supplementary Figures S1 to S16 and Tables S1 to S3

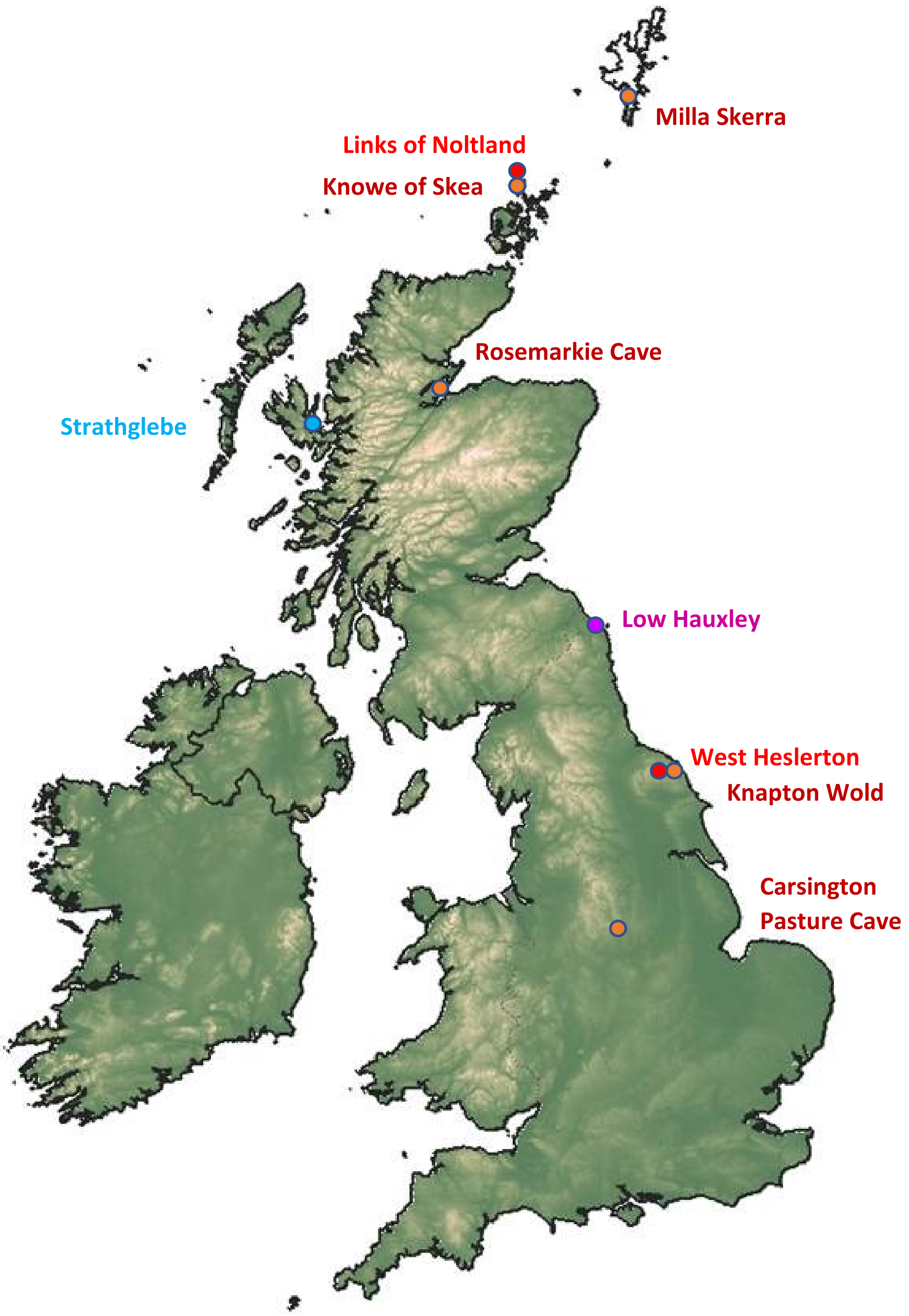

Fig. S1. Sample locations in Britain. Blue = Neolithic, pink = Beaker, red = Bronze Age, brown = Iron Age**.** Source for base map: https://srtm.csi.cgiar.org

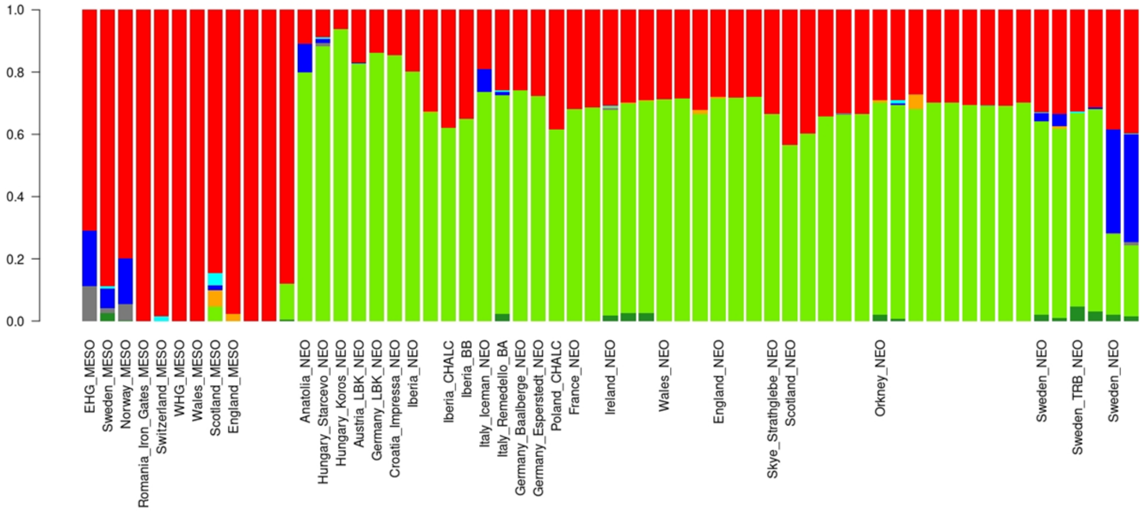

Fig. S2A. Unsupervised ADMIXTURE PLOT (*K*=7, expanded dataset) of European Mesolithic and Neolithic samples. The red component maximises in the WHG, green in the ANF, and blue in the CHG. Unlabeled profiles to the right of each label are from the same population. Note that very minor component (pale blue, yellow, grey etc.), which are mainly maximised in other modern non-Eurasian populations, are unlikely to be meaningful but rather due to noise in lower-coverage samples.

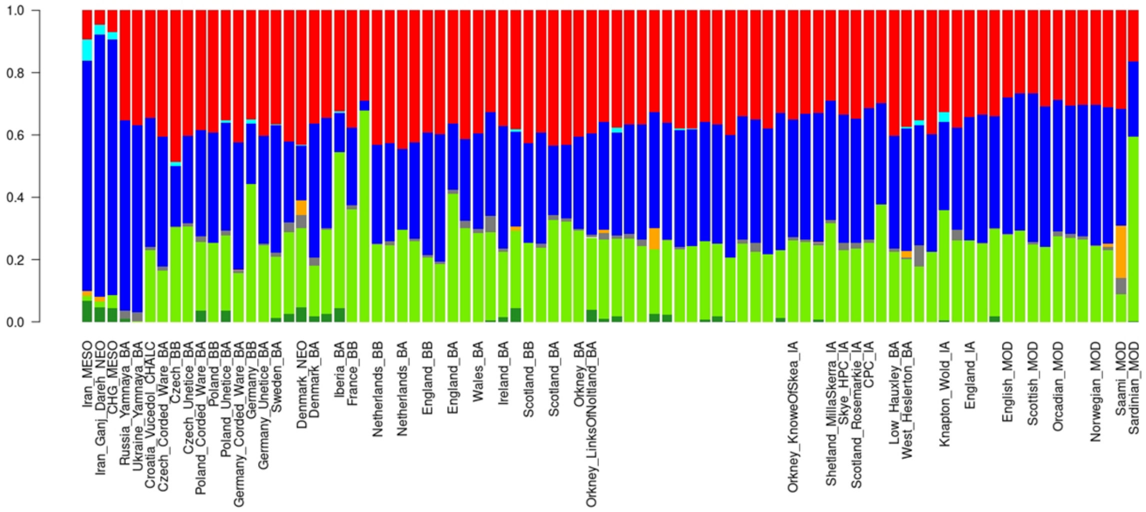

Fig. S2B. Unsupervised ADMIXTURE PLOT (*K*=7, expanded dataset) of Iranian Mesolithic/Neolithic and Bronze Age, Iron Age and modern (MOD) samples. The red component maximises in the WHG, green in the ANF, and blue in the CHG. Unlabeled profiles to the right of each label are from the same population. See the caveat mentioned in legend to Fig. S2A.

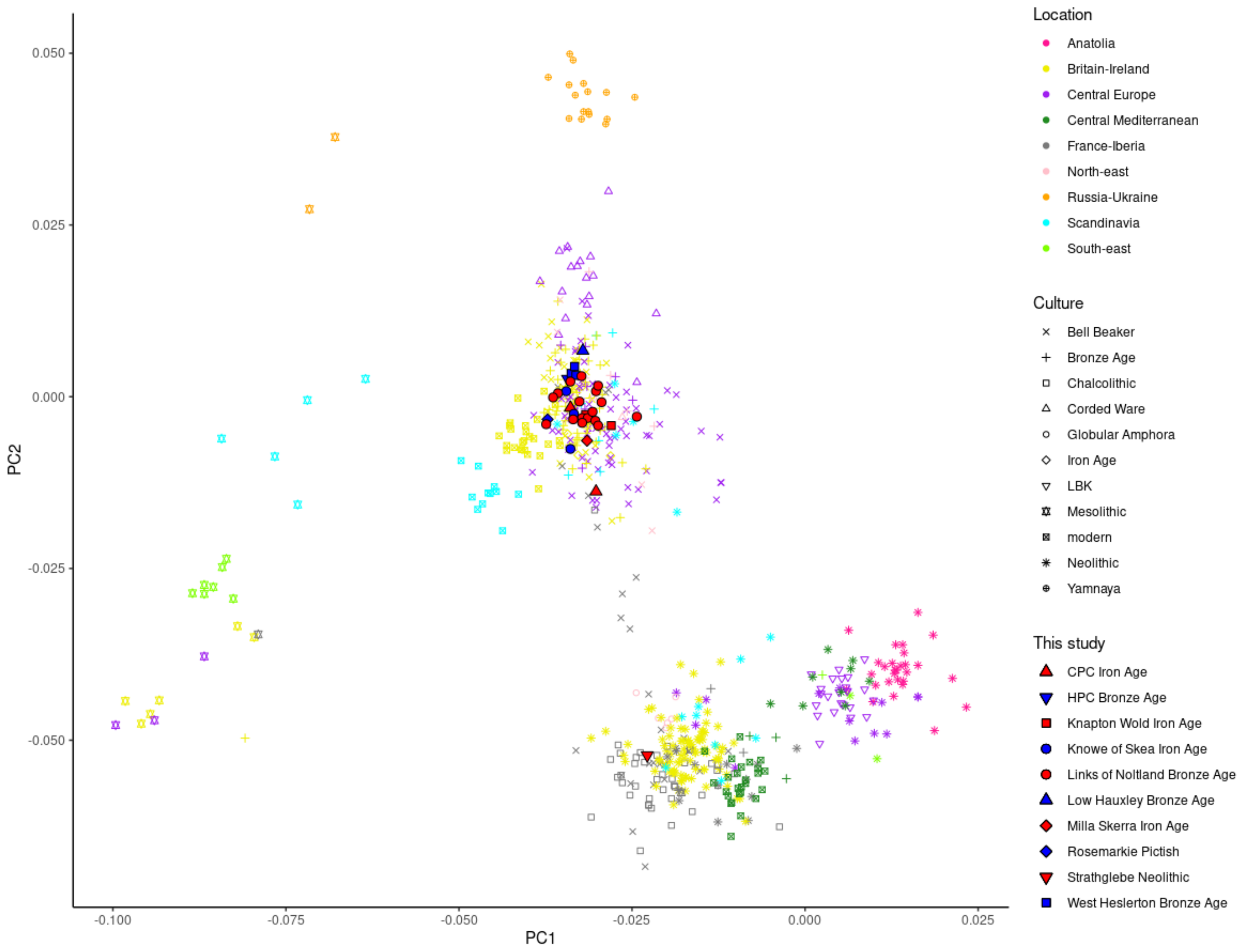

Fig. S3. Full PCA showing first two principal components (amounting to 0.8% and 0.4% of the variance respectively) of European Mesolithic, Neolithic and Bronze Age samples projected on present-day European variation.

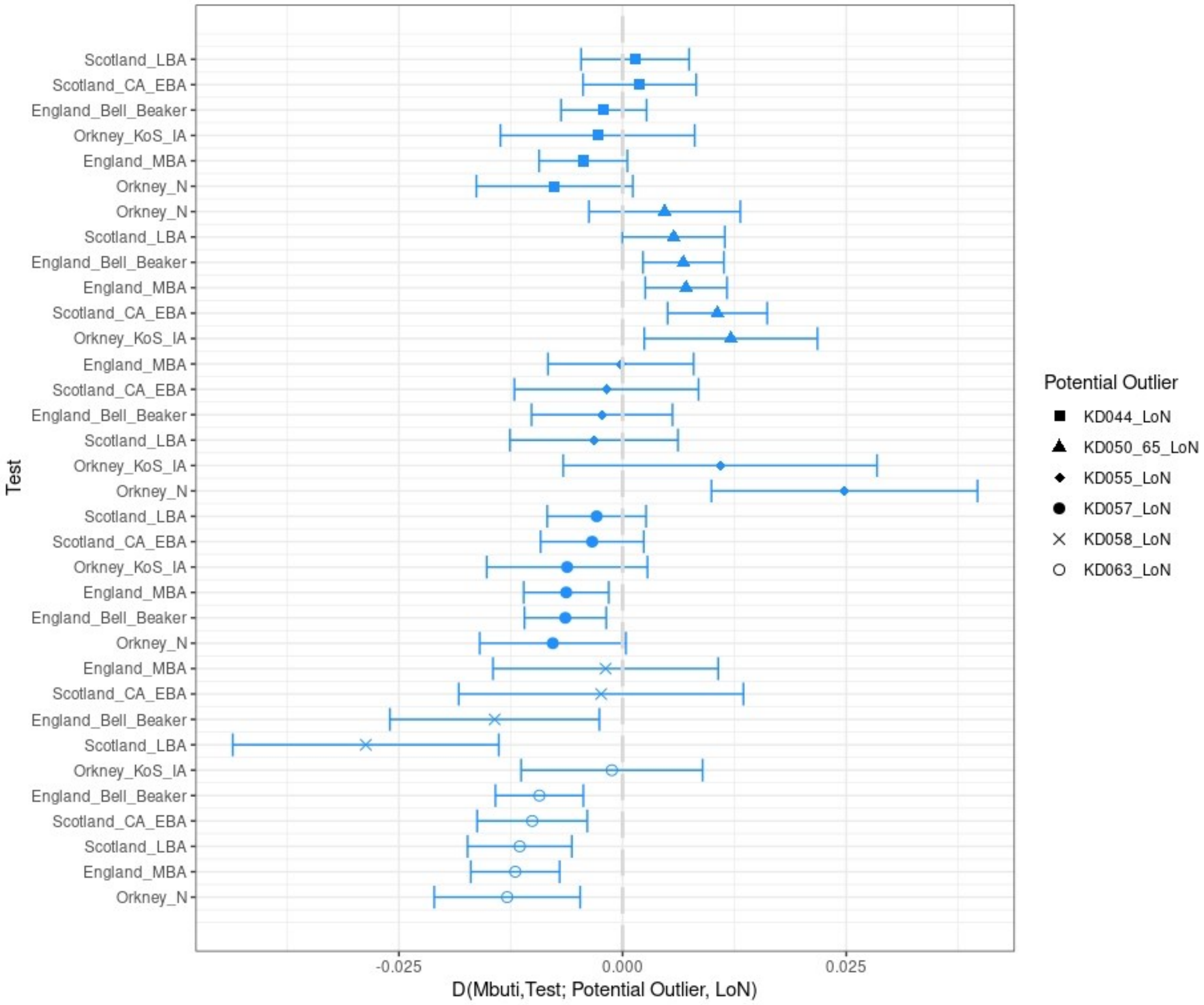

Fig. S4A. Symmetry *D*-statistics for Links of Noltland individuals. Results are all insignificant, $|Z|<3$, demonstrating that the Links of Noltland individuals form a clade.

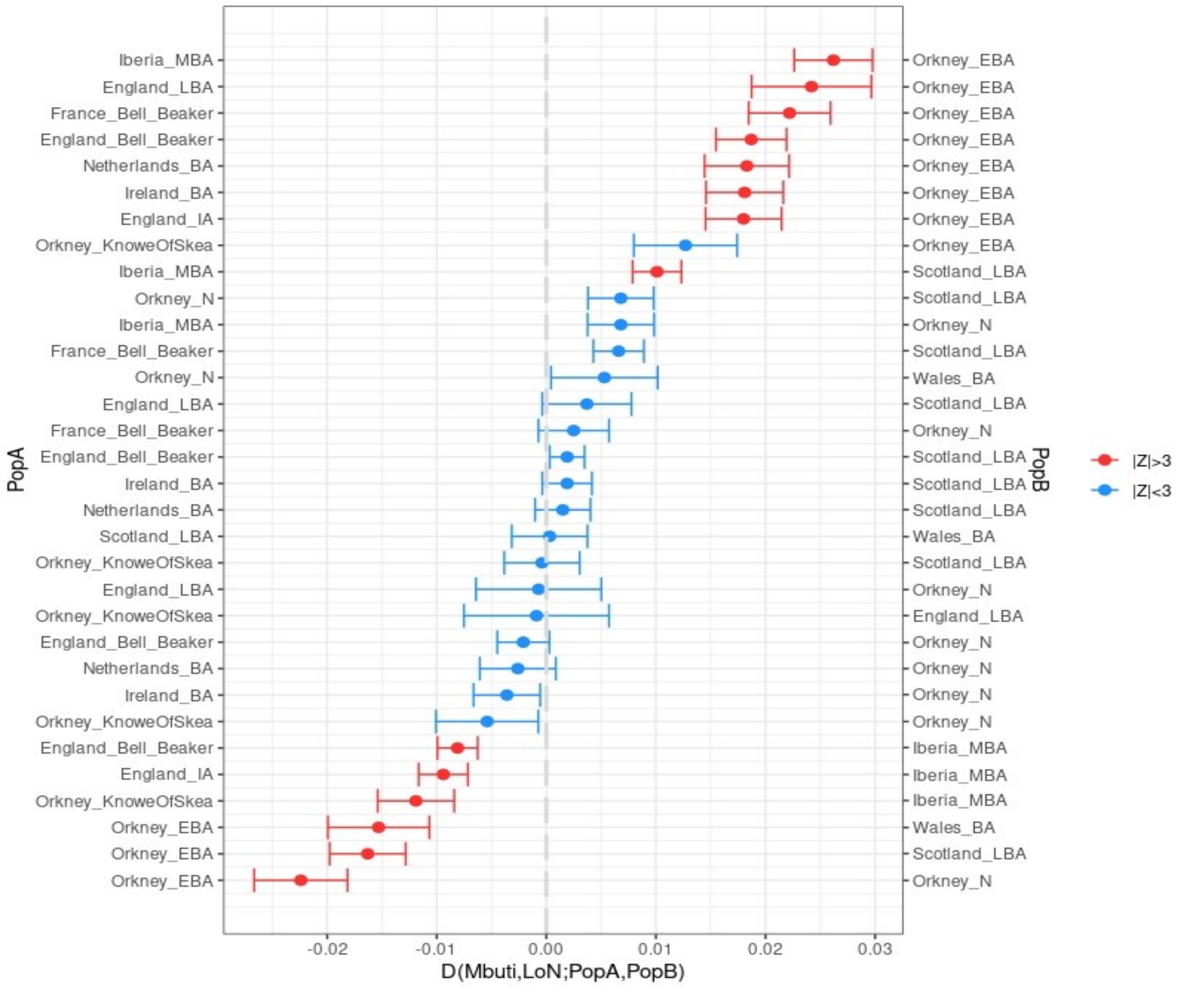

Fig. S4B. *D*-statistics (significant results, |*Z*|>3, are in red) for Orkney Links of Noltland genomes.

A

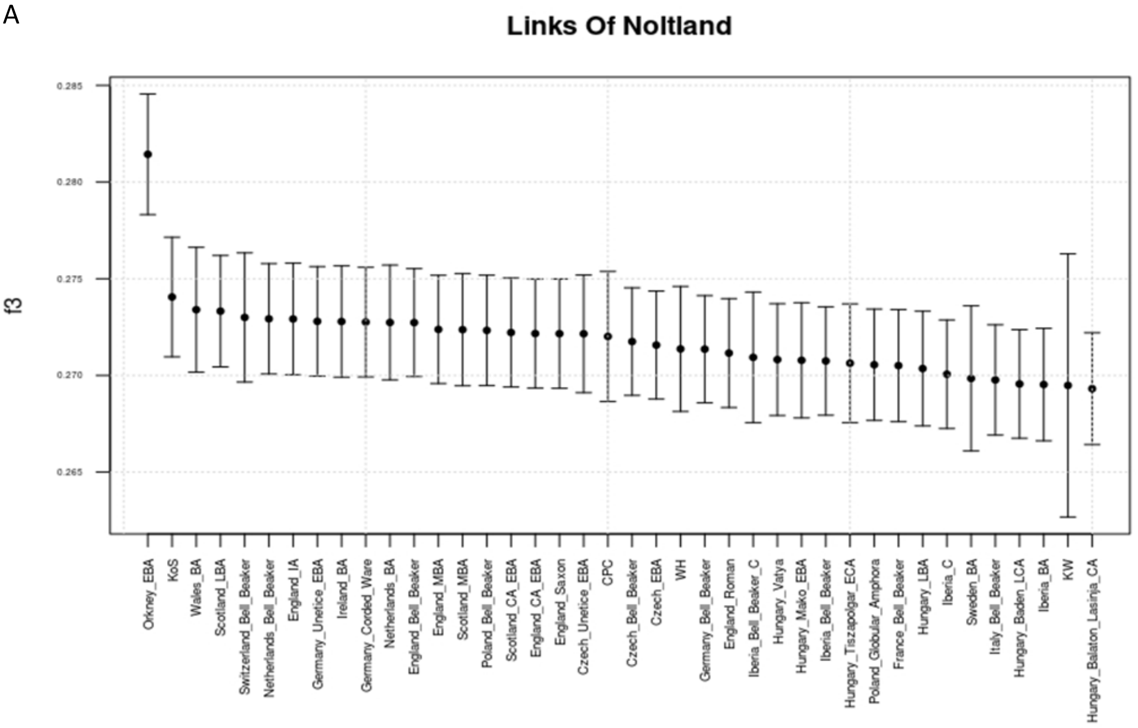
Links Of Noltland
0.285
0.280
0.275
0.270
0.265
f3
Orkney_EBA
KoS
Wales_BA
Scotland_LBA
Switzerland_Bell_Beaker
Netherlands_Bell_Beaker
England_IA
Germany_Unetice_EBA
Ireland_BA
Germany_Corded_Ware
Netherlands_BA
England_Bell_Beaker
England_MBA
Scotland_MBA
Poland_Bell_Beaker
Scotland_CA_EBA
England_CA_EBA
England_Saxon
Czech_Unetice_EBA
CPC
Czech_Bell_Beaker
Czech_EBA
WH
Germany_Bell_Beaker
England_Roman
Iberia_Bell_Beaker_C
Hungary_Vatya
Hungary_Mako_EBA
Iberia_Bell_Beaker
Hungary_Tiszapolgar_ECA
Poland_Globular_Amphora
France_Bell_Beaker
Hungary_LBA
Iberia_C
Sweden_BA
Italy_Bell_Beaker
Hungary_Baden_LCA
Iberia_BA
KW
Hungary_Balaton_Lasinja_CA

B

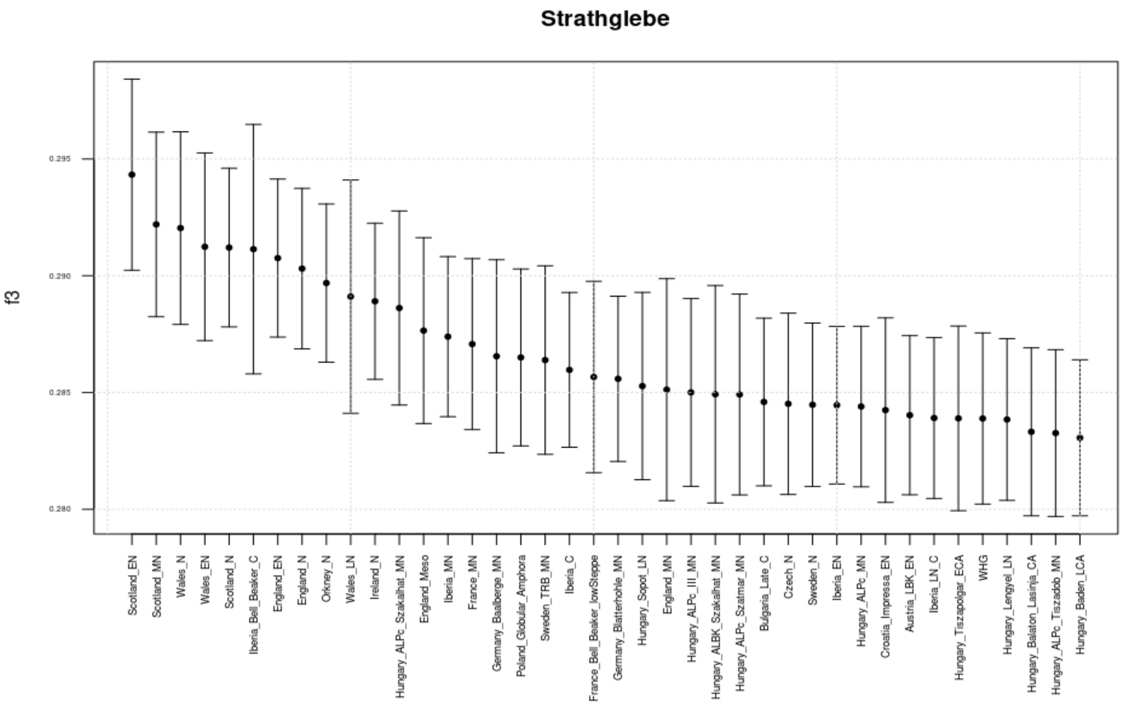
Strathglebe
0.295
0.290
0.285
0.280
f3
Scotland_EN
Scotland_MN
Wales_N
Wales_EN
Scotland_N
Iberia_Bell_Beaker_C
England_EN
England_N
Orkney_N
Wales_LN
Ireland_N
Hungary_ALPc_Szakalhat_MN
England_Meso
Iberia_MN
France_MN
Germany_Baalberge_MN
Poland_Globular_Amphora
Sweden_TRB_MN
Iberia_C
France_Bell_Beaker_lowSteppe
Germany_Blatterhohle_MN
Hungary_Sopot_LN
England_MN
Hungary_ALPc_III_MN
Hungary_ALBK_Szakalhat_MN
Hungary_ALPc_Szatmar_MN
Bulgaria_Late_C
Czech_N
Sweden_N
Iberia_EN
Hungary_ALPc_MN
Croatia_Impressa_EN
Austria_LBK_EN
Iberia_LN_C
Hungary_Tiszapolgar_ECA
WHG
Hungary_Lengyel_LN
Hungary_Balaton_Lasinja_CA
Hungary_ALPc_Tiszadob_MN
Hungary_Baden_LCA

C

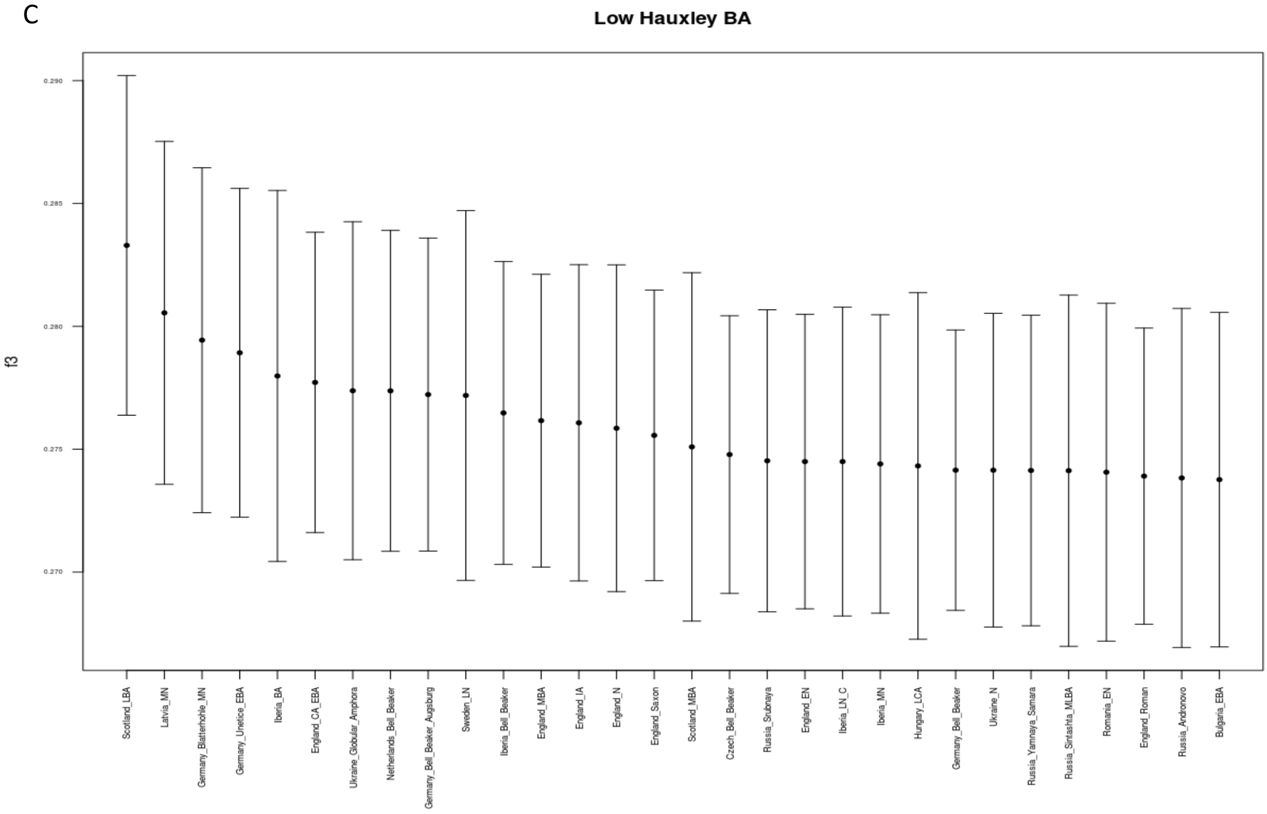
Low Hauxley BA
f3
0.290
0.285
0.280
0.275
0.270
Scotland_LBA
Latvia_MN
Germany_Blatterhohle_MN
Germany_Unetice_EBA
Iberia_BA
England_CA_EBA
Ukraine_Globular_Amphora
Netherlands_Bell_Beaker
Germany_Bell_Beaker_Augsburg
Sweden_LN
Iberia_Bell_Beaker
England_MBA
England_IA
England_N
England_Saxon
Scotland_MBA
Czech_Bell_Beaker
Russia_Srubnaya
England_EN
Iberia_LN_C
Iberia_MN
Hungary_LCA
Germany_Bell_Beaker
Ukraine_N
Russia_Yamnaya_Samara
Russia_Sintashta_MLBA
Romania_EN
England_Roman
Russia_Andronovo
Bulgaria_EBA

D

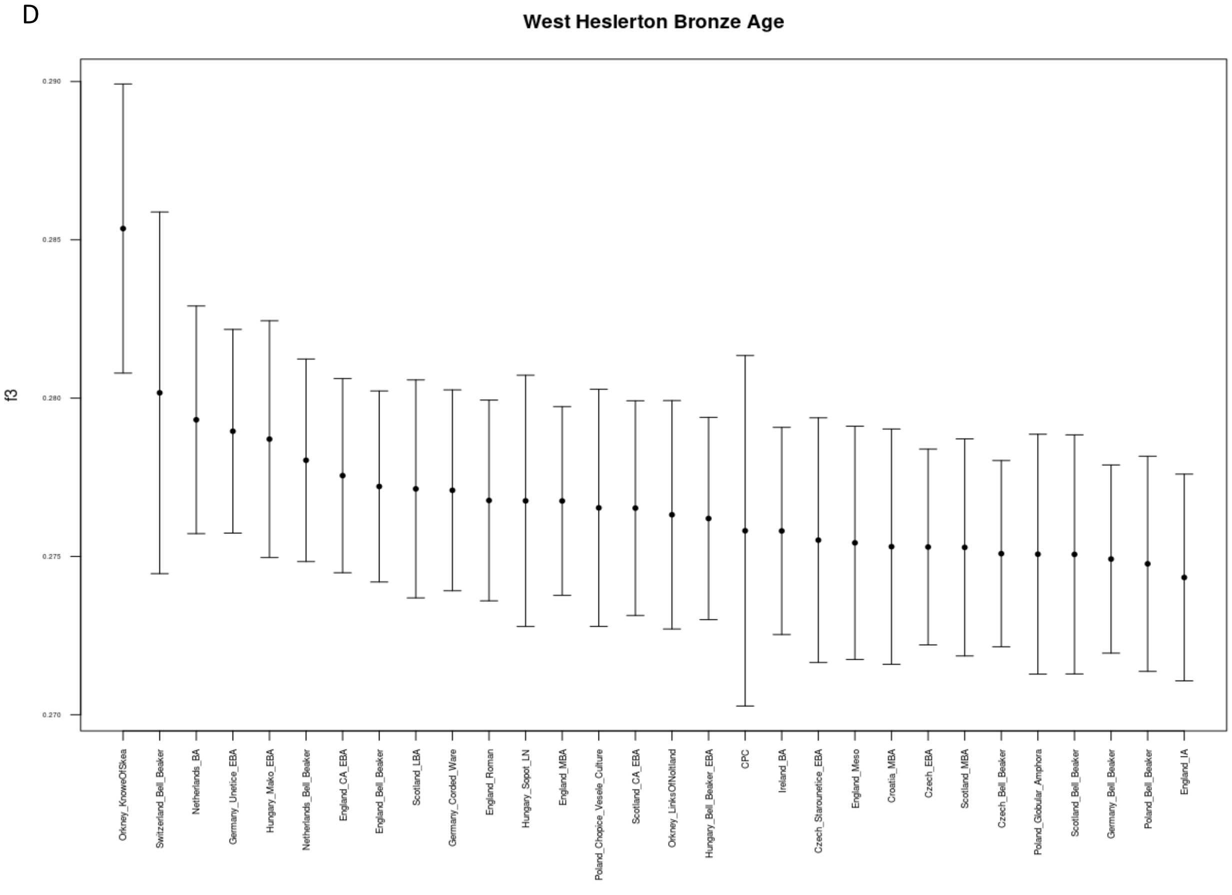
West Heslerton Bronze Age
f3
0.290
0.285
0.280
0.275
0.270
Orkney_KnoweOfSkea
Switzerland_Bell_Beaker
Netherlands_BA
Germany_Unetice_EBA
Hungary_Mako_EBA
Netherlands_Bell_Beaker
England_CA_EBA
England_Bell_Beaker
Scotland_LBA
Germany_Corded_Ware
England_Roman
Hungary_Sopot_LN
England_MBA
Poland_Chopice_Vesele_Culture
Scotland_CA_EBA
Orkney_LinksOfNoltland
Hungary_Bell_Beaker_EBA
CPC
Ireland_BA
Czech_Starounetice_EBA
England_Meso
Croatia_MBA
Czech_EBA
Scotland_MBA
Czech_Bell_Beaker
Poland_Globular_Amphora
Scotland_Bell_Beaker
Germany_Bell_Beaker
Poland_Bell_Beaker
England_IA

E

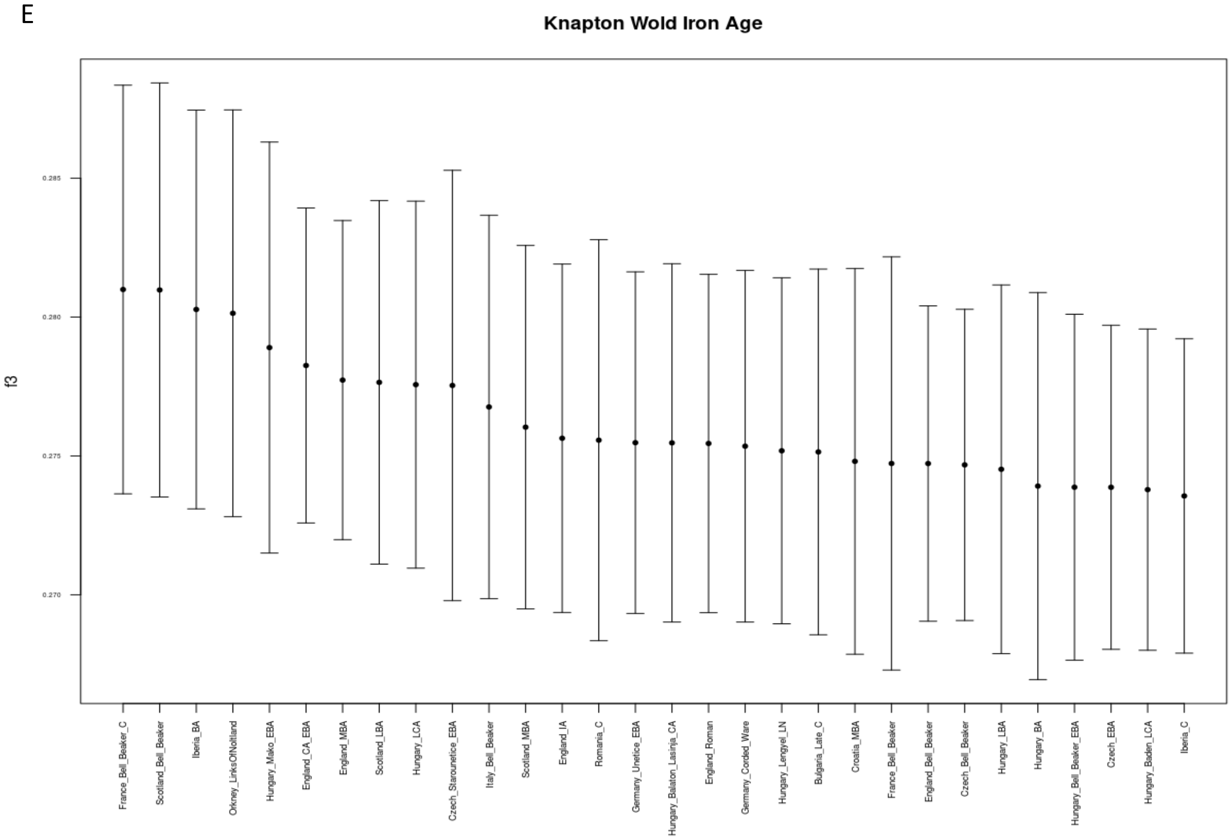
Knapton Wold Iron Age
f3
France_Bell_Beaker_C
Scotland_Bell_Beaker
Iberia_BA
Orkney_LinksOfNoltland
Hungary_Mako_EBA
England_CA_EBA
England_MBA
Scotland_LBA
Hungary_LCA
Czech_Starounetice_EBA
Italy_Bell_Beaker
Scotland_MBA
England_IA
Romania_C
Germany_Unetice_EBA
Hungary_Balaton_Lasinja_CA
England_Roman
Germany_Corded_Ware
Hungary_Lengyel_LN
Bulgaria_Late_C
Croatia_MBA
France_Bell_Beaker
England_Bell_Beaker
Czech_Bell_Beaker
Hungary_LBA
Hungary_BA
Hungary_Bell_Beaker_EBA
Czech_EBA
Hungary_Baden_LCA
Iberia_C

F

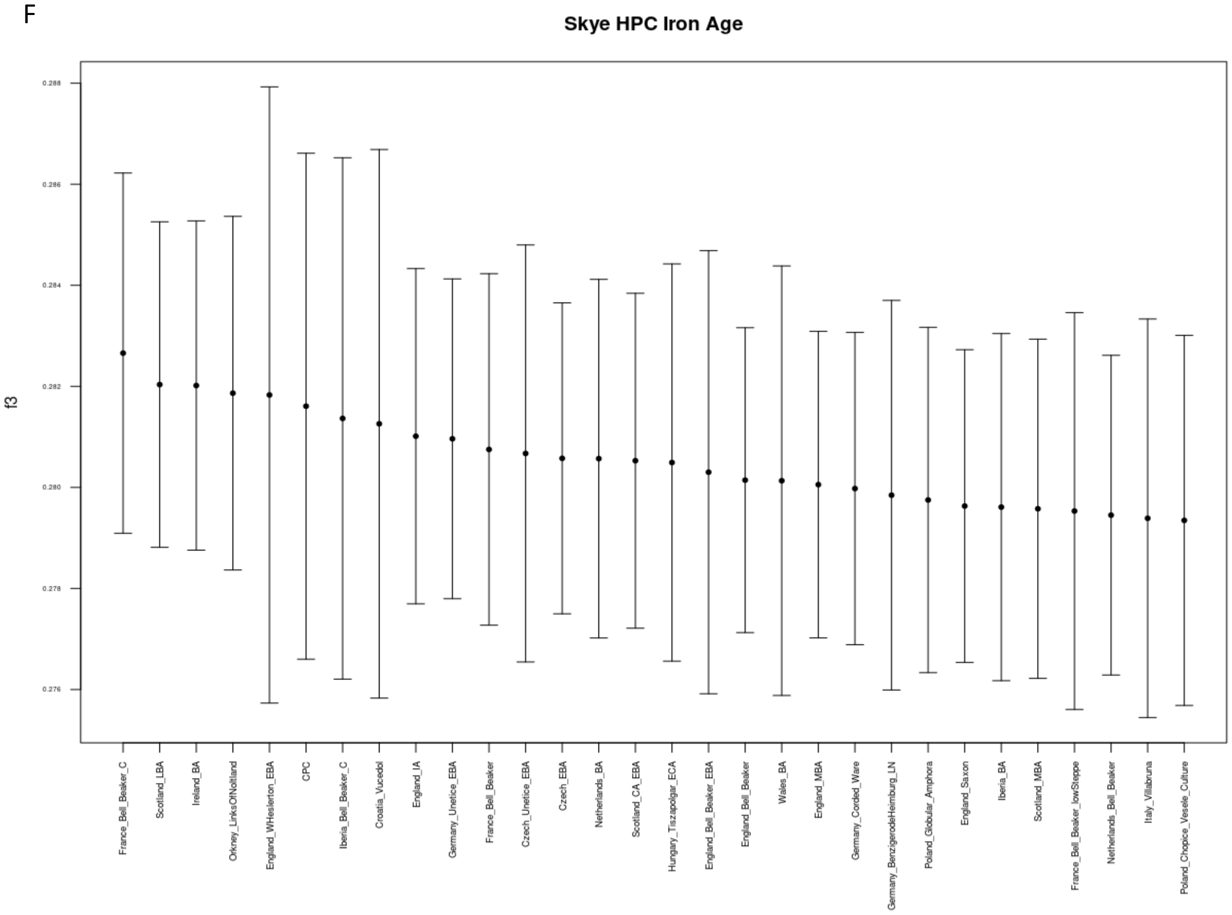
Skye HPC Iron Age
f3
France_Bell_Beaker_C
Scotland_LBA
Ireland_BA
Orkney_LinksOfNoltland
England_WHeslerton_EBA
CPC
Iberia_Bell_Beaker_C
Croatia_Vucedol
England_IA
Germany_Unetice_EBA
France_Bell_Beaker
Czech_Unetice_EBA
Czech_EBA
Netherlands_BA
Scotland_CA_EBA
Hungary_Tiszapolgar_ECA
England_Bell_Beaker_EBA
England_Bell_Beaker
Wales_BA
England_MBA
Germany_Corded_Ware
Germany_BenzigerodeHeimburg_LN
Poland_Globular_Amphora
England_Saxon
Iberia_BA
Scotland_MBA
France_Bell_Beaker_lowSteppe
Netherlands_Bell_Beaker
Italy_Villabruna
Poland_Chopice_Vesele_Culture

G

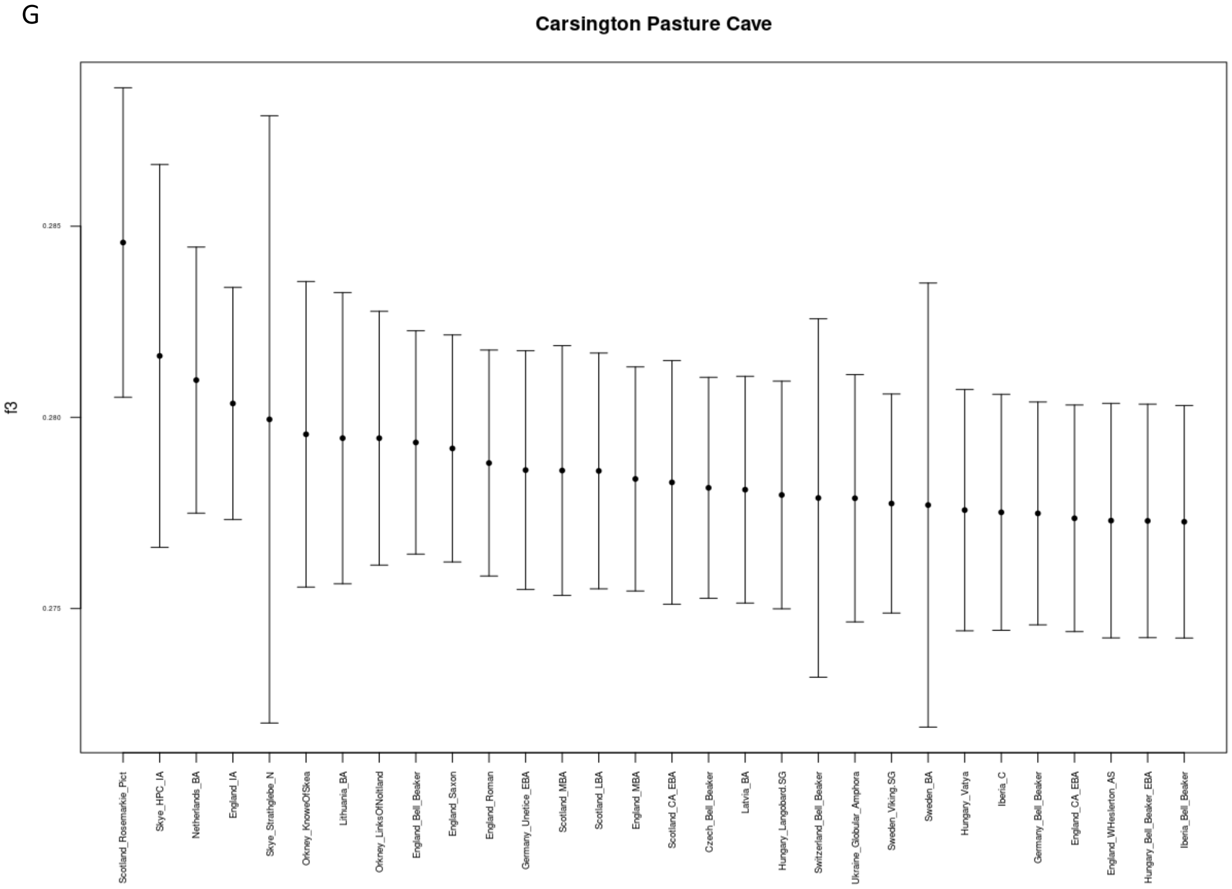

Carsington Pasture Cave
f3
0.285
0.280
0.275
Scotland_Rosemarkie_Pict
Skye_HPC_IA
Netherlands_BA
England_IA
Skye_Strathglebe_N
Orkney_KnoweOfSkea
Lithuania_BA
Orkney_LinksOfNoltland
England_Bell_Beaker
England_Saxon
England_Roman
Germany_Unetice_EBA
Scotland_MBA
Scotland_LBA
England_MBA
Scotland_CA_EBA
Czech_Bell_Beaker
Latvia_BA
Hungary_Langobard.SG
Switzerland_Bell_Beaker
Ukraine_Globular_Amphora
Sweden_Viking.SG
Sweden_BA
Hungary_Vatya
Iberia_C
Germany_Bell_Beaker
England_CA_EBA
England_WHeslerton_AS
Hungary_Bell_Beaker_EBA
Iberia_Bell_Beaker

H

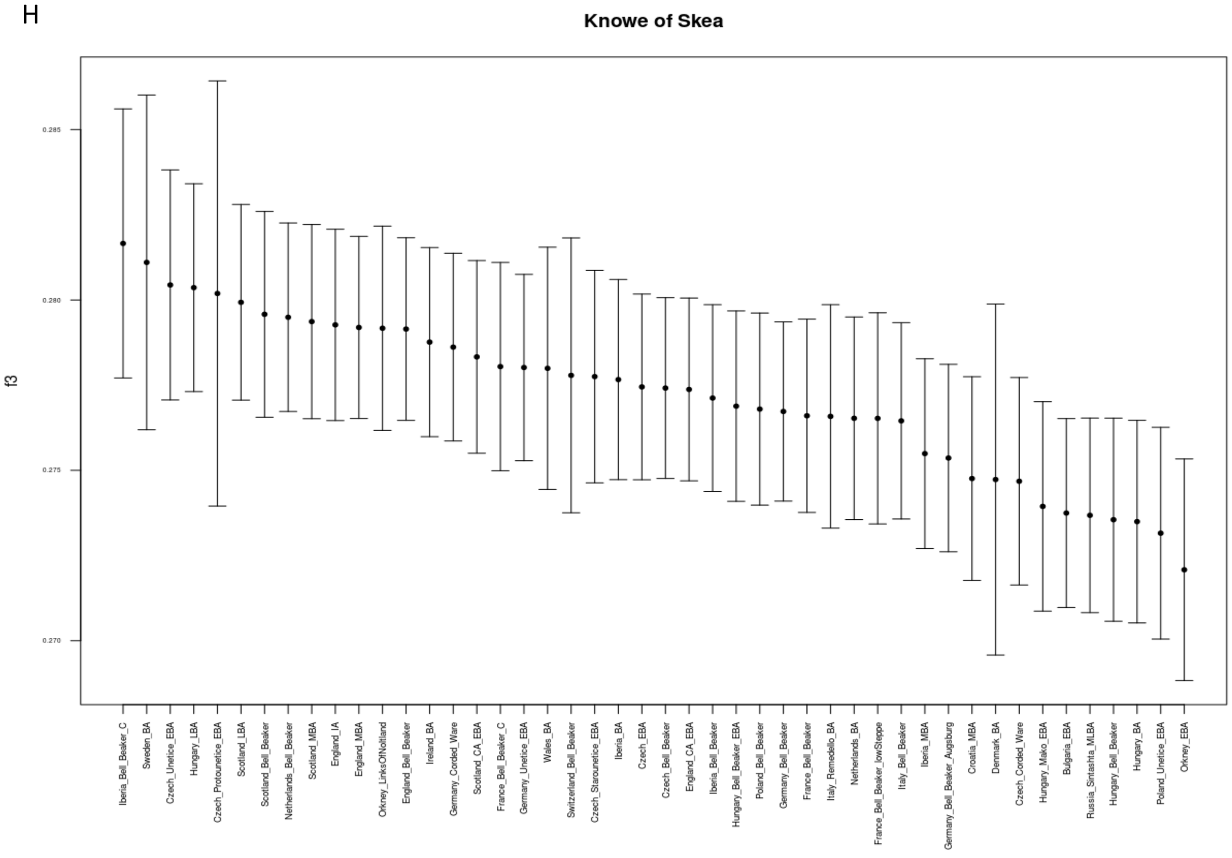

Knowe of Skea
f3
0.285
0.280
0.275
0.270
Iberia_Bell_Beaker_C
Sweden_BA
Czech_Unetice_EBA
Hungary_LBA
Czech_Protounetice_EBA
Scotland_LBA
Scotland_Bell_Beaker
Netherlands_Bell_Beaker
Scotland_MBA
England_IA
England_MBA
Orkney_LinksOfNoltland
England_Bell_Beaker
Ireland_BA
Germany_Corded_Ware
Scotland_CA_EBA
France_Bell_Beaker_C
Germany_Unetice_EBA
Wales_BA
Switzerland_Bell_Beaker
Czech_Starounetice_EBA
Iberia_BA
Czech_EBA
Czech_Bell_Beaker
England_CA_EBA
Iberia_Bell_Beaker
Hungary_Bell_Beaker_EBA
Poland_Bell_Beaker
Germany_Bell_Beaker
France_Bell_Beaker
Italy_Remedello_BA
Netherlands_BA
France_Bell_Beaker_lowSteppe
Italy_Bell_Beaker
Iberia_MBA
Germany_Bell_Beaker_Augsburg
Croatia_MBA
Denmark_BA
Czech_Corded_Ware
Hungary_Mako_EBA
Bulgaria_EBA
Russia_Sintashta_MLBA
Hungary_Bell_Beaker
Hungary_BA
Poland_Unetice_EBA
Orkney_EBA

I

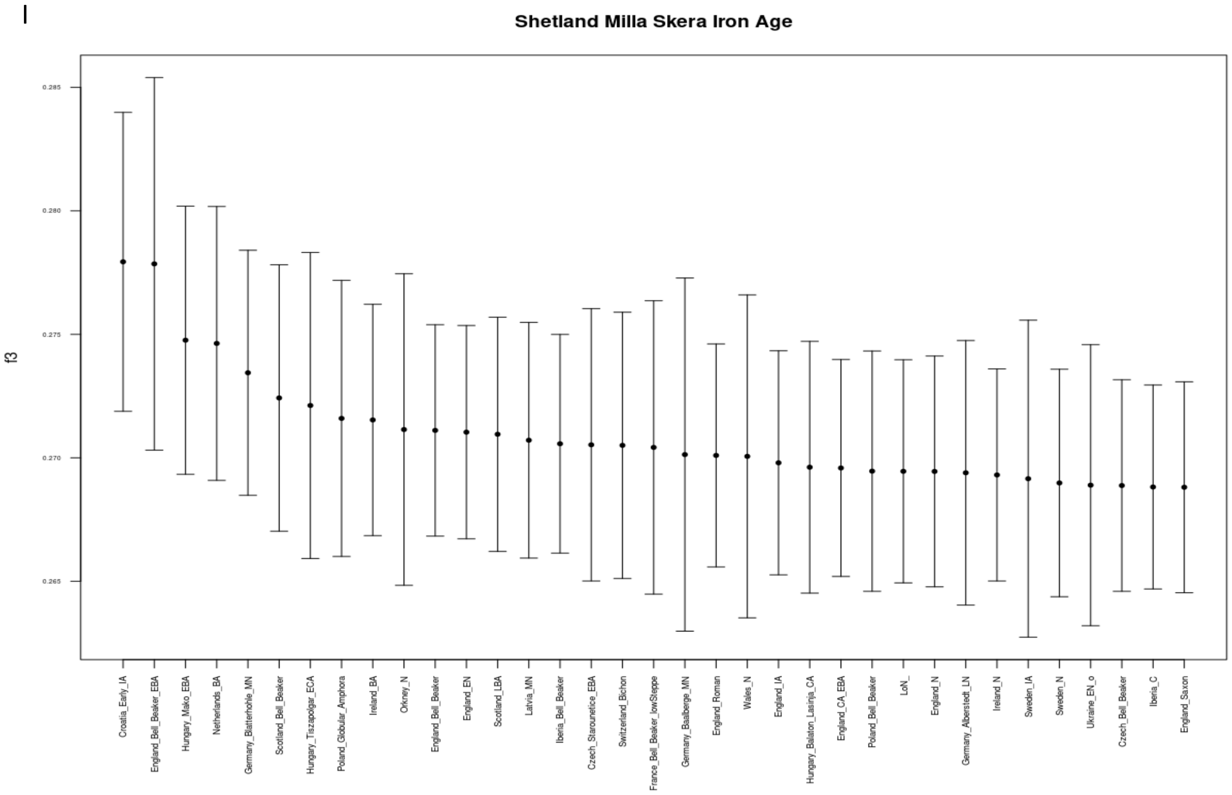
Shetland Milla Skera Iron Age
f3
0.285
0.280
0.275
0.270
0.265
Croatia_Early_IA
England_Bell_Beaker_EBA
Hungary_Mako_EBA
Netherlands_BA
Germany_Blatterhohle_MN
Scotland_Bell_Beaker
Hungary_Tiszapolgar_ECA
Poland_Globular_Amphora
Ireland_BA
Orkney_N
England_Bell_Beaker
England_EN
Scotland_LBA
Latvia_MN
Iberia_Bell_Beaker
Czech_Starounetice_EBA
Switzerland_Bichon
France_Bell_Beaker_lowSteppe
Germany_Baalberge_MN
England_Roman
Wales_N
England_IA
Hungary_Balaton_Lasinja_CA
England_CA_EBA
Poland_Bell_Beaker
LoN_
England_N
Germany_Alberstedt_LN
Ireland_N
Sweden_IA
Sweden_N
Ukraine_EN_o
Czech_Bell_Beaker
Iberia_C
England_Saxon

J

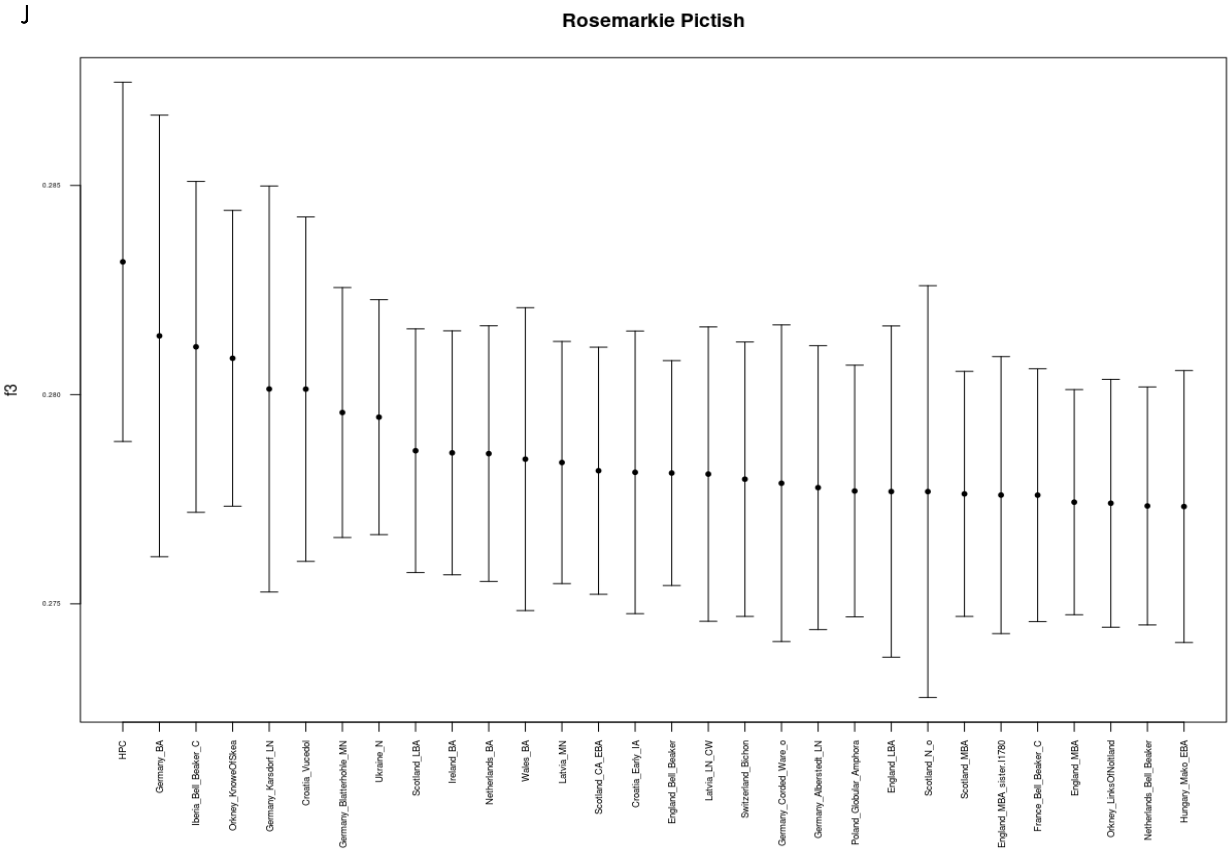
Rosemarkie Pictish
f3
0.285
0.280
0.275
HPC
Germany_BA
Iberia_Bell_Beaker_C
Orkney_KnoweOfSkea
Germany_Karsdorf_LN
Croatia_Vucedol
Germany_Blatterhohle_MN
Ukraine_N
Scotland_LBA
Ireland_BA
Netherlands_BA
Wales_BA
Latvia_MN
Scotland_CA_EBA
Croatia_Early_IA
England_Bell_Beaker
Latvia_LN_CW
Switzerland_Bichon
Germany_Corded_Ware_o
Germany_Alberstedt_LN
Poland_Globular_Amphora
England_LBA
Scotland_N_o
Scotland_MBA
England_MBA_sister.I1780
France_Bell_Beaker_C
England_MBA
Orkney_LinksOfNoltland
Netherlands_Bell_Beaker
Hungary_Mako_EBA

K

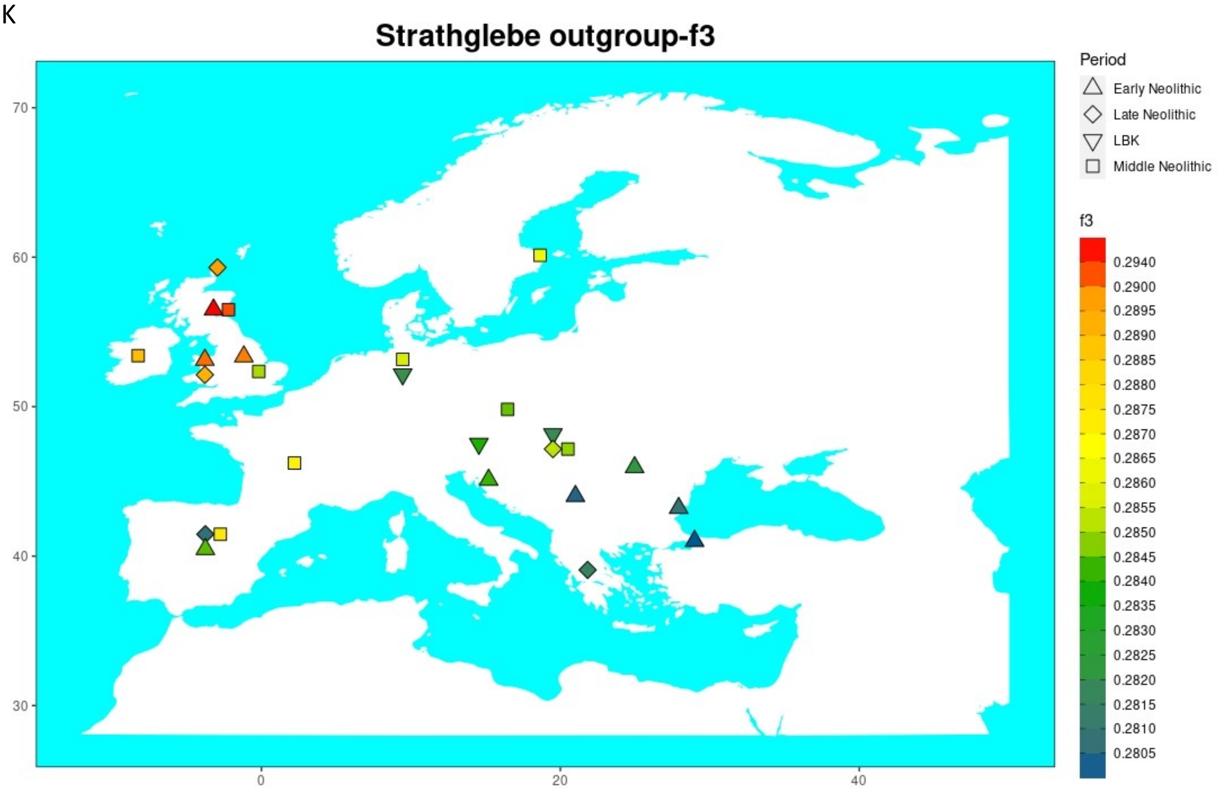

Fig. S5. (A-J) Outgroup-*f3* plots each site displaying the highest *f3* results for tests with over 20,000 SNPs. (K) Map displaying the outgroup-*f3* statistics for the Neolithic Strath Glebe individual from Skye, western Scotland. Note the closer relationship with other Early Neolithic Scottish individuals in comparison with those from Wales and England; and with Middle Neolithic individuals of the Atlantic façade, in comparison with other European Neolithic individuals.

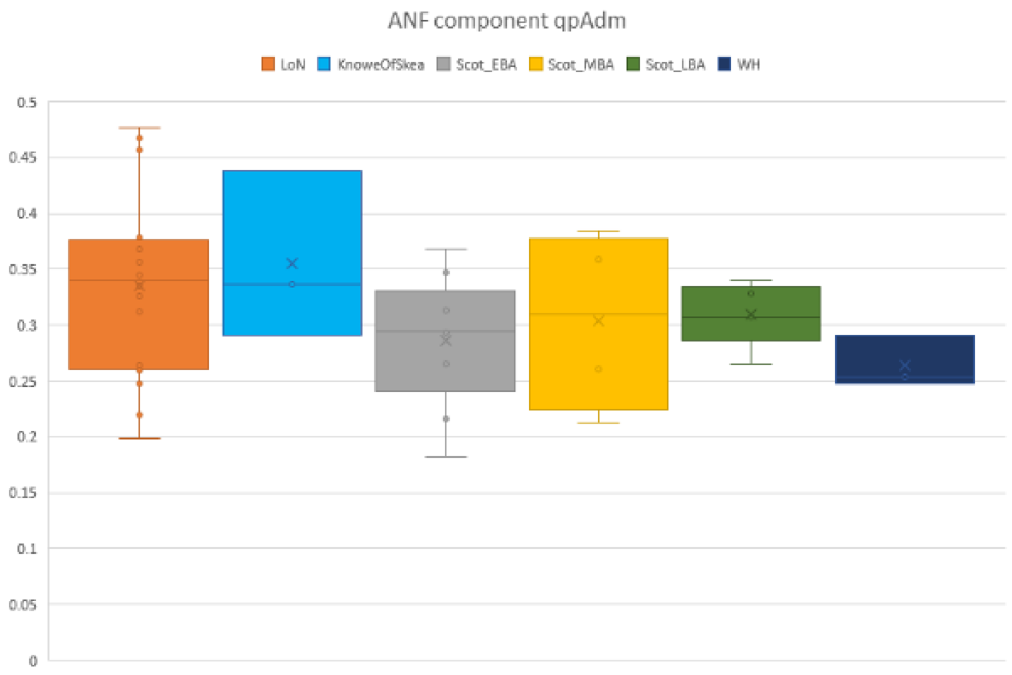

Fig. S6A. ANF fraction estimated using *qpAdm* from Bronze Age and Iron Age samples.

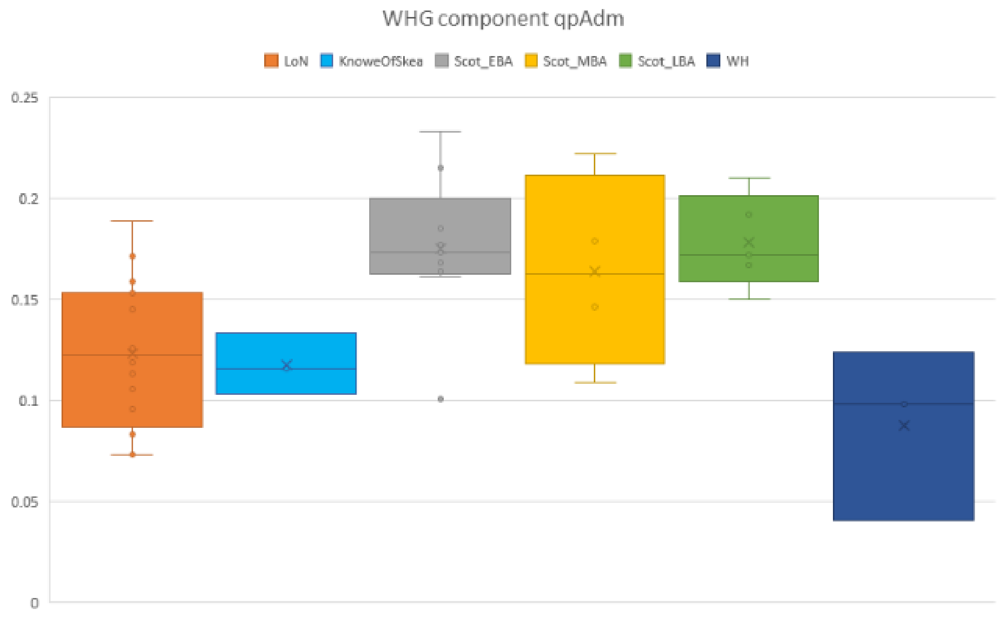

Fig. S6B. WHG fraction estimated using *qpAdm* from Bronze Age and Iron Age samples.

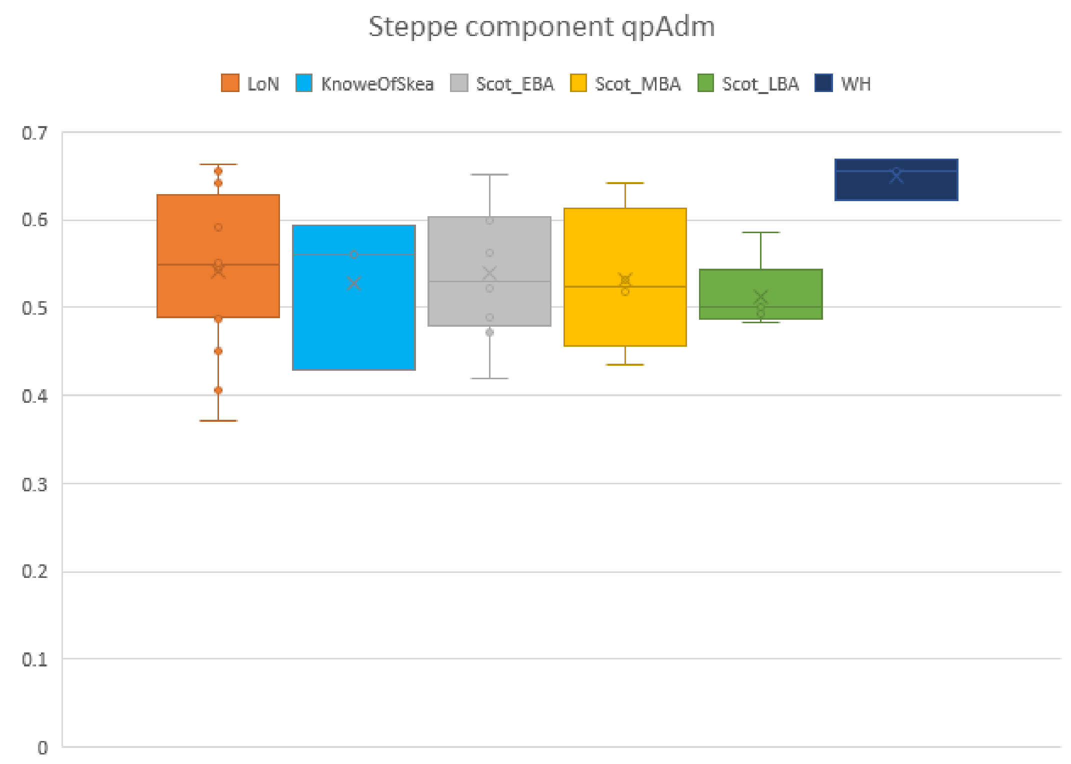

Fig. S6C. Yamnaya fraction estimated using *qpAdm* from Bronze Age and Iron Age samples.

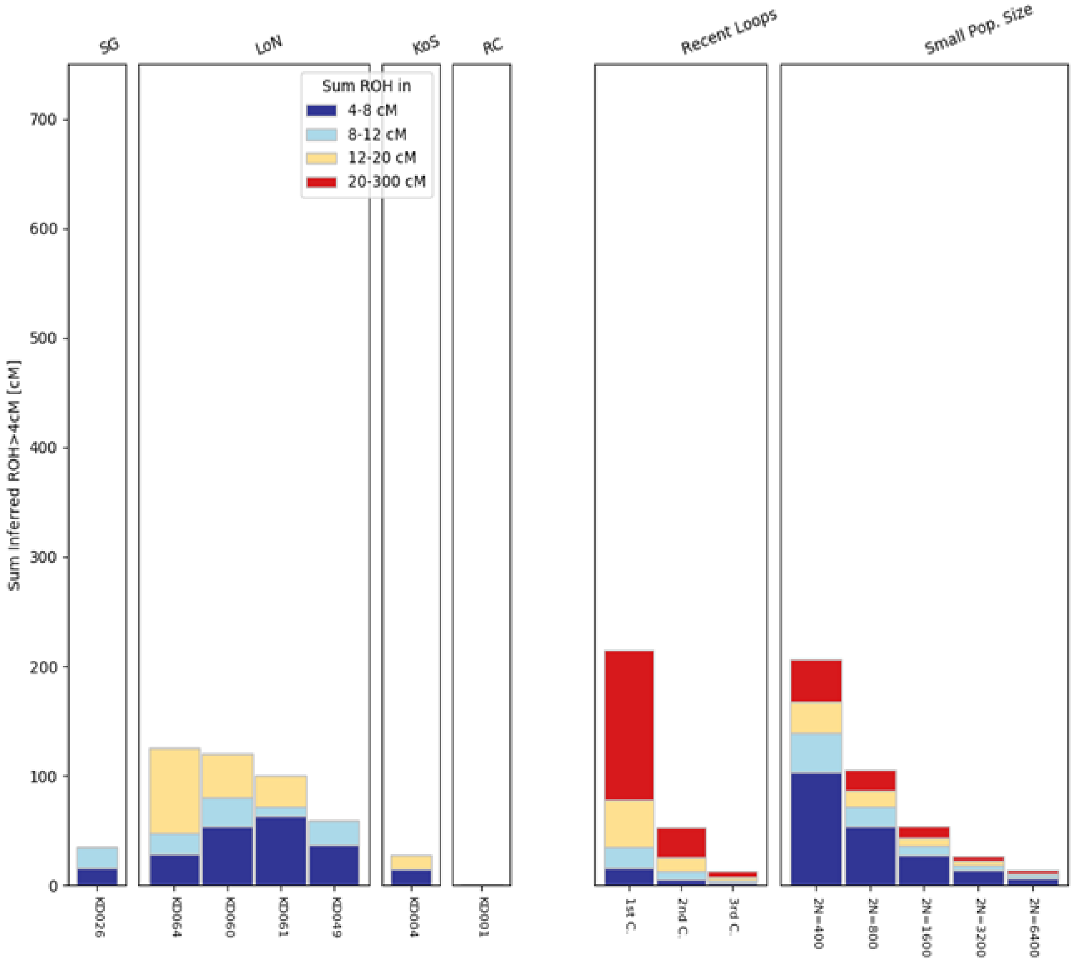

Fig. S7. Runs of homozygosity calculated using hapROH on newly reported samples with >400,000 SNPs against the 1240K SNP array. The LoN show relatively large amounts of short ROH, indicating a small effective population size and possible bottleneck. No long runs of homozygosity are detected, suggesting that there was no recent consanguinity.

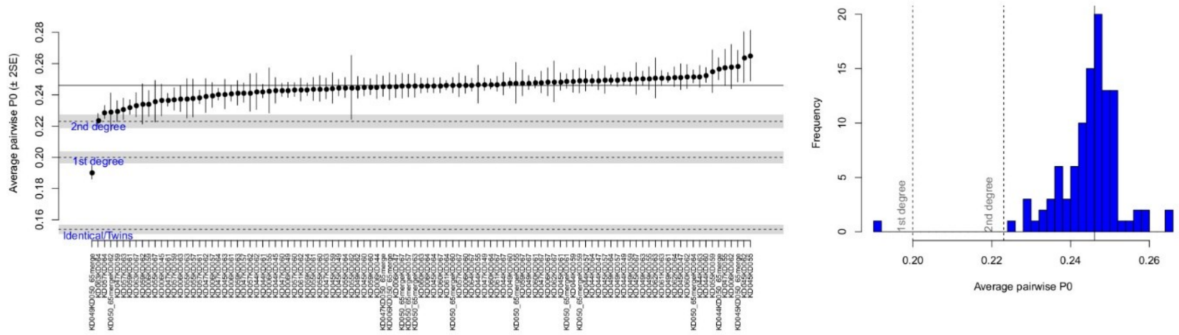

Fig. S8A. READ results for the Links of Noltland population.

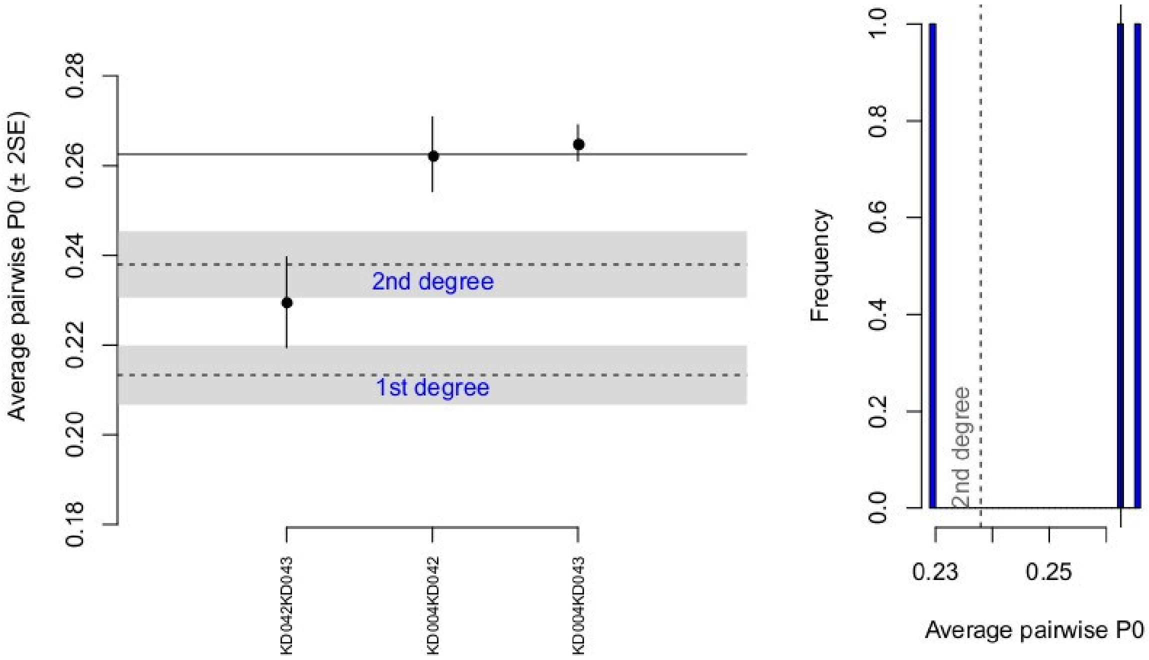

Fig. S8B. READ results for the Knowe of Skea population.

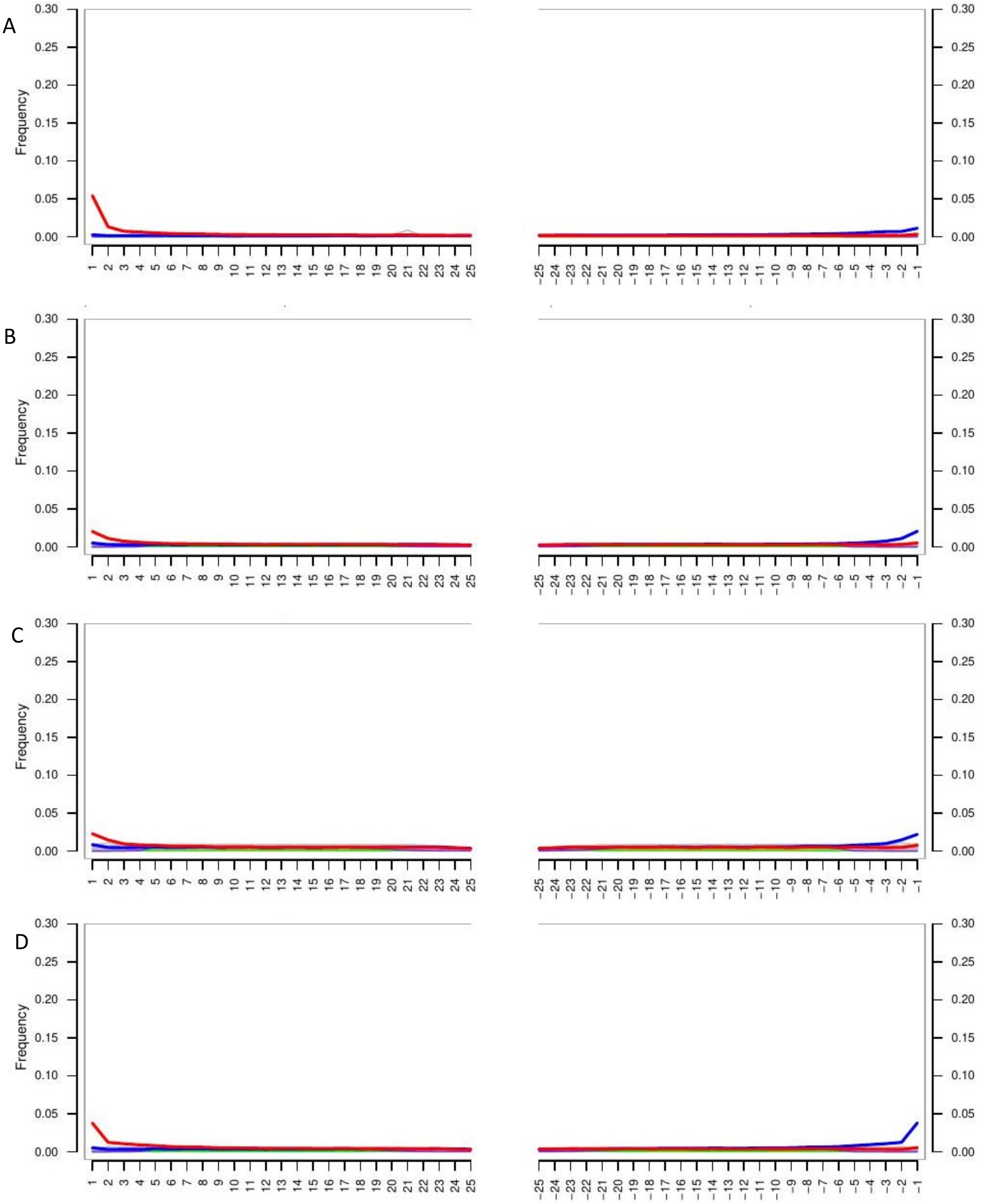
A
B
C
D
Frequency
0.30
0.25
0.20
0.15
0.10
0.05
0.00

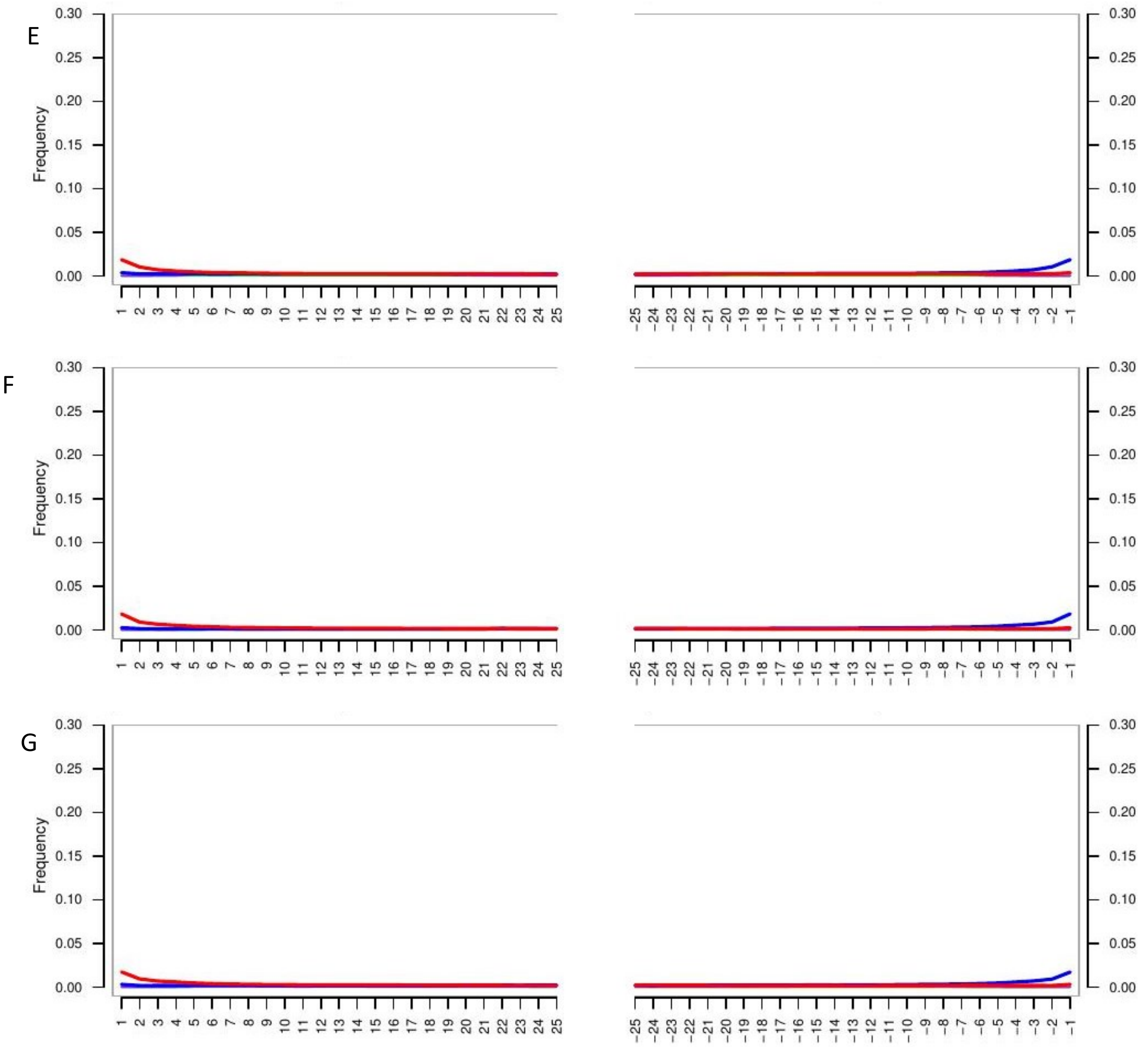

Fig S9. MapDamage mis-incorporation plots for a representative subset of the total libraries processed. The y-axis represents the frequency of substitutions, and the x-axis represents the number of bases from the 5’ end (left) and the 3’ end (right). Red represents C to T substitutions, and blue G to A substitutions. The presence of increased C to T substitutions compared to other types of transition is characteristic of DNA damage (read at the 5’ end as G to A). As expected for UDG-treated samples, there is rather little C to T damage present, but it is nevertheless still clearly evident in each of the plots. (A) KD004. (B) KD026. (C) KD047. (D) KD049. (E) KD060. (F) KD064. (G) KD067.

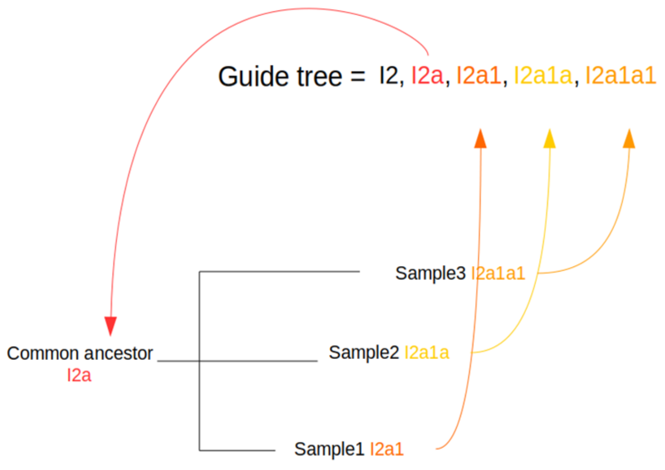

Fig. S10. Process to annotate automatically a phylogenetic tree.

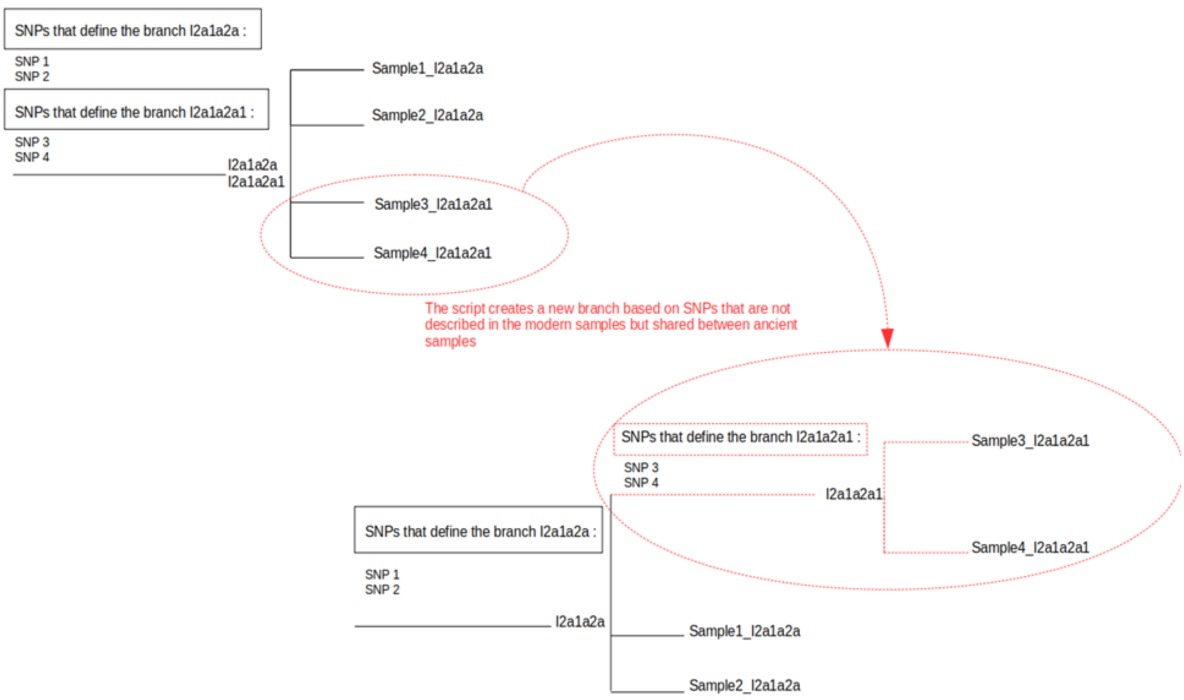

Fig. S11. Procedure to create new branch using SNPs only present in ancient samples.

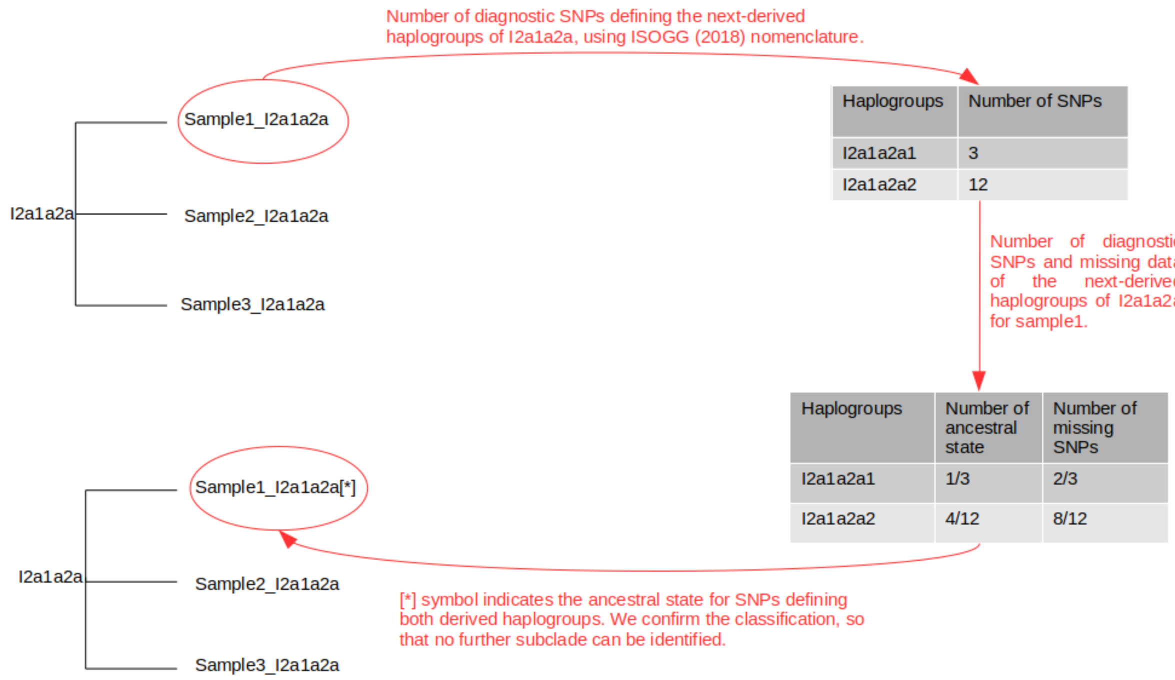

Fig. S12A. Confirmation of haplogroup determination.

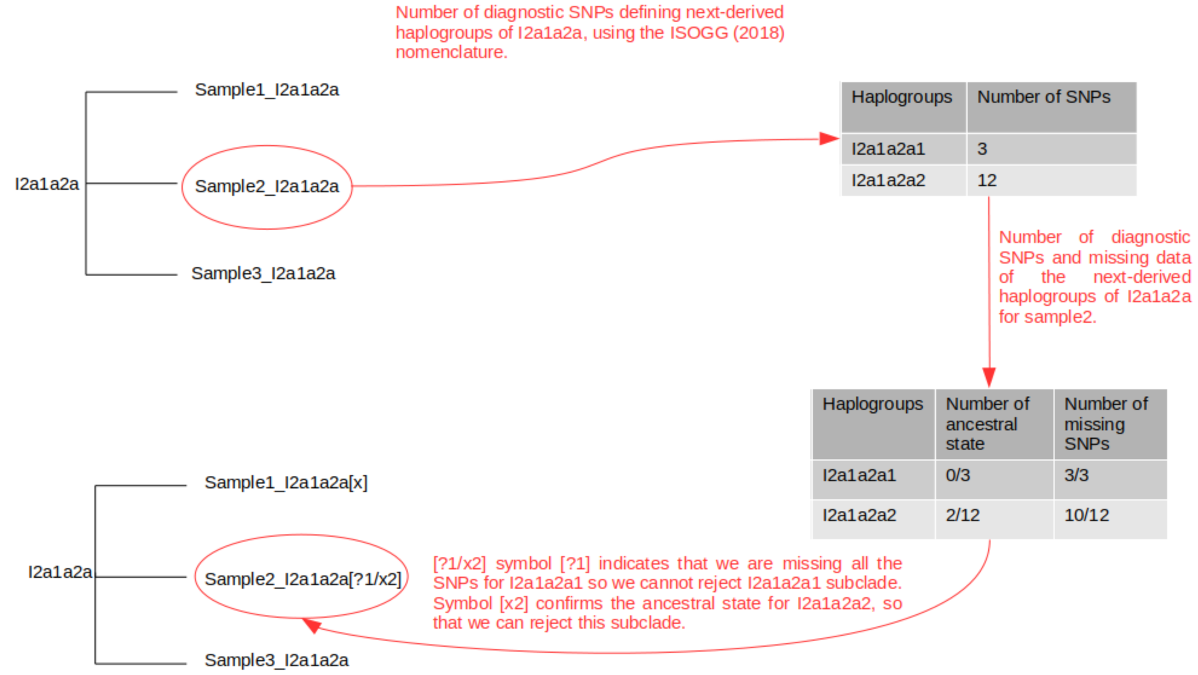

Fig. S12B. Partial confirmation of haplogroup determination.

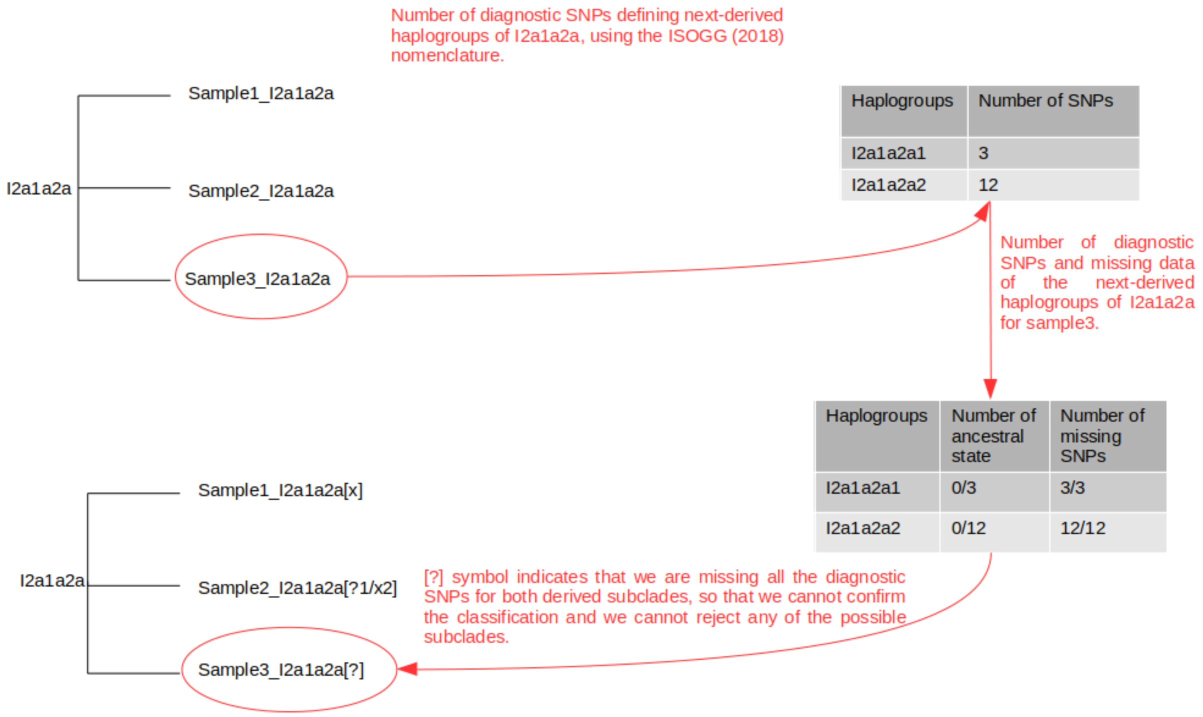

Fig. S12C. Uncertainty in haplogroup determination for all subclades.

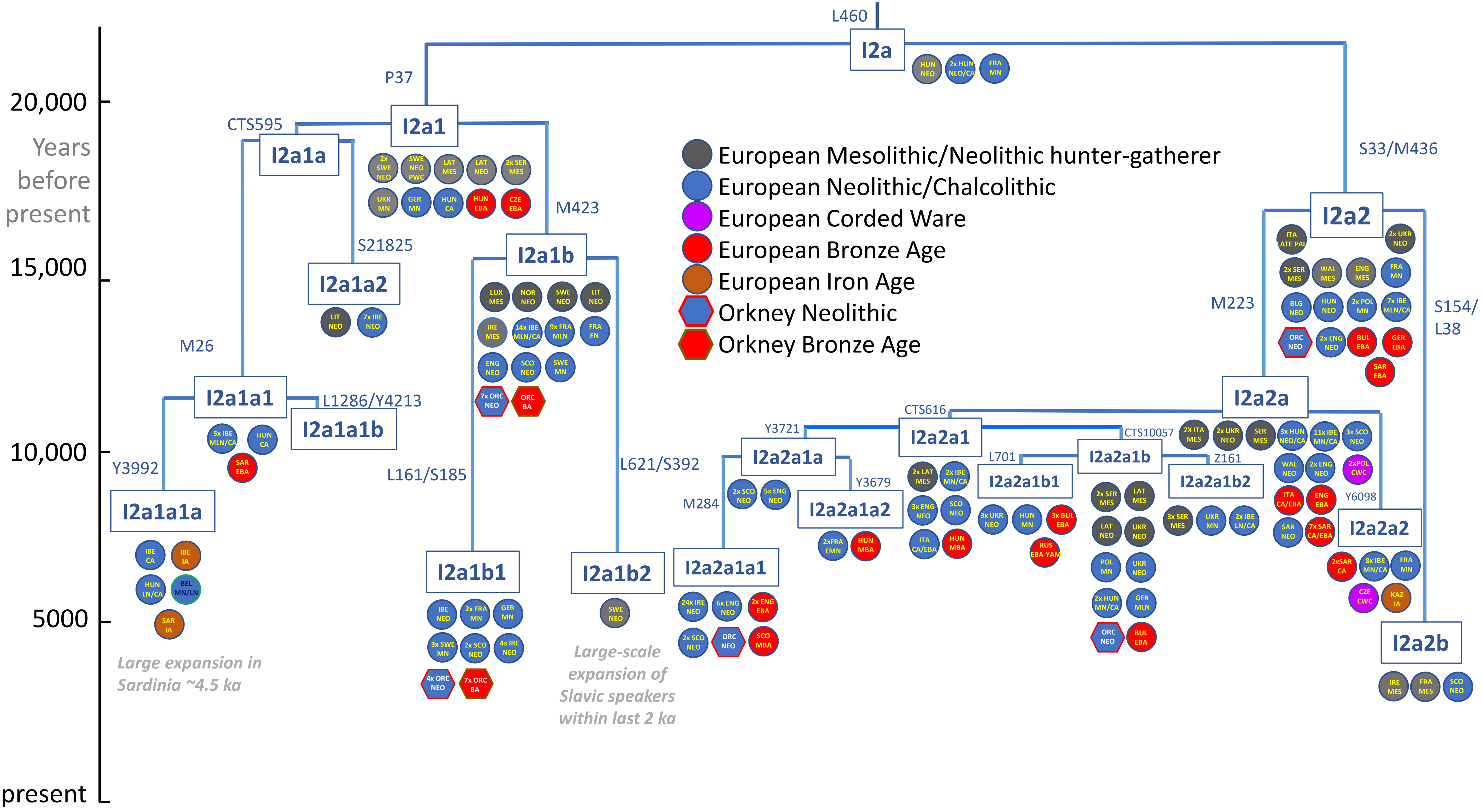

20,000
15,000
10,000
5000
present
Years before present
L460
I2a
HUN NEO
2x HUN NEO/CA
FRA MN
P37
I2a1
2x SWE NEO
SWE NEO PWC
LAT MES
LAT NEO
2x SER MES
UKR MN
GER MN
HUN CA
HUN EBA
CZE EBA
CTS595
I2a1a
S21825
I2a1a2
LIT NEO
7x IRE NEO
M26
I2a1a1
5x IBE MLN/CA
HUN CA
SAR EBA
L1286/Y4213
I2a1a1b
Y3992
I2a1a1a
IBE CA
IBE IA
HUN LN/CA
BEL MN/LN
SAR IA
Large expansion in Sardinia ~4.5 ka
M423
I2a1b
LUX MES
NOR NEO
SWE NEO
LIT NEO
IRE MES
14x IBE MLN/CA
9x FRA MN
FRA EN
ENG NEO
SCO NEO
SWE MN
7x ORC NEO
ORC BA
L161/S185
I2a1b1
IBE NEO
2x FRA MN
GER MN
3x SWE MN
2x SCO NEO
4x IRE NEO
4x ORC NEO
7x ORC BA
L621/S392
I2a1b2
SWE NEO
Large-scale expansion of Slavic speakers within last 2 ka
European Mesolithic/Neolithic hunter-gatherer
European Neolithic/Chalcolithic
European Corded Ware
European Bronze Age
European Iron Age
Orkney Neolithic
Orkney Bronze Age
S33/M436
I2a2
ITA LATE PAL
2x UKR NEO
2x SER MES
WAL MES
ENG MES
FRA MN
BLG NEO
HUN NEO
2x POL MN
7x IBE MLN/CA
ORC NEO
2x ENG NEO
BUL EBA
GER EBA
SAR EBA
M223
S154/L38
I2a2a
CTS616
2X ITA MES
2x UKR NEO
SER MES
3x HUN NEO/CA
11x IBE MN/CA
3x SCO NEO
WAL NEO
2x ENG NEO
2xPOL CWC
ITA CA/EBA
ENG EBA
SAR NEO
7x SAR CA/EBA
Y6098
I2a2a2
2xSAR CA
8x IBE MN/CA
FRA MN
CZE CWC
KAZ IA
I2a2b
IRE MES
FRA MES
SCO NEO
Y3721
I2a2a1
2x LAT MES
2x IBE MN/CA
3x ENG NEO
SCO NEO
ITA CA/EBA
HUN MBA
I2a2a1a
2x SCO NEO
5x ENG NEO
Y3679
I2a2a1a2
2xFRA EMN
HUN MBA
M284
I2a2a1a1
24x IRE NEO
6x ENG NEO
2x ENG EBA
2x SCO NEO
ORC NEO
SCO MBA
CTS10057
I2a2a1b
L701
I2a2a1b1
3x UKR NEO
HUN MN
3x BUL EBA
RUS EBA-YAM
2x SER MES
LAT MES
LAT NEO
UKR NEO
POL MN
UKR NEO
2x HUN MN/CA
GER MLN
ORC NEO
BUL EBA
Z161
I2a2a1b2
3x SER MES
UKR MN
2x IBE LN/CA

Fig. S13. Phylochronology of Y-chromosome haplogroup I2a-L460. Schematic phylogenetic tree of Y-chromosome haplogroup I2a, showing published and new prehistoric sample data, from a database of 667 published adequately resolved West Eurasian Y-chromosome haplotypes. Haplogroups are labelled according to the widely used ISOGG 2017 nomenclature.

Samples are colour-coded for broad archaeological period (Late Palaeolithic/Mesolithic and Neolithic hunter-gatherers, Neolithic/Chalcolithic, Corded Ware, Bronze Age, Iron Age) with Orcadian Neolithic and Bronze Age distinguished by hexagonal outline, white font and distinct borders. Each circle represents a Y-chromosome haplotype and is labelled for archaeological subdivision (Late Pal = Late Upper Palaeolithic, Mes = Mesolithic, Neo = Neolithic, MN = Middle Neolithic, MLN = Middle/Late Neolithic, LN = Late Neolithic, CA = Chalcolithic, BA = Bronze Age, IA = Iron Age), and for region (ORC = Orkney, SCO = Scotland, ENG = England, WAL = Wales, IRE = Eire, FRA = France, IBE = Iberia, LUX = Luxembourg, GER = Germany, NOR = Norway, SWE = Sweden, POL = Poland, CZE = Czech Republic, HUN = Hungary, ITA = Italy, SER = Serbia, BUL = Bulgaria, LIT = Lithuania, LAT = Latvia, UKR = Ukraine, RUS = Russia, KAZ = Kazakhstan). Note that we have merged CA with Neolithic for Iberia, but with the BA elsewhere in Europe, given their different demographic trajectories.

Timescale shows years before present for the nodes in the tree based on the Y-chromosome molecular clock (YFull 2020). Note that samples are positioned phylogenetically at the nodes of the tree, regardless of the age of the sample. Note that some samples appear on the tree earlier than their true age because intra-specific haplotypes may persist in the population for many thousands of years; more unusually, some may appear later, which may be either due to mis-classification/poor typing or an under-estimate of the node age using modern data. Caution is warranted, in general, because of the imperfect resolution of ancient DNA analysis, due to missing SNPs, which may result in samples being assigned an artefactually less derived position (see legend to Fig. S15).

Note that I2a1-P37 and I2a2-S33 display distinctive phylogeographic and phylochronological patterns. Amongst Mesolithic and Neolithic hunter-gatherers, I2a1-P37 is distributed across northern, southeast and central Europe (I2a1b-M423 is represented by the Loschbour individual from Mesolithic Luxembourg at ~8 ka), but seems to be absent from Iberia which was dominated by C1a2-V20 lineages until the arrival of Neolithic farmers dispersing along the Mediterranean route (91, 144). I2a1b-M423 is heavily represented in both Iberian and French Neolithic samples, as well as in Neolithic Britain and Sweden, but has not been seen in prehistoric Italy, which has so far been represented only by I2a2-S33 (145): the oldest I2a2 (and I2a) lineage is from Late Glacial Italy) and R1b – ancestral for M269 (76) – or in the prehistoric Balkans, which is similarly largely a mix of I2a2-S33 and ancestral R1b1a (87), but with a few representatives of I2a1-P37.

I2a1b-M423 therefore seems most likely to have been assimilated from hunter-gatherers in the western Mediterranean, most likely in southern France before the divergence to Iberia in the west (although I2a1b-M423 may have only arrived in Iberia with a second wave in the Middle Neolithic) and northern France, Britain and Scandinavia in the north. Interestingly it is very rare in the English Neolithic (and absent to date from the Neolithic of Wales, although there are very few data) but present, albeit again as a minority lineage, in both Neolithic and modern Ireland (101). Although the pattern may be the result of drift, it suggests a possible dispersal from northwest France to Ireland, Scotland and Orkney via the western coast of Britain. Much of Neolithic Britain mostly carries I2a2-S33 lineages, which may have been introduced separately into southeast England from further east on the Continent, perhaps with a slightly earlier wave (146).

Note also that although modern data are not included (see YFull tree for a detailed portrait of the modern phylogeography), I2a1b2 seems to be associated with the medieval expansion of Dinaric Slavic speakers (147); also that I2a1a1 has undergone a massive expansion in Sardinia, possibly dating to the Neolithic or the Bronze Age (148).

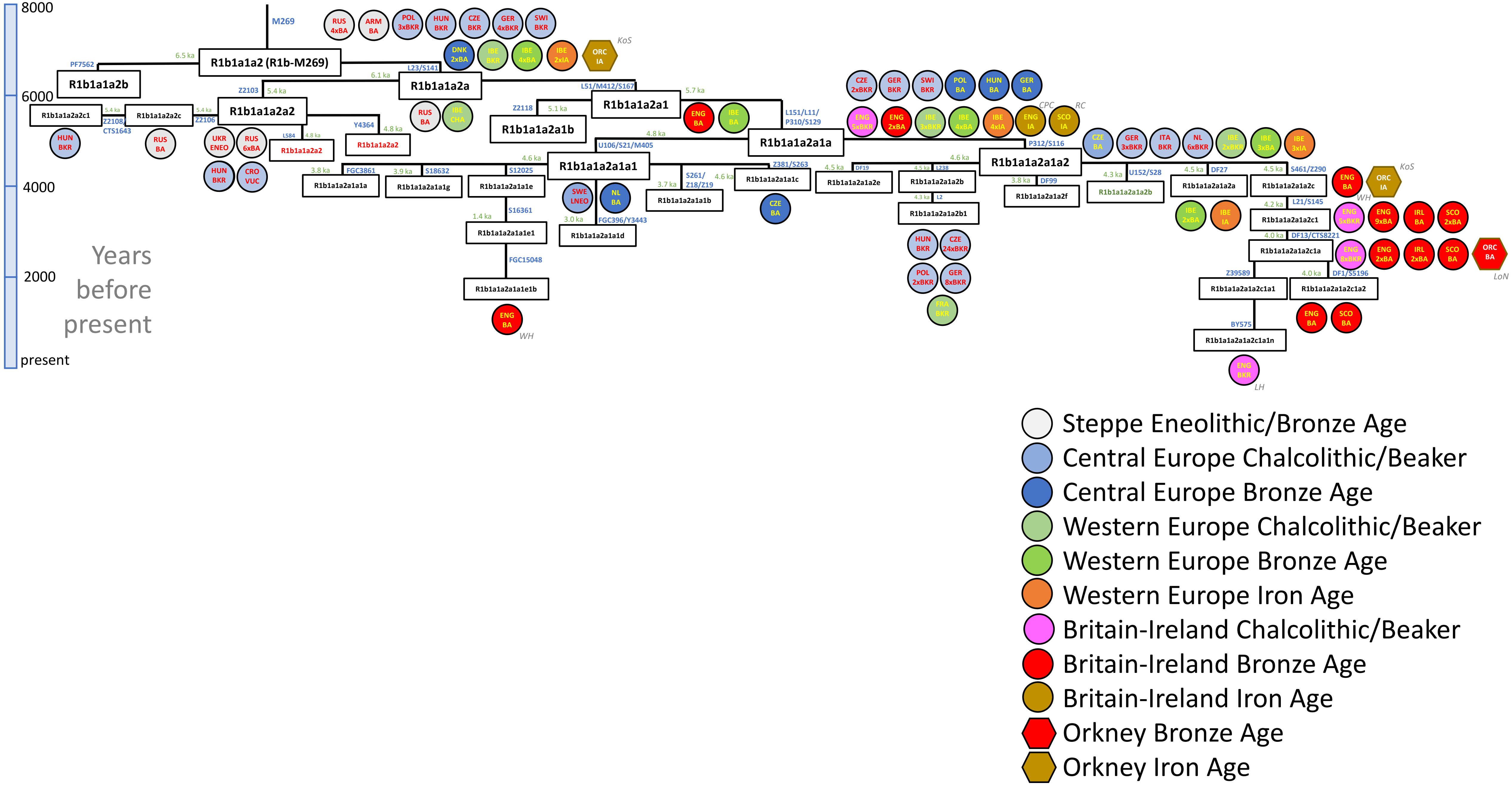

8000
6000
4000
2000
present
Years before present
M269
R1b1a1a2 (R1b-M269)
R1b1a1a2b
R1b1a1a2a
R1b1a1a2a2
R1b1a1a2a2c
R1b1a1a2a2c1
R1b1a1a2a1
R1b1a1a2a1b
R1b1a1a2a1a
R1b1a1a2a1a1
R1b1a1a2a1a2
R1b1a1a2a1a1a
R1b1a1a2a1a1g
R1b1a1a2a1a1e
R1b1a1a2a1a1e1
R1b1a1a2a1a1e1b
R1b1a1a2a1a1d
R1b1a1a2a1a1b
R1b1a1a2a1a1c
R1b1a1a2a1a2e
R1b1a1a2a1a2b
R1b1a1a2a1a2b1
R1b1a1a2a1a2f
R1b1a1a2a1a2b
R1b1a1a2a1a2a
R1b1a1a2a1a2c
R1b1a1a2a1a2c1
R1b1a1a2a1a2c1a
R1b1a1a2a1a2c1a1
R1b1a1a2a1a2c1a2
R1b1a1a2a1a2c1a1n
Steppe Eneolithic/Bronze Age
Central Europe Chalcolithic/Beaker
Central Europe Bronze Age
Western Europe Chalcolithic/Beaker
Western Europe Bronze Age
Western Europe Iron Age
Britain-Ireland Chalcolithic/Beaker
Britain-Ireland Bronze Age
Britain-Ireland Iron Age
Orkney Bronze Age
Orkney Iron Age

Fig. S14. Phylochronology of Y-chromosome haplogroup R1b-M269. Schematic phylogenetic tree of Y-chromosome haplogroup R1b-M269, showing published and new prehistoric sample data, from a database of West Eurasian Y-chromosome haplotypes, mainly from western Europe (12, 87, 91). Samples are colour-coded for broad archaeological period (Steppe Eneolithic/Bronze Age, Chalcolithic/Beaker, Bronze Age, Iron Age) with Orcadian Bronze Age and Iron Age distinguished by hexagonal outline and white font. Each circle represents a Y-chromosome haplotype and is labelled for archaeological subdivision (Eneo = Eneolithic, VUC = Croatian Vucedol Eneolithic, LN = Late Neolithic, CHA = Chalcolithic, BA = Bronze Age, IA = Iron Age), and for region (ORC = Orkney, SCO = Scotland, ENG = England, WAL = Wales, IRE = island of Ireland, FRA = France, IBE = Iberia, NL = Netherlands, SWI = Switzerland, GER = Germany, DNK = Denmark, SWE = Sweden, ITA = Italy, CRO = Croatia, CZE = Czech Republic, HUN = Hungary, POL = Poland, UKR = Ukraine, RUS = Russia, ARM = Armenia). Timescale shows years before present for the nodes in the tree based on the Y-chromosome molecular clock (YFull 2020). New data: LoN = Links of Noltland; KoS = Knowe of Skea; LH = Low Hauxley; WH = West Heslerton; CPC = Carsington Pasture Cave; RC = Rosemarkie Cave.

Note that samples are positioned phylogenetically at the nodes of the tree (for practical reasons, some are above and some below), regardless of the age of the sample. Some samples appear on the tree earlier than their archaeological age because intra-specific haplotypes may persist in the population for many thousands of years. Caution is warranted, however, because of the imperfect resolution of ancient DNA analysis, due to missing SNPs, which may result in samples being assigned an artefactually less derived position. This may especially apply to samples from Iberia, where preservation is poor due to the climate and the coverage often lower. Moreover, many samples are not well-dated archaeologically: several individual Beaker samples from the Netherlands and Germany are dated to ~2300–2600 BC. The oldest samples archaeologically are from the Steppe (>3000 BC); following this, most are from north-central Europe Beaker Complex contexts (Corded Ware contexts from northern Europe may be slightly older, but largely lack the R1b-M269 that traces back to the Pontic-Caspian Steppe); but it is not possible to distinguish ages of Beaker samples from north-central Europe and several from Iberia, which may be similar age or several centuries later.

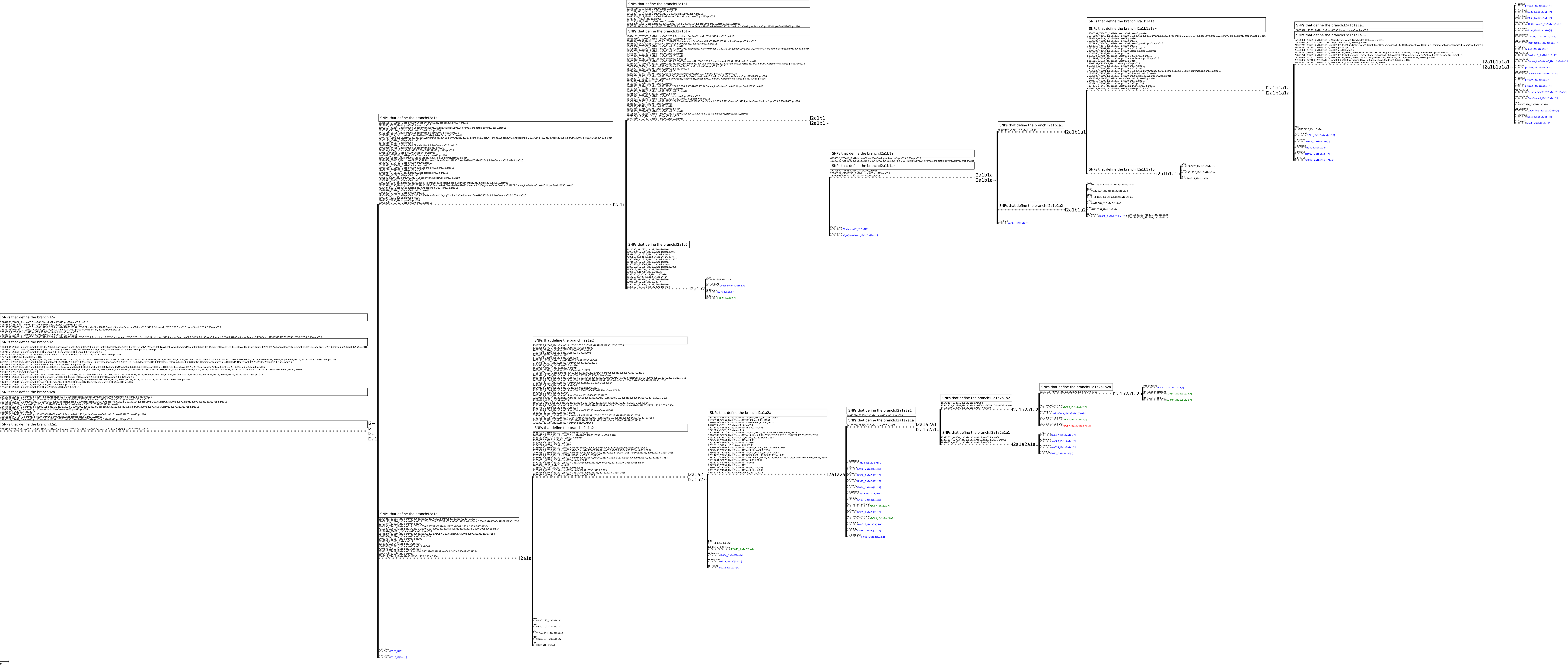

SNPs that define the branch:I2a1b1
SNPs that define the branch:I2a1b1~
SNPs that define the branch:I2a1b
SNPs that define the branch:I2a1b2
SNPs that define the branch:I2a1b1a
SNPs that define the branch:I2a1b1a~
SNPs that define the branch:I2a1b1a1
SNPs that define the branch:I2a1b1a1a
SNPs that define the branch:I2a1b1a1a~
SNPs that define the branch:I2a1b1a1a1
SNPs that define the branch:I2a1b1a1a1~
SNPs that define the branch:I2a1b1a1b
SNPs that define the branch:I2a1b1a2
SNPs that define the branch:I2~
SNPs that define the branch:I2
SNPs that define the branch:I2a
SNPs that define the branch:I2a1
SNPs that define the branch:I2a1a2
SNPs that define the branch:I2a1a2~
SNPs that define the branch:I2a1a
SNPs that define the branch:I2a1a2a
SNPs that define the branch:I2a1a2a1
SNPs that define the branch:I2a1a2a1a
SNPs that define the branch:I2a1a2a1a2
SNPs that define the branch:I2a1a2a1a1
SNPs that define the branch:I2a1a2a1a2a

Fig. S15. Phylogeny of haplogroup I2a-L460*.* A snapshot of the I2a subclade of the 1000 Genomes Project data Y-chromosome phylogenetic tree, including nine new ancient I2a genomes from the Bronze Age Links of Noltland in Orkney and the Strath Glebe Neolithic on Skye (labelled in green), alongside 51 published Mesolithic/Neolithic aDNA genomes (labelled in blue). Each node displays in black variants shared by two or more ancient genomes that are located to their most likely place on the tree. Note that the nomenclature used here follows pathPhynder in using the ISOGG 2018 nomenclature, which includes more SNPs than the ISOGG 2017 nomenclature, but for clarity we use the more familiar ISOGG 2017 in our main text and figures (*see* S6; Table S3).

Each node displayed in black shows haplogroups and variants that define the most likely phylogenetic position of each ancient samples. Following the ISOGG (2018) nomenclature, haplogroups and variants labelled with the symbol ~ indicate only an approximate location on the ISOGG tree. Note that branch lengths are not additive; they only link variants/haplogroups/samples labelled to the tree for visualisation purposes.

Each ancient sample ID is suffixed with [x], [?] or [?1/2]. The symbol [x] indicates that we do not expect a deeper haplogroup classification due to missing data – the classification should be robust. The symbol [?] indicates, by contrast, that we cannot confirm the haplogroup classification, due to missing data, and a more refined haplogroup determination might be possible. For example, the symbol [?1/2] means that the sample has missing data for all the diagnostic SNPs in one of the two next-derived haplogroups. In this case, we cannot exclude a haplogroup classification within the derived haplogroup where there is missing data. However, the presence of the ancestral state for the second next-derived haplogroup confirms that the sample does not belong to that subclade.

Note that, if we consider the effect of potential deamination, the phylogenetic position of KD059 falls on the I2a branch. However, without deamination, KD059 falls on the branch I2a1a2a1a2 alongside two other Links of Noltland samples and one from England. We labelled it here in red, to underline its uncertain phylogenetic position, but we have nevertheless left it at what we considered its most likely phylogenetic position, as I2a1a2a1a2.

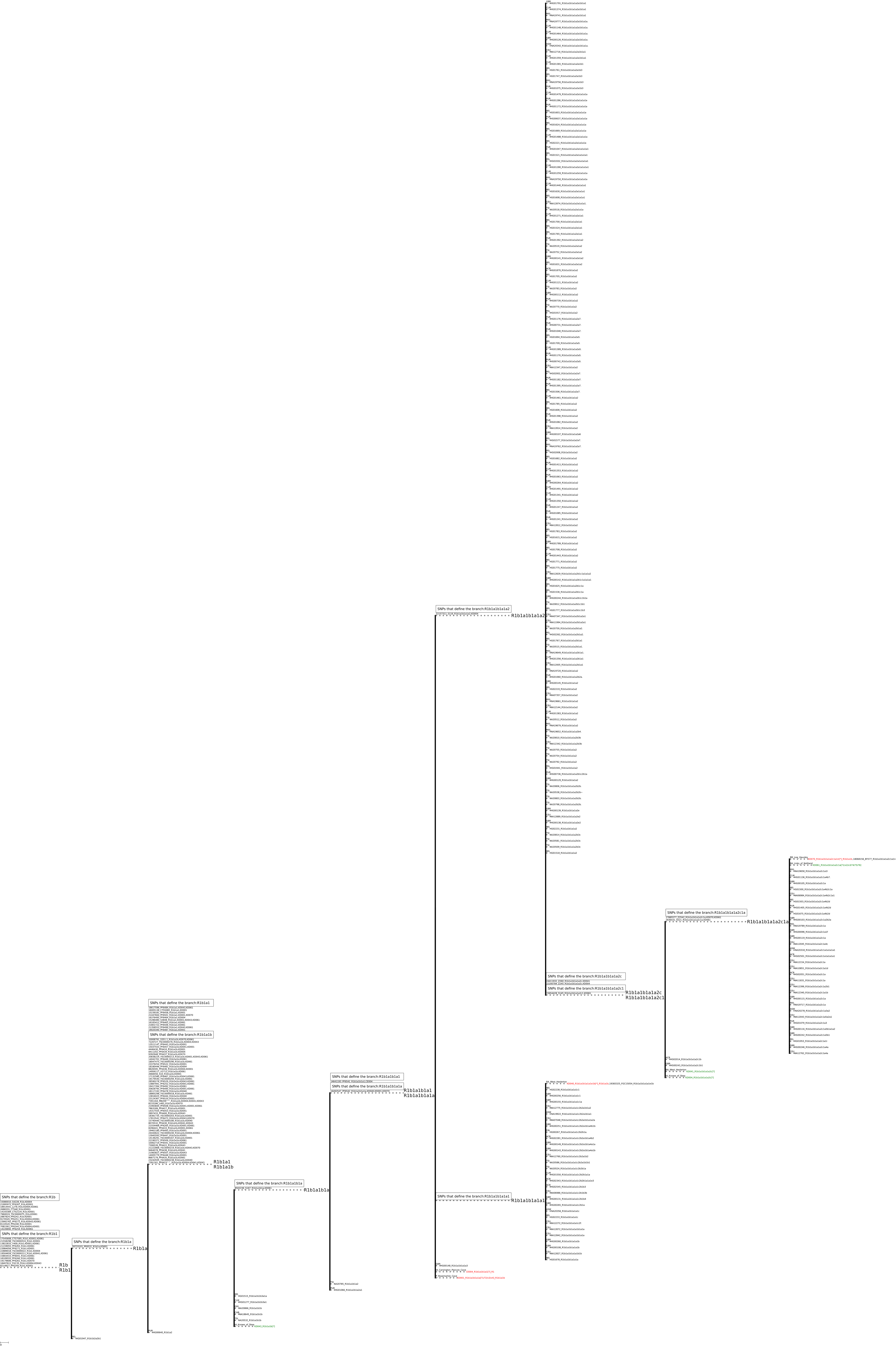

SNPs that define the branch:R1b
SNPs that define the branch:R1b1
R1b
R1b1
SNPs that define the branch:R1b1a
R1b1a
SNPs that define the branch:R1b1a1
SNPs that define the branch:R1b1a1b
R1b1a1
R1b1a1b
SNPs that define the branch:R1b1a1b1a
R1b1a1b1a
SNPs that define the branch:R1b1a1b1a1
SNPs that define the branch:R1b1a1b1a1a
R1b1a1b1a1
R1b1a1b1a1a
SNPs that define the branch:R1b1a1b1a1a2
R1b1a1b1a1a2
SNPs that define the branch:R1b1a1b1a1a1
R1b1a1b1a1a1
SNPs that define the branch:R1b1a1b1a1a2c
SNPs that define the branch:R1b1a1b1a1a2c1
R1b1a1b1a1a2c
R1b1a1b1a1a2c1
SNPs that define the branch:R1b1a1b1a1a2c1a
R1b1a1b1a1a2c1a

Fig. S16. Phylogeny of haplogroup R1b1-L278*.* A snapshot of the R1b1 subclade of the 1000 Genomes Project data Y-chromosome phylogenetic tree, including eight new ancient genomes from: Bronze Age Links of Noltland and Iron Age Knowe of Skea in Orkney; Iron Age High Pasture Cave in Skye, Hebrides; Pictish Rosemarkie Cave, Black Isle; Bronze Age West Heslerton, North Yorkshire; and Beaker burial sample from Low Hauxley, Northumberland (labelled in green). See legend to Fig. S15 for further details.

LoN	LoN prop	Pop2	Pop2 prop	SE
LoN	0.941	Bulgaria_Beli_Breyag_EBA	0.059	0.074
LoN	0.955	Bulgaria_EBA	0.045	0.051
LoN	0.923	Croatia_MBA	0.077	0.072
LoN	0.948	Hungary_BA	0.052	0.051
LoN	0.974	Hungary_Bell_Beaker	0.026	0.051
LoN	0.836	Hungary_LBA	0.164	0.107
LoN	0.942	Hungary_Mako_EBA	0.058	0.101
LoN	0.935	Iberia_BA	0.065	0.069
LoN	0.973	Iberia_Bell_Beaker	0.027	0.052
LoN	0.937	Iberia_Bell_Beaker_C	0.063	0.078
LoN	0.946	Iberia_MBA	0.054	0.063
LoN	0.959	Italy_Bell_Beaker	0.041	0.049
LoN	0.957	Italy_Remedello_BA	0.043	0.046
LoN	0.971	Jordan_EBA	0.029	0.043

Table S1. Knowe of Skea ancestry, using *qpAdm* to model Knowe of Skea as a two-way admixture of Links of Noltland and a second Bronze Age population, showing all significant *qpAdm* results.

N_e	0.025 95% boundary	0.975 95% boundary	Culture	Location	Site	Number of individuals
1760	673	7065	Neolithic	Orkney	Unstan	1
1980	1363	3039	Neolithic	Orkney	Isbister	9
2647	852	15,866	Neolithic	Orkney	Point of Cott	1
3514	1736	8876	Neolithic	Orkney	Holm of Papa Westray	4
1760	673	7065	Neolithic	Orkney	Quoyness	1
2257	1170	5248	Neolithic	Scotland	Distillery Cave	3
3018	1873	5358	Neolithic	Scotland	Raschoille Cave	8
2257	1170	5248	Neolithic	Scotland	Tulloch of Assery	3
**1048**	**483**	**2936**	**Neolithic**	**Scotland**	**Strathglebe**	**1**
2107	978	5903	BB	France	Moselle	2
2063	1411	3190	BB	Netherlands	Oostwoud	9
7946	2574	47,638	EBA	Ireland	Rathlin	3
393	234	721	EBA	Orkney	LopNess	1
**426**	**249**	**806**	**MBA**	**Orkney**	**KD064 (LoN)**	**1**
**342**	**209**	**598**	**MBA**	**Orkney**	**KD061 (LoN)**	**1**
**317**	**198**	**547**	**MBA**	**Orkney**	**KD060 (LoN)**	**1**
**518**	**292**	**1048**	**MBA**	**Orkney**	**KD049 (LoN)**	**1**
**387**	**298**	**514**	**MBA**	**Orkney**	**LoN total**	**4**
7946	2574	47,638	MBA	Scotland	Longniddry	3
3018	1565	7016	LBA	Scotland	Covsea Cave	4
**1316**	**562**	**4235**	**Iron Age**	**Orkney**	**Knowe of Skea**	**1**

Table S2. Maximum-likelihood estimates of effective population size (N_e) with 95% confidence intervals for newly reported samples (in bold) and previously published populations. All samples have over 400,000 SNPs in the 1240K SNP array.

ISOGG 2016 Y haplogroup	ISOGG 2017 Y haplogroup	ISOGG 2018 Y haplogroup
I2a-L460/PF3647/S238	I2a-L460/PF3647/S238	I2a1-L460/PF3647/S238
I2a1-P37.2/PF4004	I2a1-P37.2/PF4004	*No match*
I2a1b-M423	**I2a1b-M423***	I2a1a2-M423
I2a1b1-L161.1/S185	**I2a1b1-L161.1/S185.1***	I2a1a2a-L161.1/S185
I2a1b2-L621/S392	I2a1b2-L621/S392	I2a1a2b-L621/S392
I2a1b1a- AMM079.1/L1498.1/Y4008.1	I2a1b1a1-AMM079.1/L1498.1/Y4008.1	I2a1a2a1a-L1498.1/AMM079.1/Y4008.1
I2a1b1a1-S2703	I2a1b1a1a-S2703/FGC7126/AMM068	I2a1a2a1a1-S2703/FGC7126/AMM068
Not described	**I2a1b1a1b-A1150/Y13519***	I2a1a2a1a2-A1150/Y13519
Not described	**I2a1b1a1b1-A8742***	I2a1a2a1a2a-A8742
I2a1a-CTS595	I2a1a-CTS595	I2a1a1-CTS595
I2a1a1-M26/PF4056	I2a1a1-M26/PF4056	I2a1a1a-M26/PF4056
I2a1a1a-L672/S327	~I2a1a1a-Y3992 *(approximate match)*	*No match*
I2a2a1a1-M284	I2a2a1a1-M284	I2a1b1a1a-M284
I2a1a2-S21825	I2a1a2-S21825	I2a1a1b-S21825
I2a2-M436/P214/PF3856/S33	I2a2-M436/P214/PF3856/S33	I2a1b-M436/P214/PF3856/S33
Not described	**I2a2b-FGC29562/Y10705**	I2a1b2-FGC29562/Y10705
I2a2b-L38/S154	I2a2b1-L38/S154	I2a1b2a-L38/S154
R1b1a1a2-M269/PF6517	**R1b1a1a2-M269/PF6517****	R1b1a1b-M269/PF6517
R1b1a1a2a-L23/PF6534/S141	R1b1a1a2a-L23/PF6534/S141	R1b1a1b1-L23/PF6534/S141
R1b1a1a2a1a-L151/PF6542	**R1b1a1a2a1a-L151/PF6542**	R1b1a1b1a1a-- L151/PF6542
R1b1a1a2a1a1e1b-FGC15048	**R1b1a1a2a1a1e1b-FGC15048**	R1b1a1b1a1a1e1b-FGC15048
R1b1a1a2a1a2-P312/PF6547/S116	R1b1a1a2a1a2-P312/PF6547/S116	R1b1a1b1a1a2-P312/PF6547/S116
R1b1a1a2a1a2c-L21/M529/S145/S461/Z290	**R1b1a1a2a1a2c-S461/Z290****	R1b1a1b1a1a2c-S461/Z290
R1b1a1a2a1a2c-L21/S145/M529/S461/Z290	R1b1a1a2a1a2c1-L21/M529/S145	R1b1a1b1a1a2c1-L21/S145/M529
R1b1a1a2a1a2c1-CTS241/DF13/S521	**R1b1a1a2a1a2c1a-CTS241/DF13/S521***	R1b1a1b1a1a2c1a-CTS241/DF13/S521
R1b1a1a2a1a2c1a1-DF49/S474	R1b1a1a2a1a2c1a1a-DF49/S474	R1b1a1b1a1a2c1a1a-DF49/S474
R1b1a1a2a1a2c1a1-BY575	**R1b1a1a2a1a2c1a1n-BY575**	R1b1a1b1a1a2c1a1n-BY575

Table S3. Haplogroup classification translation guide for I2a-L460 and R1b-L23 by ISOGG, from 2016 to 2018. Haplogroups identified in the present study are shown in bold, using the ISOGG 2017 nomenclature. *Orkney Bronze Age; **Orkney Iron Age.

Dataset S1 *(separate Excel file)*

Dataset S1A. Archaeological information for newly reported samples.

Dataset S1B. Sequencing information and contamination estimates for newly reported samples.

Dataset S1C. Table of modern mtDNA haplotypes and sequence variants.

Dataset S1D. Table of symmetry *D*-statistics demonstrating that LoN samples form a single population group.

Dataset S1E. *D*-statistics for all newly reported samples, comparing them to published ancient populations.

Dataset S1F. qpAdm results modelling newly reported samples as an admixture of CHG, ANF and WHG populations.

Dataset S1G. Table of mtDNA haplotypes and sequence variants for newly reported samples.

Dataset S1H. Table of Y-chromosome haplotypes and sequence variants with both ISOGG 2017 and 2018 classifications.

Dataset S1I. Table of published samples used for genome-wide analyses.

Dataset S1J. Comparison of autosomal and X chromosomal qpAdm results for Strath Glebe to investigate sex-bias.

Dataset S1K. Table of *D*-statistics testing whether LoN and KoS form a clade to the exclusion of other Bronze Age populations.

Dataset S2 *(separate Excel file).* Maximum-parsimony tree of mitogenomes from 1856 modern Orcadians and Shetlanders, and 209 ancient mitogenomes from prehistoric Britain and Ireland.

Dataset S3 *(separate Excel file).* Most parsimonious phylogeny of ancient DNA mitogenomes belonging to haplogroup U5b2a3, including two modern mitogenomes belonging to U5b2a3b.

SI References

1. N. Card *et al.*, To cut a long story short: Formal chronological modelling for the Late Neolithic site of Ness of Brodgar, Orkney. *Eur J Archaeol* **21**, 217-263 (2017).
2. C. Richards, R. Jones, Eds., *The development of Neolithic house societies in Orkney* (Windgather Press, Oxbow Books, Oxford, 2016).
3. V. G. Childe, W. G. Grant, A Stone-Age settlement at the Braes of Rinyo, Rousay, Orkney. (First Report). *Proc Soc Antiq Scot* **78**, -31. (1939).
4. V. G. Childe, W. G. Grant, A Stone-Age settlement at the Braes of Rinyo, Rousay, Orkney. *Proc Soc Antiq Scot* **81**, 16-42 (1949).
5. H. Moore, G. Wilson, *Shifting sands: Links of Noltland, Westray: Interim report on Neolithic and Bronze Age excavations, 2007-09* (Historic Scotland, Edinburgh, 2011).
6. C. Renfrew, *Investigations in Neolithic Orkney*, Reports of the Research Committee of the Society of Antiquaries of London (Society of Antiquaries, London, 1979), vol. No. XXXVIII, pp. 234.
7. J. A. Sheridan, "A Rumsfeld reality check: what we know, what we don't know and what we don't know we don't know about the Chalcolithic in Britain and Ireland" in Is there a British Chalcolithic? People, Place and Polity in the Later Third Millennium*,* M. J. Allen, J. Gardiner, J. A. Sheridan, Eds. (Oxbow/Prehistoric Society, Oxford, 2012), vol. Prehistoric Society Research Paper 4, pp. 40–55.
8. A. Bayliss, P. Marshall, C. Richards, A. Whittle, Islands of history: the Late Neolithic timescape of Orkney. *Antiquity* **91**, 1171-1188 (2017).
9. C. J. Stevens, D. Q. Fuller, Alternative strategies to agriculture: the evidence for climatic shocks and cereal declines during the British Neolithic and Bronze Age (a reply to Bishop). *World Archaeol* **47**, 856-875 (2015).
10. L. Sims, The 'solarization' of the moon: Manipulated knowledge at Stonehenge. *Cambridge Archaeological Journal* **16**, 191-207 (2006).
11. S. Brace *et al.*, Ancient genomes indicate population replacement in Early Neolithic Britain (vol 3, pg 765, 2019). *Nat Ecol Evol* **3**, 986-987 (2019).
12. I. Olalde *et al.*, The Beaker phenomenon and the genomic transformation of northwest Europe. *Nature* **555**, 190-196 (2018).
13. H. Moore, G. Wilson, Sands of time: Domestic rituals at the Links of Noltland. *Current Archaeology* **275**, 12–19 (2013).
14. S. Fraser *et al.*, Matrilines in Neolithic cattle from Orkney, Scotland reveals complex husbandry patterns of ancestry. *J Archaeol Sci Rep* **14**, 46-54 (2017).
15. S. Fraser (2015) Mammals in Late Neolithic Orkney (with reference to mammal bone recovered from Links of Noltland, Westray). (University of Edinburgh).
16. L. E. McKenna, I. Simpson, "Thin section micromorphology of anthrosols (Area 1)" in Shifting Sands. Links of Noltland, Westray: Interim Report on Neolithic and Bronze Age Excavations, 2007-09*,* H. Moore, G. Wilson, Eds. (Historic Scotland, Edinburgh, 2011), vol. Archaeological Report 4, pp. 77-89.
17. M. Wildgoose, G. Kozikowski (2018) Strath Glebe: Isle of Skye: The excavation of a rectangular stone setting on Strath Glebe Farm 2015-18: Data Structure Report. (Phoenix Archaeology.).
18. C. Waddington, C. Bonsall, *Archaeology and environment on the North Sea littoral: a case study from Low Hauxley* (Archaeological Research Services, 2016).
19. C. Bonsall, Low Hauxley, Northumberland: Mesolithic/Bronze Age coastal site. *Proceedings of the Prehistoric Society* **50**, 398 (1984).
20. C. Haughton, D. Powlesland, *West Heslerton: The Anglian Cemetery.* , Landscape Research Centre Archaeological Monographs (English Heritage, 1999).

21. Powlesland D., H. C., H. J., Excavations at Heslerton, North Yorkshire 1978–82. *Archaeol J* **143**, 53-173 (1986).
22. H. Moore, G. Wilson, Two Orcadian cist burials: excavations at Midskaill, Egilsay, and Linga Fiold, Sandwick. *Proceedings of the Society of Antiquaries of Scotland* **125**, 237-251 (1995).
23. S. Øvrevik, "The second millennium BC and after" in The prehistory of Orkney BC 4000–1000 AD*,* C. Renfrew, Ed. (Edinburgh University Press, Edinburgh, 1985), pp. 131-149.
24. J. Waddell, *The Bronze Age Burials of Ireland.* (Galway University Press, Galway, 1990).
25. D. Powlesland, C. Haughton, and J. Hanson Excavations at Heslerton, North Yorkshire 1978–82. *Archaeol J* **143**, 53–173 (1986).
26. A. T. Chamberlain, Carsington Pasture Cave, Brassington, Derbyshire: A prehistoric burial site. *Capra* **1**, 1 (1999).
27. D. Gooney (2015) *Life and death in Iron Age Orkney: an osteoarchaeological examination of the human skeletal remains from the burial ground at Knowe of Skea, Westray.* PhD thesis, University of Edinburgh. Available at: Edinburgh Research Archive http://hdl.handle.net/1842/25744 (accessed 17/12/2021)
28. H. Moore, G. Wilson, Knowe of Skea, Westray: The 4000 year history of a remarkable place. *Orkney Archaeological Review* **3**, 55-64 (2018).
29. S. A. Birch, J. T. Mackenzie, G. Cruickshanks, *High Pasture Cave: Ritual, Memory and Identity in the Iron Age of Skye* (Oxbow Books, Oxford, 2021).
30. K. Dulias *et al.*, Maternal relationships within an Iron Age burial at the High Pasture Cave, Isle of Skye, Scotland. *J Archaeol Sci* **110**, 104978 (2019).
31. O. Lelong, Ed., *Excavations at Milla Skerra, Sandwick, Unst: Rhythms of Life in Iron Age Shetland.* (Oxbow Books, Oxford, 2019).
32. S. P. Birch, M. (2017) Rosemarkie Caves Project. Data Structure Report 2016. Archaeological Excavation in Learnie 2B Near Rosemarkie, Ross-shire. Data Structure Report. . in *Rosemarkie Caves Project.*
33. R. Pinhasi *et al.*, Optimal Ancient DNA Yields from the Inner Ear Part of the Human Petrous Bone. *Plos One* **10**, e0129102 (2015).
34. D. Y. Yang, B. Eng, J. S. Waye, J. C. Dudar, S. R. Saunders, Improved DNA extraction from ancient bones using silica-based spin columns. *Am J Phys Anthropol* **105**, 539–543 (1998).
35. D. E. MacHugh, C. J. Edwards, J. F. Bailey, D. R. Bancroft, D. G. Bradley, The extraction and analysis of ancient DNA from bone and teeth: a survey of current methodologies. *Anc Biomol* **3**, 81–102 (2000).
36. M. Meyer, M. Kircher, Illumina sequencing library preparation for highly multiplexed target capture and sequencing. *Cold Spring Harbor Protocols 2010* **pdb.prot5448** (2010).
37. S. Andrews (2010) FastQC: a quality control tool for high throughput sequence data.
38. M. Schubert, S. , S. Lindgreen, L. Orlando, AdapterRemoval v2: rapid adapter trimming, identification, and read merging. *BMC Res Notes* **9**, 88 (2016).
39. H. Li, R. Durbin, Fast and accurate short read alignment with Burrows–Wheeler transform. *Bioinformatics* **25**, 1754–1760 (2009).
40. R. M. Andrews *et al.*, Reanalysis and revision of the Cambridge reference sequence for human mitochondrial DNA. *Nature Genet.* **23**, 147 (1999).
41. M. Schubert *et al.*, Improving ancient DNA read mapping against modern reference genomes. *BMC Genomics* **13**, 178 (2012).
42. H. Li *et al.*, The Sequence Alignment/Map format and SAMtools. *Bioinformatics* **25**, 2078-2079 (2009).
43. K. Okonechnikov, A. Conesa, F. García-Alcalde, Qualimap 2: advanced multi-sample quality control for high-throughput sequencing data. *Bioinformatics* **32**, 292-294 (2015).
44. H. Jónsson, A. Ginolhac, M. Schubert, P. L. F. Johnson, L. Orlando, mapDamage2.0: fast approximate Bayesian estimates of ancient DNA damage parameters. *Bioinformatics* **29**, 1682-1684 (2013).

45. G. Jun *et al.*, Detecting and estimating contamination of human DNA samples in sequencing and array-based genotype data. *Am J Hum Genet* **91**, 839-848 (2012).
46. T. Gunther *et al.*, Population genomics of Mesolithic Scandinavia: Investigating early postglacial migration routes and high-latitude adaptation. *PLoS Biol* **16**, e2003703 (2018).
47. F. Sanchez-Quinto *et al.*, Megalithic tombs in western and northern Neolithic Europe were linked to a kindred society. *Proc Natl Acad Sci USA* **116**, 9469-9474 (2019).
48. S. Schiffels *et al.*, Iron Age and Anglo-Saxon genomes from East England reveal British migration history. *Nat Commun* **7**, 10408 (2016).
49. G. Renaud, V. Slon, A. T. Duggan, J. Kelso, Schmutzi: estimation of contamination and endogenous mitochondrial consensus calling for ancient DNA. *Genome Biol* **16**, 224 (2015).
50. P. Skoglund, J. Stora, A. Gotherstrom, M. Jakobsson, Accurate sex identification of ancient human remains using DNA shotgun sequencing. *J Archaeol Sci* **40**, 4477-4482 (2013).
51. P. Skoglund, E. Ersmark, E. Palkopoulou, L. Dalen, Ancient Wolf Genome Reveals an Early Divergence of Domestic Dog Ancestors and Admixture into High-Latitude Breeds. *Curr Biol* **25**, 1515-1519 (2015).
52. H. Weissensteiner *et al.*, HaploGrep 2: mitochondrial haplogroup classification in the era of high-throughput sequencing. *Nucleic Acids Res* **44**, W58-63 (2016).
53. M. van Oven, M. Kayser, Updated comprehensive phylogenetic tree of global human mitochondrial DNA variation. *Hum Mutat* **30**, E386-394 (2009).
54. J. T. Robinson, H. Thorvaldsdottir, A. M. Wenger, A. Zehir, J. P. Mesirov, Variant Review with the Integrative Genomics Viewer. *Cancer Res* **77**, e31-e34 (2017).
55. A. McKenna *et al.*, The Genome Analysis Toolkit: a MapReduce framework for analyzing next-generation DNA sequencing data. *Genome Research* **20**, 1297-1303 (2010).
56. A. Keller *et al.*, New insights into the Tyrolean Iceman's origin and phenotype as inferred by whole-genome sequencing. *Nat Commun* **3**, 698 (2012).
57. M. Meyer *et al.*, A high-coverage genome sequence from an archaic Denisovan individual. *Science* **338**, 222-226 (2012).
58. N. Patterson *et al.*, Ancient admixture in human history. *Genetics* **192**, 1065-1093 (2012).
59. J. K. Pickrell *et al.*, The genetic prehistory of southern Africa. *Nat Commun* **3**, 1143 (2012).
60. Q. Fu *et al.*, Genome sequence of a 45,000-year-old modern human from western Siberia. *Nature* **514**, 445-449 (2014).
61. C. Gamba *et al.*, Genome flux and stasis in a five millennium transect of European prehistory. *Nat Commun* **5**, 5257 (2014).
62. I. Lazaridis *et al.*, Ancient human genomes suggest three ancestral populations for present-day Europeans. *Nature* **513**, 409-413 (2014).
63. M. Raghavan *et al.*, Upper Palaeolithic Siberian genome reveals dual ancestry of Native Americans. *Nature* **505**, 87-91 (2014).
64. M. Rasmussen *et al.*, The genome of a Late Pleistocene human from a Clovis burial site in western Montana. *Nature* **506**, 225-229 (2014).
65. A. Seguin-Orlando *et al.*, Genomic structure in Europeans dating back at least 36,200 years. *Science* 10.1126/science.aaa0114 (2014).
66. P. Skoglund *et al.*, Genomic diversity and admixture differs for Stone-Age Scandinavian foragers and farmers. *Science* **344**, 747-750 (2014).
67. C. Genomes Project *et al.*, A global reference for human genetic variation. *Nature* **526**, 68-74 (2015).
68. M. E. Allentoft *et al.*, Population genomics of Bronze Age Eurasia. *Nature* **522**, 167-172 (2015).
69. E. R. Jones *et al.*, Upper Palaeolithic genomes reveal deep roots of modern Eurasians. *Nat Commun* **6**, 8912 (2015).
70. T. Gunther *et al.*, Ancient genomes link early farmers from Atapuerca in Spain to modern-day Basques. *Proc Natl Acad Sci USA* **112**, 11917-11922 (2015).

71. I. Mathieson *et al.*, Genome-wide patterns of selection in 230 ancient Eurasians. *Nature* **528**, 499-503 (2015).
72. M. G. Llorente *et al.*, Ancient Ethiopian genome reveals extensive Eurasian admixture in Eastern Africa. *Science* **350**, 820-822 (2015).
73. M. Raghavan *et al.*, Genomic evidence for the Pleistocene and recent population history of Native Americans. *Science* **349**, aab3884 (2015).
74. F. Broushaki *et al.*, Early Neolithic genomes from the eastern Fertile Crescent. *Science* **353**, 499-503 (2016).
75. L. M. Cassidy *et al.*, Neolithic and Bronze Age migration to Ireland and establishment of the insular Atlantic genome. *Proc Natl Acad Sci USA* **113**, 368-373 (2016).
76. Q. Fu *et al.*, The genetic history of Ice Age Europe. *Nature* **534**, 200-205 (2016).
77. Z. Hofmanová *et al.*, Early farmers from across Europe directly descended from Neolithic Aegeans. *Proc Natl Acad Sci USA* **113**, 6886-6891 (2016).
78. G. M. Kilinc *et al.*, The Demographic Development of the First Farmers in Anatolia. *Curr Biol* **26**, 2659-2666 (2016).
79. I. Lazaridis *et al.*, Genomic insights into the origin of farming in the ancient Near East. *Nature* **536**, 419-424 (2016).
80. R. Martiniano *et al.*, Genomic signals of migration and continuity in Britain before the Anglo-Saxons. *Nat Commun* **7**, 10326 (2016).
81. G. Gonzalez Fortes *et al.*, Paleogenomic Evidence for Multi-generational Mixing between Neolithic Farmers and Mesolithic Hunter-Gatherers in the Lower Danube Basin. *Curr Biol* **27**, 1801-+ (2017).
82. I. Lazaridis *et al.*, Genetic origins of the Minoans and Mycenaeans. *Nature* **548**, 214-218 (2017).
83. M. Lipson *et al.*, Parallel palaeogenomic transects reveal complex genetic history of early European farmers. *Nature* **551**, 368-372 (2017).
84. L. Saag *et al.*, Extensive farming in Estonia started through a sex-biased migration from the Steppe. *Curr Biol* **27**, 2185-2193 (2017).
85. P. B. Damgaard *et al.*, 137 ancient human genomes from across the Eurasian steppes. *Nature* **557**, 369-374 (2018).
86. R. Fregel *et al.*, Ancient genomes from North Africa evidence prehistoric migrations to the Maghreb from both the Levant and Europe. *Proc Natl Acad Sci USA* **115**, 6774-6779 (2018).
87. I. Mathieson *et al.*, The genomic history of southeastern Europe. *Nature* **555**, 197-203 (2018).
88. C. Valdiosera *et al.*, Four millennia of Iberian biomolecular prehistory illustrate the impact of prehistoric migrations at the far end of Eurasia. *Proc Natl Acad Sci USA* **115**, 3428-3433 (2018).
89. M. van de Loosdrecht *et al.*, Pleistocene North African genomes link Near Eastern and sub-Saharan African human populations. *Science* **360**, 548-552 (2018).
90. V. M. Narasimhan *et al.*, The formation of human populations in South and Central Asia. *Science* **365** (2019).
91. I. Olalde *et al.*, The genomic history of the Iberian Peninsula over the past 8000 years. *Science* **363**, 1230-1234 (2019).
92. S. Mallick *et al.*, The Simons Genome Diversity Project: 300 genomes from 142 diverse populations. *Nature* **538**, 201-206 (2016).
93. R. Martiniano *et al.*, The population genomics of archaeological transition in west Iberia: Investigation of ancient substructure using imputation and haplotype-based methods. *Plos Genet* **13** (2017).
94. J. M. M. Kuhn, M. Jakobsson, T. Gunther, Estimating genetic kin relationships in prehistoric populations. *Plos One* **13** (2018).

95. D. H. Alexander, J. Novembre, K. Lange, Fast model-based estimation of ancestry in unrelated individuals. *Genome Res* **19**, 1655-1664 (2009).
96. C. C. Chang *et al.*, Second-generation PLINK: rising to the challenge of larger and richer datasets. *Gigascience* **4**, 7 (2015).
97. N. Patterson, A. L. Price, D. Reich, Population structure and eigenanalysis. *PLoS Genet* **2**, e190 (2006).
98. A. L. Price *et al.*, Principal components analysis corrects for stratification in genome-wide association studies. *Nat Genet* **38**, 904-909 (2006).
99. D. Gurdasani *et al.*, The African Genome Variation Project shapes medical genetics in Africa. *Nature* **517**, 327-332 (2015).
100. A. Sheridan, Movement, Exchange and Identity in Europe in the 2nd and 1st Millennia BC: beyond frontiers. *Int J Naut Archaeol* **49**, 205-207 (2020).
101. L. M. Cassidy *et al.*, A dynastic elite in monumental Neolithic society. *Nature* **582**, 384-388 (2020).
102. H. Ringbauer, J. Novembre, M. Steinrücken, Human Parental Relatedness through Time - Detecting Runs of Homozygosity in Ancient DNA. *bioRxiv* 10.1101/2020.05.31.126912, 2020.2005.2031.126912 (2020).
103. R. McQuillan *et al.*, Runs of homozygosity in European populations. *Am J Hum Genet* **83**, 359-372 (2008).
104. S. M. Kerr *et al.*, An actionable KCNH2 Long QT Syndrome variant detected by sequence and haplotype analysis in a population research cohort. *Sci Rep* **9**, 10964 (2019).
105. G. A. Van der Auwera, B. D. O'Connor, *Genomics in the Cloud: Using Docker, GATK, and WDL in Terra* (O'Reilly Media, 2020).
106. M. Halachev *et al.*, Increased ultra-rare variant load in an isolated Scottish population impacts exonic and regulatory regions. *Plos Genet* **15**, e1008480 (2019).
107. A. Peltzer *et al.*, EAGER: efficient ancient genome reconstruction. *Genome Biol* **17**, 60 (2016).
108. D. M. Fernandes *et al.*, A genomic Neolithic time transect of hunter-farmer admixture in central Poland. *Sci Rep* **8**, 14879 (2018).
109. W. Haak *et al.*, Massive migration from the steppe was a source for Indo-European languages in Europe. *Nature* **522**, 207-211 (2015).
110. S. Brunel *et al.*, Ancient genomes from present-day France unveil 7,000 years of its demographic history. *Proc Natl Acad Sci USA* **117**, 12791-12798 (2020).
111. M. Rivollat *et al.*, Ancient genome-wide DNA from France highlights the complexity of interactions between Mesolithic hunter-gatherers and Neolithic farmers. *Sci Adv* **6**, eaaz5344 (2020).
112. M. D. Costa *et al.*, A substantial prehistoric European ancestry amongst Ashkenazi maternal lineages. *Nat Commun* **4**, 2543 (2013).
113. A. J. Drummond, A. Rambaut, BEAST: Bayesian evolutionary analysis by sampling trees. *BMC Evolutionary Biology* **7**, 214 (2007).
114. M. Krzewinska *et al.*, Ancient genomes suggest the eastern Pontic-Caspian steppe as the source of western Iron Age nomads. *Sci Adv* **4** (2018).
115. C. Knipper *et al.*, Female exogamy and gene pool diversification at the transition from the Final Neolithic to the Early Bronze Age in central Europe. *Proc Natl Acad Sci USA* **114**, 10083-10088 (2017).
116. D. M. Behar *et al.*, A "Copernican" reassessment of the human mitochondrial DNA tree from its root. *Am J Hum Genet* **90**, 675-684 (2012).
117. M. Silva *et al.*, A genetic chronology for the Indian Subcontinent points to heavily sex-biased dispersals. *BMC Evolutionary Biology* **17**, 88 (2017).
118. A. Goldberg, T. Gunther, N. A. Rosenberg, M. Jakobsson, Ancient X chromosomes reveal contrasting sex bias in Neolithic and Bronze Age Eurasian migrations. *Proc Natl Acad Sci USA* **114**, 2657-2662 (2017).

119. B. D. S. Rui Martiniano, Pille Hallast, Richard Durbin, Placing ancient DNA sequences into reference phylogenies. *BioRXiv* **preprint** (2021).
120. G. D. Poznik *et al.*, Punctuated bursts in human male demography inferred from 1,244 worldwide Y-chromosome sequences. *Nature Genet.* **48**, 593-599 (2016).
121. I. I. S. o. G. Genealogy (2018) Y-DNA haplogroup tree 2018, version: 13.307.
122. D. Adamov, V. Guryanov, S. Karzhavin, V. Tagankin, V. Urasin, Defining a new rate constant for Y-chromosome SNPs based on full sequencing data. *Russian Journal of Genetic Genealogy (Русская версия)* **7**, 68–89 (2015).
123. J. Huerta-Cepas, F. Serra, P. Bork, ETE 3: Reconstruction, analysis, and visualization of phylogenomic data. *Mol Biol Evol* **33**, 1635-1638 (2016).
124. G. B. Busby *et al.*, The peopling of Europe and the cautionary tale of Y chromosome lineage R-M269. *Proc R Soc Lond B Biol Sci* **279**, 884-892 (2011).
125. M. Silva *et al.*, "One upon the time in the West: The archaeogenetics of Celtic origins" in Exploring Celtic origins: New ways forward in archaeology, linguistics, and genetics, B. Cunliffe, J. T. Koch, Eds. (Oxbow, Oxford, 2019), pp. 153-191.
126. C. Batini *et al.*, Large-scale recent expansion of European patrilineages shown by population resequencing. *Nat Commun* **6**, 7152 (2015).
127. J. F. Wilson *et al.*, Genetic evidence for different male and female roles during cultural transitions in the British Isles. *Proc Natl Acad Sci USA* **98**, 5078-5083 (2001).
128. C. Capelli *et al.*, A Y chromosome census of the British Isles. *Curr Biol* **13**, 979–984 (2003).
129. M. G. Thomas, M. P. H. Stumpf, H. Harke, Evidence for an apartheid-like social structure in early Anglo-Saxon England. *Proc R Soc Lond B Biol Sci* **273**, 2651-2657 (2006).
130. H. Malmstrom *et al.*, The genomic ancestry of the Scandinavian Battle Axe Culture people and their relation to the broader Corded Ware horizon. *Proc R Soc Lond B Biol Sci* **286**, 0191528 (2019).
131. A. Juras *et al.*, Mitochondrial genomes reveal an east to west cline of steppe ancestry in Corded Ware populations. *Sci Rep* **8**, 11603 (2018).
132. Q. Bourgeois, E. Kroon, The impact of male burials on the construction of Corded Ware identity: Reconstructing networks of information in the 3rd millennium BC. *Plos One* **12**, e0185971 (2017).
133. K. Kristiansen *et al.*, Re-theorising mobility and the formation of culture and language among the Corded Ware Culture in Europe. *Antiquity* **91**, 334-347 (2017).
134. H. Schroeder *et al.*, Unraveling ancestry, kinship, and violence in a Late Neolithic mass grave. *Proc Natl Acad Sci USA* **116**, 10705-10710 (2019).
135. K. M. Frei *et al.*, Tracing the dynamic life story of a Bronze Age Female. *Sci Rep* **5**, 10431 (2015).
136. D. W. Anthony, *The horse, the wheel, and language: how Bronze-Age riders from the Eurasian steppes shaped the modern world* (Princeton University Press, Princeton, 2007).
137. W. Haak *et al.*, Ancient DNA, Strontium isotopes, and osteological analyses shed light on social and kinship organization of the Later Stone Age. *Proc Natl Acad Sci USA* **105**, 18226-18231 (2008).
138. A. Mittnik *et al.*, Kinship-based social inequality in Bronze Age Europe. *Science* **366**, 731-734 (2019).
139. J. P. Mallory, *In search of the Indo-Europeans* (Thames and Hudson, London, 1989).
140. A. Szecsenyi-Nagy *et al.*, The maternal genetic make-up of the Iberian Peninsula between the Neolithic and the Early Bronze Age. *Sci Rep* **7**, 15644 (2017).
141. M. Lacan *et al.*, Ancient DNA reveals male diffusion through the Neolithic Mediterranean route. *Proc Natl Acad Sci USA* **108**, 9788-9791 (2011).
142. R. A. Bentley *et al.*, Community differentiation and kinship among Europe's first farmers. *Proc Natl Acad Sci USA* **109**, 9326-9330 (2012).
143. F. W. Marlowe, Marital residence among foragers. *Current Anthropology* **45**, 277-284 (2004).

144. V. Villalba-Mouco *et al.*, Survival of Late Pleistocene Hunter-Gatherer Ancestry in the Iberian Peninsula. *Curr Biol* **29**, 1169-1177 e1167 (2019).
145. M. L. Antonio *et al.*, Ancient Rome: A genetic crossroads of Europe and the Mediterranean. *Science* **366**, 708-714 (2019).
146. A. Bayliss, F. Healy, A. Whittle, *Gathering time: Dating the Early Neolithic enclosures of southern Britain and Ireland* (Oxbow Books, Oxford, 2011).
147. M. Peričić *et al.*, High-resolution phylogenetic analysis of southeastern Europe traces major episodes of paternal gene flow among Slavic populations. *Mol Biol Evol* **22**, 1964–1975 (2005).
148. P. Francalacci *et al.*, Low-pass DNA sequencing of 1200 Sardinians reconstructs European Y chromosome phylogeny. *Science* **341**, 565-569 (2013).

Members of the Scottish Genomes Partnership: Tim J. Aitman, Zosia Miedzybrodzka, Nicola Williams, Alison Meynert, Andrew V Biankin, Javier Santoyo-Lopez.
